# Supplementary material for: Treatment of unresectable locally advanced pancreatic cancer with percutaneous irreversible electroporation (IRE) following initial systemic chemotherapy (LAP-PIE) trial: study protocol for a feasibility randomised controlled trial
Source: BMJ Open. 2022 May 11;12(5):e050166. doi: 10.1136/bmjopen-2021-050166 (PMC9109032; doi:10.1136/bmjopen-2021-050166)
Supplement: Author's manuscript [file bmjopen-2021-050166.draft_revisions.pdf]

BMJ Open

BMJ Open is committed to open peer review. As part of this commitment we make the peer review history of every article we publish publicly available.

When an article is published we post the peer reviewers' comments and the authors' responses online. We also post the versions of the paper that were used during peer review. These are the versions that the peer review comments apply to.

The versions of the paper that follow are the versions that were submitted during the peer review process. They are not the versions of record or the final published versions. They should not be cited or distributed as the published version of this manuscript.

BMJ Open is an open access journal and the full, final, typeset and author-corrected version of record of the manuscript is available on our site with no access controls, subscription charges or pay-per-view fees (http://bmjopen.bmj.com).

If you have any questions on BMJ Open's open peer review process please email info.bmjopen@bmj.com

BMJ Open

Treatment of unresectable Locally Advanced Pancreatic cancer with Percutaneous Irreversible Electroporation (IRE) following initial systemic chemotherapy (LAP-PIE) trial: Study protocol for a feasibility randomised controlled trial.

Journal:	*BMJ Open*
Manuscript ID	bmjopen-2021-050166
Article Type:	Protocol
Date Submitted by the Author:	11-Feb-2021
Complete List of Authors:	Rai, Zainab; University College London, Division of Surgery and Interventional Science; Wellcome / EPSRC Centre for Interventional and Surgical Sciences (WEISS),<br>Ranieri, Veronica; Tavistock and Portman NHS Foundation Trust, Research & Development; University College London, Science & Technology Studies<br>Palmer, D. H.; University of Liverpool<br>Littler, Peter; Freeman Hospital<br>Ghaneh, Pauleh; University of Liverpool<br>Gurusamy, Kurinchi; University College London,<br>Manas, Derek; Newcastle Upon Tyne Hospitals NHS Trust<br>Pizzo, Elena; University College London, Applied Health Research<br>Psarelli, Eftychia; University of Liverpool, Liverpool Clinical Trials Centre<br>Davidson, Brian; Royal Free London NHS Foundation Trust, HPB Surgery
Keywords:	Adult oncology < ONCOLOGY, SURGERY, Pancreatic surgery < SURGERY, Interventional radiology < RADIOLOGY & IMAGING, Clinical trials < THERAPEUTICS

SCHOLARONE™
Manuscripts

Author Declaration

Research Ethics

The trial protocol has received the favourable opinion of the Brent Research Ethics Committee (REC).

Competing interests

We wish to confirm that there are no known conflicts of interest associated with this publication and there has been no significant financial support for this work that could have influenced its outcome.

Consent for publication

Not applicable

Availability of Data and Material

Not applicable

Funding

The LAP-PIE trial is funded by the National Institute for Health Research (NIHR) under its Research for Patient Benefit (RfPB) Programme (PB-PG-1217-20042).

EP is supported by the National Institute for Health Research ARC North Thames. The views expressed in this publication are those of the author(s) and not necessarily those of the National Institute for Health Research or the Department of Health and Social Care.

Authorship

All listed authors meet the ICMJE criteria. We attest that all authors contributed significantly to the creation of this manuscript, each having fulfilled criteria as established by the ICMJE.

We confirm that the manuscript has been read and approved by all named authors.

We confirm that the order of authors listed in the manuscript has been approved by all named authors.

Acknowledgement

This project is funded by the National Institute for Health Research (NIHR) under its Research for Patient Benefit (RfPB) Programme (PB-PG-1217-20042). The views expressed are those of the author(s) and not necessarily those of the NIHR or the Department of Health and Social Care.

Word count: 4517

For peer review only

Title

Treatment of unresectable **L**ocally **A**dvanced **P**ancreatic cancer with **P**ercutaneous **I**rreversible **E**lectroporation (IRE) following initial systemic chemotherapy (LAP-PIE) trial: Study protocol for a feasibility randomised controlled trial.

ZL Rai, V Ranieri, D Palmer, P Littler, P Ghaneh, K Gurusamy, D Manas, E Pizzo, EE Psarelli, Davidson BR and the UK LAP-PIE study group

Corresponding author: ZL Rai email: Zainab.rai.19@ucl.ac.uk

Author Contributions

ZLR: manuscript preparation. VR: trial management. DP: protocol design, trial management and co-applicant. PL: protocol design, trial management and co-applicant. EEP: statistical analysis plan. KG: major contribution to design and funding application. DM: protocol design, trial management and co-applicant. PG: protocol design, trial management and co-applicant. EP: health economic analysis design. BRD: principal investigator and grant holder, overall trial management, study design and review of manuscript. All authors read and approved the final manuscript.

Keywords

Locally advanced pancreatic cancer, Irreversible electroporation, Feasibility study, Randomised controlled trial, qualitative assessment

Strengths and limitations of this study

- Locally advanced pancreatic cancer (LAPC) accounts for 30% of patients presenting with pancreatic cancer. Irreversible Electroporation (IRE) is a novel cancer treatment that may improve survival and quality of life in LAPC.

For peer review only - http://bmjopen.bmj.com/site/about/guidelines.xhtml

Retrospective and prospective clinical studies on pancreas cancer IRE have suggested a survival benefit. Evidence from randomised controlled trails (RCT) is needed to corroborate the beneficial impact of IRE in pancreatic cancer currently reported in selected patient cohorts.

- This trial is the first randomised comparison of IRE therapy with chemotherapy versus chemotherapy alone in patients with LAPC and will aim to evaluate the feasibility of recruitment of LAPC patients across multiple pancreas cancer specialist units in the UK. The results will give a clearer indication on the impact, if any, of IRE on LAPC.
- As a feasibility study, it is not powered to show effectiveness however it is likely that the data will form the basis of a cost effectiveness application for a larger multi-centre trial. Experience in optimising recruitment, site set up and developing local radiology expertise will be crucial for the success of the subsequent trial which will be powered from data collected during this feasibility study.

For peer review only

Abstract

Background

Approximately 30% of patients with pancreas cancer have unresectable locally advanced disease, which is currently treated with systemic chemotherapy. A new treatment option of Irreversible Electroporation (IRE) has been used for these patients since 2005. Cohort studies suggest that IRE confers a survival advantage, but with significant procedure related complications. Selection bias may account for improved survival and there have been no prospective randomised trials evaluating the harms and benefits of therapy. The aim of this trial is to evaluate the feasibility of a randomised comparison of IRE therapy with chemotherapy versus chemotherapy alone in patients with locally advanced pancreatic cancer.

Methods and analysis

Eligible patients with locally advanced pancreatic cancer who have undergone first line FOLFIRINOX chemotherapy will be randomised to receive either a single session of IRE followed by (if indicated) further chemotherapy or to chemotherapy alone (standard of care). Fifty patients from seven specialist pancreas centres in the UK will be recruited over a period of 15 months. Trial follow up will be 12 months. The primary outcome measure is ability to recruit. Secondary objectives include practicality and technical success of treatment, acceptability of treatment to patients and clinicians and safety of treatment. A qualitative study has been incorporated to evaluate the patient and clinician perspective of the LAP-PIE trial. It is likely that the data obtained will guide the structure, the primary outcome measure, the power, and the duration of a subsequent multi-centre RCT aimed at establishing the cost effectiveness of pancreas IRE therapy.

For peer review only - http://bmjopen.bmj.com/site/about/guidelines.xhtml

Ethics and Dissemination

The protocol has received a favourable opinion by London-Brent Research Ethics Committee.

Results will be analysed following completion of trial recruitment and follow up.Results will be presented to international conferences with an interest in oncology, hepato-pancreatico-biliary surgery and interventional radiology and be published in a peer-reviewed journal.

Introduction

Pancreas cancer and LAPC

Pancreatic cancer (PC) is one of the deadliest cancers worldwide. It has a five-year survival rate of less than 6% and an increasing incidence (1). The low survival rate is mainly due to late diagnosis, as PC often remains asymptomatic until it becomes advanced. Surgery (pancreatic resection) is the only potentially curative treatment. However, only 10 to 20% of patients are eligible for surgery (2). Approximately 30% of all patients have locally advanced pancreatic cancer (LAPC) at diagnosis and are not considered for surgical resection (3).

Chemotherapy for LAPC

Systemic chemotherapy is the standard treatment for unresectable pancreatic cancer (4). Despite advances in drug therapy for pancreas cancer over the last 20 years the overall survival (OS) and progression free survival (PFS) of these patients has not significantly improved (5). Furthermore, a meta-analysis reported that chemoradiation is not superior to chemotherapy alone in patients with LAPC, with comparable overall survival and higher rates of grade 3–4 adverse events (6).

In the UK, NICE guidelines, published in 2018 recommend systemic combination chemotherapy for both locally unresectable and metastatic pancreatic cancer (7). NICE recommends first-line combination chemotherapy with 5-FluoroUracil, Leucovorin, Irinotecan and Oxaliplatin (FOLFIRINOX) in fit patients (Eastern Cooperative Oncology Group (ECOG) performance status of 0-1). In patients with co-morbidities and restricted activities, or those unable to tolerate this regime, NICE recommends gemcitabine alone (6). Other NICE recommended combinations include Nab-paclitaxel and gemcitabine combined with capecitabine (GemCap) (7).

For peer review only

Although FOLFIRINOX shows the best objective response in pancreatic cancer (8, 9) it is associated with more frequent and severe side effects, including neutropenia, diarrhoea and peripheral neuropathy (10).

First Line Chemotherapy

The timing of chemotherapy in relation to IRE therapy may influence outcome. Studies where IRE has been delivered before chemotherapy have reported only modest increases in median OS (11, 12) whereas studies evaluating the impact of IRE after induction treatment report an overall survival of 27 months (13). This may be because chemotherapy responders who have better outcomes are being selected to undergo IRE or alternatively it may suggest that chemotherapy modifies the tumour microenvironment making it more sensitive to IRE.

Repeat staging after first line chemotherapy and possibility of resection

Staging is repeated following first line chemotherapy for LAPC and imaging is reviewed once again regarding suitability for resection. In patients with locally advanced disease undergoing first line treatment with FOLFIRINOX, 25.9% underwent resection, and in this cohort, 78.4% had R0 resection, however the long-term outcomes for these patients is not reported in the studies, and there is still a possibility of recurrence albeit at later date. (14).

Locally advanced and borderline unresectable LAPC

The definition of borderline resectable disease is controversial (15), whereas the definition of unresectable locally advanced disease has clear consensus (16).

For peer review only

Therefore, the LAP-PIE trial will only involve patients with locally advanced disease fulfilling published criteria.

Second line chemotherapy for LAPC

NICE recommends that patients who have previously undergone FOLFIRINOX therapy as first line should be offered Gemcitabine based second-line chemotherapy (17). Other agents used in second line treatments include Capecitabine and combination therapy with Gemcitabine and Capecitabine (18). If new agents for second line therapy are approved by NICE prior to or during the LAP-PIE trial these can be offered in the second line chemotherapy arm and following IRE therapy if considered appropriate by the treating oncologist.

IRE for LAPC

Irreversible electroporation is a novel ablative method for treating solid cancers (19). IRE treatment destroys cancer cells using electricity. It can be delivered via an open approach involving a laparotomy and placement of probes around the cancer or via a percutaneous approach which requires image guidance, via CT or ultrasound guidance. Both methods require the patient to be under general anaesthesia with neuromuscular blockade. A single study using propensity score matching reported a median OS of 21.6 months in patients with LAPC treated with induction chemotherapy followed by IRE (20). A systematic review on pancreas IRE therapy from 2019 reported an OS from diagnosis or treatment of up to 27 months and a morbidity rate of 30% (13).

Complications of pancreas IRE therapy

Reported complications following pancreas IRE include pancreatic fistula, venous and arterial thrombosis, arterial pseudoaneurysm and pancreatic abscess (13). The average morbidity rate in the studies examined is 34%, but can reach up to

For peer review only

62% (21) The average rate of severe complications (defined as greater than Grade III on the Clavien-Dindo scale) following IRE is 8% but can be as high as 44% (22). The average procedure related mortality rate in these studies is 3% (12).

There are significant variations in the percentage and severity of complications reported. This difference may be due to the case selection, whether IRE is carried out during surgery or electively in the IR suite, the treatment algorithm, the completeness of reporting from treatment centres or the experience of the radiologist and/or centre. Only two studies specifically mention the experience of clinicians delivering IRE (12, 23) and currently, there is no data on the number of IRE procedures performed and the relationship with outcomes.

IRE is a palliative option and therefore its impact on patient's quality of life (QoL) is an essential consideration. A single-centre prospective study evaluating the QoL pre and post IRE in patients with LAPC demonstrated that IRE had no adverse effect on QoL however, concomitant treatment with chemotherapy after IRE was not taken into account and may confound the results (24). Currently there are no recognised QoL scores that compare IRE with no treatment or systemic chemotherapy. The data regarding the improvement in QoL in LAPC undergoing IRE is weak and needs further exploration.

Aim of the treatment of unresectable Locally Advanced Pancreatic cancer with Percutaneous Irreversible Electroporation (IRE) following initial systemic chemotherapy (LAP-PIE) trial

The current information available in the literature regarding the use of IRE in the treatment of LAPC is from retrospective and prospective cohort studies. A prospective randomised controlled trial is required to demonstrate cost effectiveness

For peer review only

of pancreas IRE therapy. The LAP-PIE trial is a feasibility trial to determine whether patients would be willing to have pancreas IRE treatment, whether they would accept randomisation to IRE or standard of care chemotherapy, whether they would comply with treatment and study data collection and to collect preliminary data on efficacy end points and costs with which to design a subsequent trial on cost effectiveness.

Methods and analysis

Study design and setting

The LAP-PIE trial is a UK multi-centre, feasibility study that will recruit 50 patients with LAPC in whom the disease has remained localised and unresectable following FOLFIRINOX chemotherapy. All participants will be offered further chemotherapy according to standard centre protocols and half will also be offered IRE. It is an open-label study. Patient enrolment is expected to last 15 months assuming a recruitment rate of 0.8 patients per site per month over 7 UK centres and an opening site rate of 0.5 per month. The trial will be conducted in specialist pancreas cancer centres. Figure 1 summarises the trial design and Table 1 demonstrates the schedule of enrolment, interventions and assessments.

Participants

Adults (>18 years) with LAPC following first line chemotherapy with FOLFIRINOX will be eligible. Following FOLFIRINOX chemotherapy patients will be re-staged with CT abdomen, pelvis and chest (CAP) +/- an FDG Positron Emission Tomography (PET), depending on local site protocols. The imaging will be reviewed by the specialist multi-disciplinary team (sMDT) meeting.

For peer review only

Patients with borderline resectable disease will be excluded. The definition for this is summarised in Table 2. Other exclusion criteria include untreated biliary or gastric outlet obstruction, coagulopathy, chronic kidney disease >/= 3 (25), significant neurological disease, indwelling electrical devices, abdominal varices precluding safe access to the pancreas and significant co-morbidities that would contraindicate general anaesthesia and neuromuscular blockade. The full inclusion and exclusion criteria are summarised in Figure 2.

Figure 1: Trial flow diagram

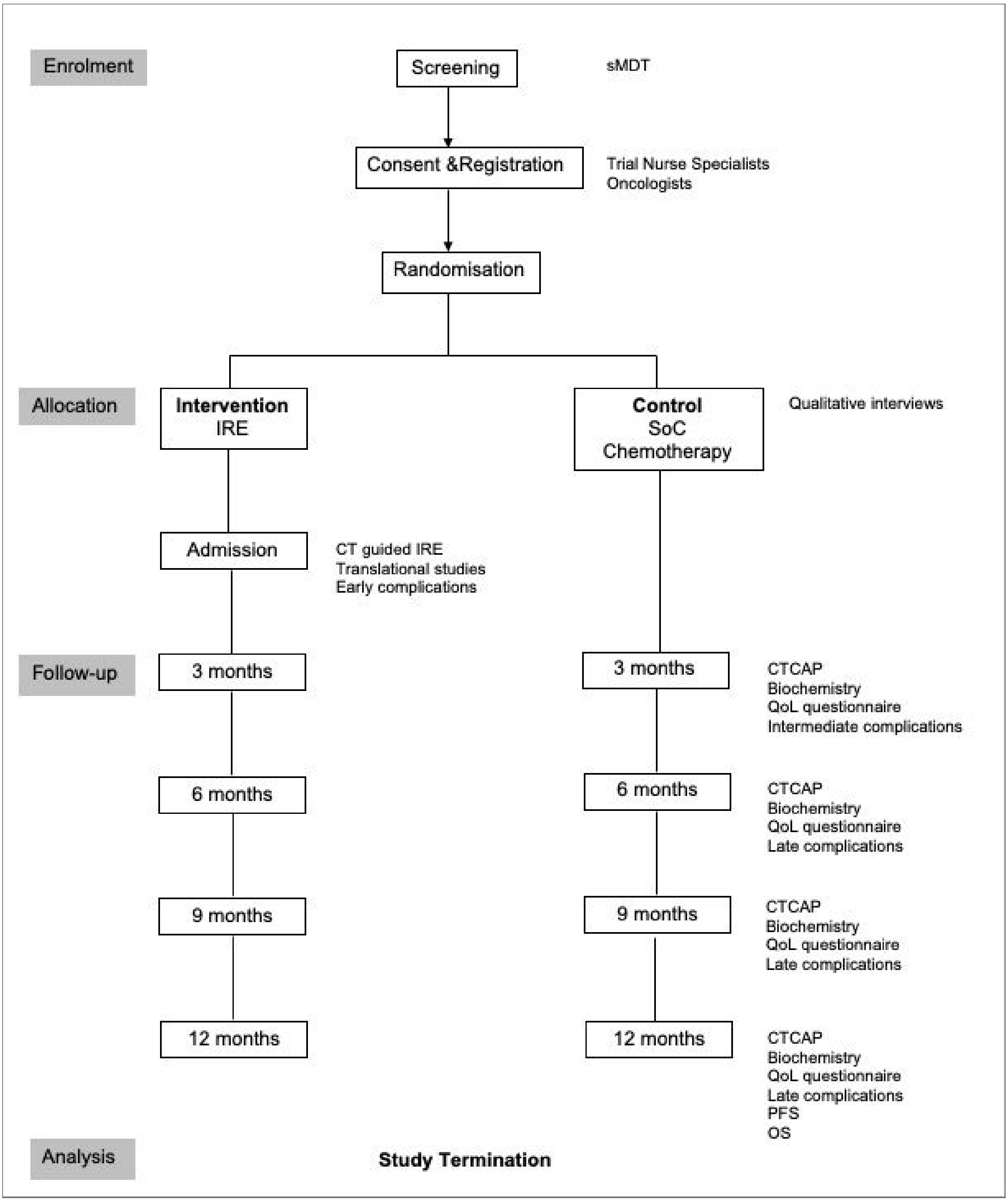

sMDT; specialist multidisciplinary team, IRE; irreversible electroporation, SoC; standard of care, CTCAP; computed tomography chest abdomen pelvis, QoL quality of life, PFS; progression free survival, OS; overall survival.

Figure 2: Inclusion and exclusion criteria of the LAP-PIE trial

Inclusion criteria
- Tissue confirmation
- Successful completion of FOLFIRINOX
- Cancer deemed inoperable at sMDT review
- Ability to provide informed consent
- > 18 years of age
- Considered amenable to IRE by pancreas IR
- WHO Performance status 0 /1.
- Maximum cancer diameter of 3.5 cm at time of IRE treatment.
- Medically fit for general anaesthetic

Exclusion Criteria
- First line chemotherapy other than FOLFIRINOX
- Concomitant malignancy (except skin and prostate cancers)
- Metastatic/Borderline resectable disease
- Untreated gastric outlet/biliary obstruction
- Co-morbidity precluding general anaesthesia +/- neuromuscular blockade
- Indwelling electrical devices
- CKD> 3
- History of neurological disease
- Abdominal varices
- Active bleeding
- Anticoagulation which cannot be discontinued,
- Coagulopathy (INR>1.5)/**any one** of the following:
  - Hb< 8 g/dL
  - absolute neutrophil count< 1500 cells/mL;
  - platelet count< 50,000.

FOLFIRINOX; 5-FluoroUracil, Leucovorin, Irinotecan and Oxaliplatin, sMDT; specialist multidisciplinary team, IRE; irreversible electroporation, IR; interventional radiologist, WHO; world health organisation, CKD; chronic kidney disease, Hb; haemoglobin

Table 1. Schedule of enrolment, interventions, and assessments of the LAP-PIE trial.

	Study Period									
	Enrolment	Allocation	Post-Allocation							Close-out
**TIMEPOINT**	*-t_1*	**0**	***Pre-IRE***	***6 hours post -IRE***	***6 days post -IRE***	***3 months***	***6 months***	***9 months***	***12 months***	
**ENROLMENT:**										
**Eligibility screen**	X									
**PIS given**	X									
Systemic treatment with FOLFIRINOX	X									
sMDT	X									
Informed consent	X									
**Allocation**		X								
**INTERVENTIONS:**										
IRE (intervention)			◆———	———	———	———	———	———	———◆	
Systemic chemotherapy (SoC)						◆———	———	———	———◆	
**ASSESSMENTS:**										
CT CAP	X					X	X	X	X	
Blood tests	X		X	X	X	X	X	X	X	X
HRQoL						X	X	X	X	X
Complications						X	X	X	X	X

PIS patient information sheet, sMDT specialist multidisciplinary team, IRE irreversible electroporation, SoC standard of care, CT CAP computed tomography chest abdomen pelvis, HRQoL health related quality of life.

For peer review only

Table 2: Definition of borderline resectable pancreatic cancer (15)

	Borderline Resectable	Unresectable
**Celiac artery**	Tumour without encasement or abutment	Tumour contact > 180º
**Superior Mesenteric artery**	Tumour abutment </= 180º	Tumour contact > 180º
**Superior Mesenteric vein (SMV)/Portal vein (PV)**	Tumour abutment of SMV/PV > 180º or abutting</= 180º with irregularity of the vein ± thrombosis with anatomical structures that still permit safe and complete resection with vein reconstruction	SMV/PV unreconstructible secondary to tumour involvement or occlusion
**Common hepatic artery**	Reconstructible short segment abutment	Unreconstructible interface between tumour mass and vessel

Adapted from Callery MP, Chang KJ, Fishman EK et al. Pre-treatment assessment of resectable and borderline resectable cancer: expert consensus statement. Ann Surg Oncol 2009; 16:1727-33.

Randomisation

Following confirmation of eligibility randomisation will be performed by the Liverpool Clinical Trials Centre (LCTC) using a web-based randomisation procedure. The LCTC will inform local centres of the treatment arm and the local LAP-PIE Clinical Trials Nurse will inform the patient and make arrangements for clinical review and the date for the chosen therapy.

Intervention

Pancreas IRE

Patients will be invited for an anaesthetic pre-assessment to ensure they are fit enough to undergo IRE under general anaesthesia prior to being booked for hospital admission. Patients will be admitted to a ward or radiology admissions unit under joint clinical care of a pancreas interventional radiologist and one of the other site leads (Surgery, Oncology).

The CT CAP performed on completion of FOLFIRINOX will be used by the interventional radiologist to plan IRE therapy. Under general anaesthesia IRE will be

delivered percutaneously using CT guidance. Timing of the procedure will be co-ordinated with the treating oncologist to ensure bone marrow recovery following FOLFIRINOX chemotherapy. A broad-spectrum antibiotic, based on individual centres first line prophylaxis for GI infection, will be given prior to the procedure, 6 hours and 12 hours following IRE.

The IRE procedure with be standardised to use between 2-6 IRE needles depending on the size, shape and relationship of critical structures to the tumour, with a maximum, inter-electrode distance of 2cm. A treatment margin of at least 0.5cm will be maintained. The needle placement prior to treatment will be recorded on CT.

IRE therapy will be delivered using ECG synchronisation to ensure the pulse is delivered during diastole. The length of the electrical pulse and amperage will be decided by the treating interventional radiologist and will be between 70-100 microseconds with an amperage of between 20-40 AMPs. The treating interventional radiologist will have relevant expertise in IRE procedures with a minimum of 5 IRE procedures on liver or pancreas performed previously.

Following IRE therapy full blood count, liver function tests, coagulation, renal function and amylase (or lipase) will monitored and patients will be admitted for a minimum of 24 hours to ensure adequate pain management and to monitor and manage any immediate complications. Participants will be discharged with pain relief as required and DVT prophylaxis for 4 weeks.

The intra-operative imaging will be reviewed by the lead radiologist at each site and if the entire tumour has been treated based on these images, the procedure will be deemed as successful. The technical success will be confirmed by central review. Contrast CT follow up scans will be assessed for evidence of tumour growth as indicated by mass enlargement or increased vascularity. In the absence of tumour

For peer review only

growth, the procedure will be considered successful at follow up. At the end of the follow up period, serial imaging will be analysed, and the tumour staged. Local disease control will be evaluated according to the Response Evaluation Criteria in Solid Tumours (RECIST). This will be performed by the radiology leads at each site.

Controls (Second line systemic chemotherapy)

Patients randomised to receive further chemotherapy will commence treatment within 6 weeks after sMDT or 4 weeks after randomisation, whichever is sooner. The second line chemotherapy regime will be selected at the discretion of the treating oncologist and will adhere to NICE guidelines and local protocols.

Outcomes

The primary objective of the LAP-PIE trial is to ascertain feasibility of such a trial concept. In particular, the ability to identify and recruit LAPC patients at the selected sites to the LAP-PIE trial will be evaluated. The primary outcome consists of recruitment rate, number of screening failures, number of patients completing study pathway as per protocol and trial withdrawal rate.

The secondary objectives of the LAP-PIE trial aim to assess the practicality and technical success of pancreas IRE treatment. Completion rates of IRE procedures, safety and complications of IRE treatment, technical success of intervention, acceptability of pancreas IRE therapy to patients and their clinicians, rate of withdrawal from the study and outcome measures including overall survival (measured from diagnosis and randomisation), progression free survival (measured as a binary outcome) and quality of life will be evaluated.

Follow up

All patients will be clinically reviewed every 3 months during which a CT CAP will be performed. Additional imaging can be performed if clinically indicated. Other

For peer review only

investigations will include biochemistry and haematology, CA19-9 levels, CRP, quality of life as measured by a validated questionnaire (EQ5D-5L) (26) and a clinical review of any adverse events experienced. This information will be recorded in an electronic case report form (eCRF).

Sample size calculation

Formal sample size calculations are not appropriate for a feasibility study. Sample sizes between 24 and 50 have been recommended to estimate the standard deviation required for a sample size calculation to allow for the design of a subsequent randomised controlled trial aimed at evaluating the cost effectiveness of IRE in patients with LAPC (27, 28). A convenient sample size of 50 patients, randomised equally to the two groups, has been selected.

Patient and Public Involvement (PPI)

PPI representatives have been involved in designing the trial ensuring that patient interests are central to the study. The design of the trial was presented to the Pancreas Cancer Action day which has a large representation from patients and carers and received extremely positive feedback. Two patient representatives will serve on the Trial management group (TMG) and will be involved in informing, monitoring and helping to interpret the findings from the study. Both patient representatives were involved in the trial design and in developing the trial protocol and patient information sheets. As the trial progresses they will help address any patient related concerns and will have input into the interpretation of the trial outcomes and how this will be communicated to the patient user groups. They will then be involved, along with other patient advocates, the trial management team and other key invited participants in the discussion of the trial findings at a focus group meeting and

For peer review only

the preparation of the protocol for a subsequent trial addressing efficacy and cost effectiveness of IRE therapy in pancreas cancer. Once the data analysis and interpretation has been completed they will contribute to the dissemination of the trial outcome information to patients support groups via social media.

Assessment of feasibility

One aspect of the feasibility evaluation of this trial is to evaluate the ability to recruit LAPC patients into a trial of this nature. The average number of patients with unresectable locally advanced pancreatic cancer within UK specialist pancreas units is 60 patients per centre per year (RFHL audit data 2017, unpublished). Staging following FOLFIRINOX chemotherapy will demonstrate localised but unresectable disease in approximately 40 out of 60 patients (65%) (19). These patients will be eligible for the LAP-PIE trial. We anticipate recruiting 20% of potential participants to the LAP-PIE trial which equates to 8 per centre per year. Recruitment within 5 centres would allow 40 UK patients per year. A recruitment time of 15 months would allow 50 patients to be recruited.

Assessment of Complications

Adverse Events (AE) for the Standard of Care Chemotherapy will be defined using the Common Terminology Criteria for Adverse Events (CTCAE) Version 4.0 (29). AEs for IRE will be defined using the Clavien Dindo classification of surgical complications (30). Complications greater than grade 3 on the CTCAE scale and greater than grade III on the Clavien Dindo scale will be reported.

A Serious Adverse Event (SAE) for patients randomised to receive Standard of Care Chemotherapy only, will be compared against the reference safety information (RSI) within the current summary of product characteristics (SmPC) for that drug. The RSI will be taken from the latest SmPC available for the drug on

For peer review only

https://www.medicines.org.uk/emc. The RSI for IRE will be from the Nanoknife system users' manual.

Qualitative Study in LAP-PIE trial

A qualitative study will be carried out to understand patients' and clinicians' perspectives and experiences of the procedure and the design of the trial. All patients approached to participate in the trial will be invited to participate. A purposive sample of healthcare professionals involved in the trial will also be invited. Trial nurses, local R&D (research and development) personnel and Network CRN (clinical research network) managers will be included. This information will be gathered via interviews by an experienced qualitative researcher.

Interviews will be transcribed verbatim and managed using computer-assisted qualitative data analysis software (iNVivo) (31). Emerging data will be used to modify trial information and protocol. This may include the development of training materials for staff and changes to participant information sheets.

Translational Study

A translational sub study will evaluate prognostic markers for outcome. Imaging prior to and during IRE therapy will be correlated with outcome as will the immune response to IRE therapy by assessing circulating immune responses at baseline, 6 hours and 6 days post therapy.

Progress to full trial

The criteria to progress to a subsequent full trial will be determined quantitatively as (A) the ability to recruit 50 patients to the study across the study sites

For peer review only

(B) a consent rate of 20+/-11%, (C) overall technical success rate of 50+/-14% and (D) a loss to follow-up of 10+/-8%.

Ethics

The trial will abide by the principles of the World Medical Association Declaration of Helsinki and has approval from an independent National Research Ethics Committee (REC).

Publication and Dissemination

Results from different centres will be analysed and published together following completion of the recruitment and follow up period. This will be presented to an international conference with an interest in hepato-pancreaticobiliary surgery, oncology and interventional radiology and published in a peer- reviewed journal.

Discussion

Trial justification

Local ablative therapy using IRE in patients with LAPC has been shown to be beneficial in prospective and retrospective studies (median survival 27 months) (13), however the increase in survival reported may be due to patient selection for IRE rather than the effect of IRE (selection bias). A systematic review of IRE therapy in LAPC included retrospective and prospective studies but no randomised controlled trials (RCT) (13). Moreover, the heterogeneity among individual trial participants including varying concomitant treatments prevented combination of data sets making it difficult to establish the true impact of IRE. Despite this, the systematic review is a

For peer review only

useful aid in summarising findings including complication rates following IRE and highlighting the current knowledge gaps such as the relationship between number of procedures performed and outcome and the impact of IRE on quality of life.

A study using propensity score matching (PSM) reported a median OS of LAPC patients undergoing IRE of 21 months (20), similar to that reported by the systematic review. Such a study may effectively adjust for observed variables between cohorts and can contribute to a more precise estimation of treatment response. However, selection bias cannot be completely eliminated because PSM does not adjust for unobserved differences between groups. In order to address selection bias, a well-designed randomised control trial is required.

Before IRE can be justified by NICE to be incorporated into routine clinical practice, a randomised control trial is needed. Whilst a larger trial is required to investigate IRE treatment in locally advanced pancreatic cancer, patients and clinicians may not be willing to enter the trial and therefore currently a large RCT is justified but not feasible.

Importance of doing feasibility

Clinical trials comparing different treatment types have high rates of failure (32). This is particularly true where there has been a lack of feasibility testing and qualitative evaluation of the study design (26,27). Furthermore, if a feasibility study demonstrates that a full trial would be unlikely to meet the required patient recruitment rate, intervention acceptability and other feasibility outcomes then the potential wasted cost of a larger clinical trial is avoided making feasibility trials an important first step (33). A feasibility trial will enable a realistic assessment of the capability of registered sites in patient enrolment and will compare IRE to the current standard of care. The qualitative study will explore the patient evaluation of information provided, the quality and clarity

For peer review only

of this information, the individual providing the information, the clinical equipoise, support and attitude of other clinicians and general practitioners and the views and experiences of patients' family and friends. The feasibility trial will provide information about challenges that may be encountered during enrolment and these factors can be mitigated for if a larger RCT is justified and subsequently planned.

Exclusion of borderline resectable disease

Patients with borderline resectable disease have been excluded from the LAP-PIE trial. The exact definition of borderline resectable pancreatic disease (BRPD) varies, but broadly it is characterised as tumour abutting or involving vascular structures (15). A number of studies have suggested that neoadjuvant chemotherapy or chemoradiotherapy may downsize the tumour allowing resection and preventing local recurrence (34-36). The LAP-PIE trial will exclude patients with BRPD because of the variability in radiological criteria used for BRPD and to avoid patients being recruited to the trial who would be considered in some pancreas units for surgical intervention.

Choice of FOLFIRINOX as first line

Previous studies have reported no significant improvement in survival when IRE is used as a first line treatment for unresectable LAPC (11, 12). Furthermore, systemic chemotherapy has previously been shown to downgrade a proportion of locally advanced unresectable cancer into resectable disease and therefore delivering IRE first line may result in an unnecessary, invasive procedure in a group of patients whose tumours may be downgraded and who would go on to have potentially curative resectional surgery.

FOLFIRINOX was specifically selected as the regime of choice because it has been shown to result in the greatest number of patients downgraded to resectable

For peer review only

disease (10). Therefore, the patients recruited into the LAP-PIE trial will be those with truly unresectable disease in whom the impact of IRE can be evaluated.

Translational studies

The translational study will determine the radiological and immune response of patients undergoing IRE and determine its biological significance with emphasis on overall survival and disease response. This data will provide valuable information about the type of patient that is likely to benefit the most from IRE and therefore form the basis of a management design that is tailor made to individual patients.

Mandated imaging in the LAP-PIE trial is a CT scan and this will provide information about contrast enhancement features. The images will be reviewed and information about tumour characteristics such as size, histology and grade will be collected. The relationship between treatment parameters and imaging findings during the IRE procedure, as well as pre-treatment PET scans, will be analysed. The data generated will be used to develop a mathematical model to predict IRE responses and help understand the significance of radiological changes and their biological significance. Blood samples will be taken from patients enrolled in the study and analysed for peripheral immune response markers. This will include; absolute lymphocyte count and eosinophils, neutrophil: leucocyte ratio, Tregs and IFNγ. Previous studies have demonstrated a detectable peripheral immune response in patients following IRE (37, 38). Analysing the immune response of patients undergoing IRE will provide information about whether a peripheral immune response can be defined in these patients and if this can predict treatment efficacy. This information may be used in the future to predict treatment response and guide future patient selection.

For peer review only

The qualitative sub-study will provide the opportunity to collate and analyse the experiences of both patients and clinicians in order to understand the perspectives and experiences of the procedure and trial providing useful information on the barriers and facilitators experienced by both groups.

Preliminary studies have shown that IRE in addition to chemotherapy may provide survival benefit compared to chemotherapy alone in LAPC patients. However, this may be due to patient selection for IRE. There are no randomised controlled trials addressing the issue. The LAP-PIE trial is the first UK-based RCT of pancreas IRE in LAPC patients. The trial will assess feasibility and safety of delivering this intervention and if successful, will progress to a subsequent large scale RCT trial in assessing the efficacy and cost-effectiveness of IRE therapy in pancreatic cancer. This research is required to investigate whether IRE improves survival, health-related quality of life, or both.

Although IRE is currently available, its mechanism of action and whether it has been optimised for treating different cancer types and stages remain areas that require further research. The LAP-PIE trial will provide evidence for supporting or refuting the role of IRE in patients with locally advanced pancreatic cancer.

For peer review only

References

1. Are C, Chowdhury S, Ahmad H, Ravipati A, Song T, Shrikandhe S, et al. Predictive global trends in the incidence and mortality of pancreatic cancer based on geographic location, socio-economic status, and demographic shift. J Surg Oncol. 2016;114(6):736-42.
2. Ghaneh P, Costello E, Neoptolemos JP. Biology and management of pancreatic cancer. Postgrad Med J. 2008;84(995):478-97.
3. Balaban EP, Mangu PB, Khorana AA, Shah MA, Mukherjee S, Crane CH, et al. Locally Advanced, Unresectable Pancreatic Cancer: American Society of Clinical Oncology Clinical Practice Guideline. J Clin Oncol. 2016;34(22):2654-68.
4. Ghosn M, Ibrahim T, Assi T, El Rassy E, Kourie HR, Kattan J. Dilemma of first line regimens in metastatic pancreatic adenocarcinoma. World J Gastroenterol. 2016;22(46):10124-30.
5. Rahib L, Fleshman JM, Matrisian LM, Berlin JD. Evaluation of Pancreatic Cancer Clinical Trials and Benchmarks for Clinically Meaningful Future Trials: A Systematic Review. JAMA Oncol. 2016;2(9):1209-16.
6. Wang C, Liu X, Wang X, Wang Y, Cha N. Effects of chemoradiotherapy and chemotherapy on survival of patients with locally advanced pancreatic cancer: A meta-analysis of randomized controlled trials. Medicine (Baltimore). 2018;97(36):e12260.
7. Excellence NIfHaC. Overview | Pancreatic cancer in adults: diagnosis and management | Guidance | NICE: NICE; 2020 [Available from: https://www.nice.org.uk/guidance/ng85.
8. Pavillard V, Formento P, Rostagno P, Formento JL, Fischel JL, Francoual M, et al. Combination of irinotecan (CPT11) and 5-fluorouracil with an analysis of cellular determinants of drug activity. Biochem Pharmacol. 1998;56(10):1315-22.

9. Ducreux M, Mitry E, Ould-Kaci M, Boige V, Seitz JF, Bugat R, et al. Randomized phase II study evaluating oxaliplatin alone, oxaliplatin combined with infusional 5-FU, and infusional 5-FU alone in advanced pancreatic carcinoma patients. Ann Oncol. 2004;15(3):467-73.
10. Suker M, Beumer BR, Sadot E, Marthey L, Faris JE, Mellon EA, et al. FOLFIRINOX for locally advanced pancreatic cancer: a systematic review and patient-level meta-analysis. Lancet Oncol. 2016;17(6):801-10.
11. Mansson C, Brahmstaedt R, Nygren P, Nilsson A, Urdzik J, Karlson BM. Percutaneous Irreversible Electroporation as First-line Treatment of Locally Advanced Pancreatic Cancer. Anticancer Res. 2019;39(5):2509-12.
12. Belfiore MP, Ronza FM, Romano F, Ianniello GP, De Lucia G, Gallo C, et al. Percutaneous CT-guided irreversible electroporation followed by chemotherapy as a novel neoadjuvant protocol in locally advanced pancreatic cancer: Our preliminary experience. Int J Surg. 2015;21 Suppl 1:S34-9.
13. Lafranceschina S, Brunetti O, Delvecchio A, Conticchio M, Ammendola M, Currò G, et al. Systematic Review of Irreversible Electroporation Role in Management of Locally Advanced Pancreatic Cancer. Cancers (Basel). 112019.
14. Hajatdoost L, Sedaghat K, Walker EJ, Thomas J, Kosari S. Chemotherapy in Pancreatic Cancer: A Systematic Review. Medicina (Kaunas). 542018.
15. Callery MP, Chang KJ, Fishman EK, Talamonti MS, William Traverso L, Linehan DC. Pretreatment assessment of resectable and borderline resectable pancreatic cancer: expert consensus statement. Ann Surg Oncol. 2009;16(7):1727-33.
16. Network NCC. Pancreatic Adenocarcinoma, Version 2.2017, NCCN Clinical Practice Guidelines in Oncology - NCCN-2017-pancreatic-cancer.pdf: NCCN; 2017 [Available from: http://clubedopancreas.pt/wp-content/uploads/2018/06/NCCN-2017-pancreatic-cancer.pdf.
17. Excellence NIfHaC. Recommendations | Pancreatic cancer in adults: diagnosis and management | Guidance | NICE: NICE; 2020 [Available from: https://www.nice.org.uk/guidance/ng85/chapter/Recommendations#managing-resectable-and-borderline-resectable-pancreatic-cancer.
18. Wills-Percy DDaS. St Luke's Cancer Alliance: Cancer treatment 2018 [Available from: http://stlukescanceralliance.co.uk/wp-content/uploads/2018/08/Gem-Cap-V3-8.18.pdf.
19. Hurt CN, Falk S, Crosby T, McDonald A, Ray R, Joseph G, et al. Long-term results and recurrence patterns from SCALOP: a phase II randomised trial of gemcitabine- or capecitabine-based chemoradiation for locally advanced pancreatic cancer. Br J Cancer. 2017;116(10):1264-70.
20. He C, Wang J, Sun S, Zhang Y, Lin X, Lao X, et al. Irreversible electroporation versus radiotherapy after induction chemotherapy on survival in patients with locally advanced pancreatic cancer: a propensity score analysis. BMC Cancer. 192019.
21. Narayanan G, Hosein PJ, Beulaygue IC, Froud T, Scheffer HJ, Venkat SR, et al. Percutaneous Image-Guided Irreversible Electroporation for the Treatment of Unresectable, Locally Advanced Pancreatic Adenocarcinoma. J Vasc Interv Radiol. 2017;28(3):342-8.
22. Kluger MD, Epelboym I, Schrope BA, Mahendraraj K, Hecht EM, Susman J, et al. Single-Institution Experience with Irreversible Electroporation for T4 Pancreatic Cancer: First 50 Patients. Ann Surg Oncol. 2016;23(5):1736-43.

For peer review only

23. Liu S, Qin Z, Xu J, Zeng J, Chen J, Niu L, et al. Irreversible electroporation combined with chemotherapy for unresectable pancreatic carcinoma: a prospective cohort study. Onco Targets Ther. 2019;12:1341-50.
24. Field W, Rostas JW, Martin RCG. Quality of life assessment for patients undergoing irreversible electroporation (IRE) for treatment of locally advanced pancreatic cancer (LAPC). Am J Surg. 2019;218(3):571-8.
25. Outcomes KDIG. KDIGO 2012 Clinical Practice Guidelines for Evaluation and Management of Chronic Kidney Disease 2013 [Available from: https://kdigo.org/wp-content/uploads/2017/02/KDIGO_2012_CKD_GL.pdf.
26. Group E. EQ-5D-5L 2009 [Available from: https://euroqol.org/eq-5d-instruments/eq-5d-5l-about/.
27. Julious SA. Sample size of 12 per group rule of thumb for a pilot study. Pharmaceutical Statistics. 2020;4(4):287-91.
28. Sim J, Lewis M. The size of a pilot study for a clinical trial should be calculated in relation to considerations of precision and efficiency. J Clin Epidemiol. 2012;65(3):301-8.
29. Common Terminology Criteria for Adverse Events (CTCAE) - CTCAE_4.03_2010-06-14_QuickReference_5x7.pdf 2020 [Available from: https://www.ncbi.nlm.nih.gov/pubmed/.
30. Dindo D, Demartines N, Clavien PA. Classification of surgical complications: a new proposal with evaluation in a cohort of 6336 patients and results of a survey. Ann Surg. 2004;240(2):205-13.
31. NVIVO. Qualitative Data Analysis Software | NVivo 2020 [Available from: https://www.qsrinternational.com/nvivo-qualitative-data-analysis-software/home.
32. Fogel DB. Factors associated with clinical trials that fail and opportunities for improving the likelihood of success: A review. Contemp Clin Trials Commun. 2018;11:156-64.
33. Morgan B, Hejdenberg J, Hinrichs-Krapels S, Armstrong D. Do feasibility studies contribute to, or avoid, waste in research? PLoS One. 2018;13(4):e0195951.
34. Pisters PW, Abbruzzese JL, Janjan NA, Cleary KR, Charnsangavej C, Goswitz MS, et al. Rapid-fractionation preoperative chemoradiation, pancreaticoduodenectomy, and intraoperative radiation therapy for resectable pancreatic adenocarcinoma. J Clin Oncol. 1998;16(12):3843-50.
35. Yeung RS, Weese JL, Hoffman JP, Solin LJ, Paul AR, Engstrom PF, et al. Neoadjuvant chemoradiation in pancreatic and duodenal carcinoma. A Phase II Study. Cancer. 1993;72(7):2124-33.
36. Evans DB, Rich TA, Byrd DR, Cleary KR, Connelly JH, Levin B, et al. Preoperative chemoradiation and pancreaticoduodenectomy for adenocarcinoma of the pancreas. Arch Surg. 1992;127(11):1335-9.
37. He C, Wang J, Sun S, Zhang Y, Li S. Immunomodulatory Effect after Irreversible Electroporation in Patients with Locally Advanced Pancreatic Cancer. J Oncol. 2019;2019:9346017.
38. Pandit H, Hong YK, Li Y, Rostas J, Pulliam Z, Li SP, et al. Evaluating the Regulatory Immunomodulation Effect of Irreversible Electroporation (IRE) in Pancreatic Adenocarcinoma. Ann Surg Oncol. 2019;26(3):800-6.

For peer review only

For peer review only

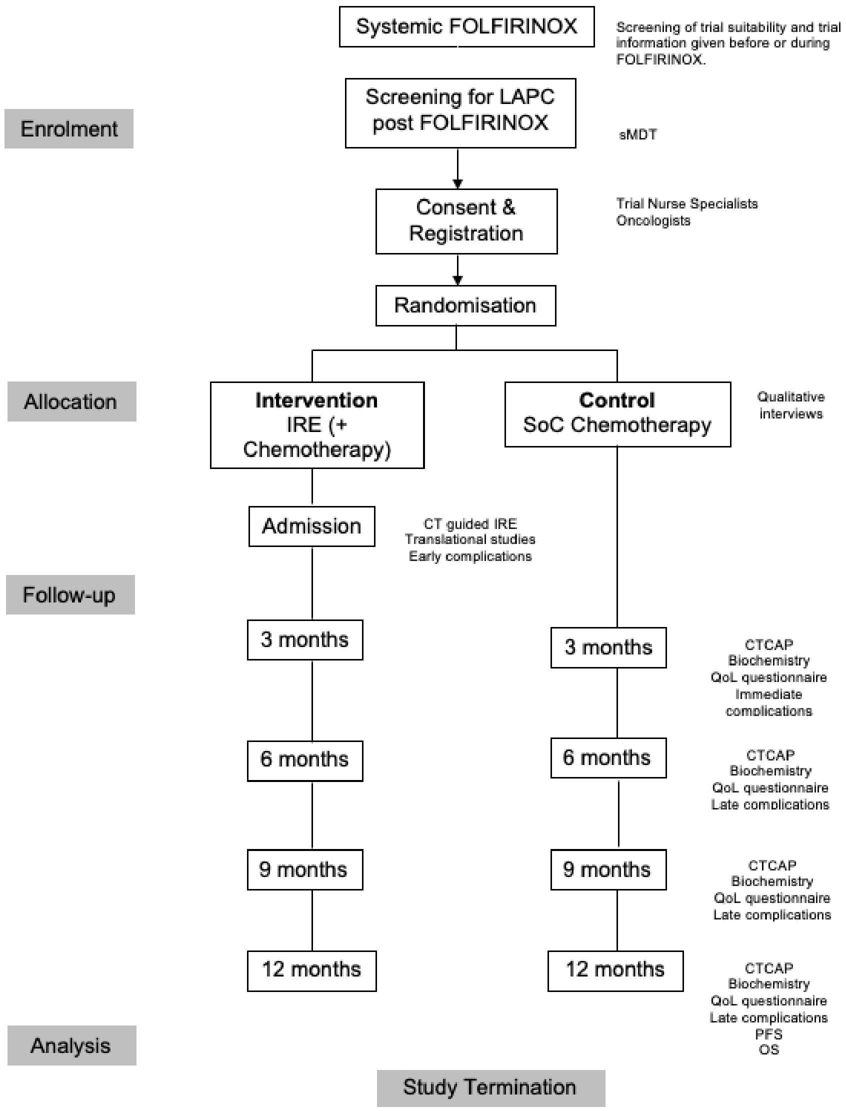

Trial flow diagram

230x256mm (72 x 72 DPI)

Inclusion criteria

- Tissue confirmation
- Successful completion of FOLFIRINOX
- Cancer deemed inoperable at sMDT review
- Ability to provide informed consent
- > 18 years of age
- Considered amenable to IRE by pancreas IR
- WHO Performance status 0 /1.
- Maximum cancer diameter of 3.5 cm at time of IRE treatment.
- Medically fit for general anaesthetic

Exclusion Criteria

- First line chemotherapy other than FOLFIRINOX
- Concomitant malignancy (except skin and prostate cancers)
- Metastatic/Borderline resectable disease
- Untreated gastric outlet/biliary obstruction
- Co-morbidity precluding general anaesthesia +/- neuromuscular blockade
- Indwelling electrical devices
- CKD> 3
- History of neurological disease
- Abdominal varices
- Active bleeding
- Anticoagulation which cannot be discontinued,
- Coagulopathy (INR>1.5)/**any one** of the following:
    - Hb< 8 g/dL
    - absolute neutrophil count< 1500 cells/mL;
    - platelet count< 50,000.

FOLFIRINOX; 5-FluoroUracil, Leucovorin, Irinotecan and Oxaliplatin, sMDT; specialist multidisciplinary team, IRE; irreversible electroporation, IR; interventional radiologist, WHO; world health organisation, CKD; chronic kidney disease, Hb; haemoglobin

Inclusion and exclusion criteria of the LAP-PIE trial

445x251mm (72 x 72 DPI)

Treatment of unresectable Locally Advanced Pancreatic cancer with Percutaneous Irreversible Electroporation (IRE) following initial systemic chemotherapy (LAP-PIE) trial: Study protocol for a feasibility randomised controlled trial.

Protocol

Version draft III- December 13, 2020

Study Principal Investigator:	Brian R. Davidson, MB ChB, MD, FRCS (Eng.), FRCPS (Glasg), Division of Surgery and Interventional Science, University College London
Sponsors:	Royal Free London NHS Foundation Trust
Protocol contributors:	Zainab Rai, (Division of Surgery and Interventional Science, University College London)<br>Veronica Ranieri, (Division of Surgery and Interventional Science, University College London)<br>Michael Stackpoole, (Liverpool Clinical Trials Unit, Liverpool)<br>Peter Littler, (Interventional Radiology, Freeman Hospital, Newcastle)<br>Dan Palmer, (Liverpool Clinical Trials Unit, Liverpool)<br>Paula Ghaneh, (Liverpool Clinical Trials Unit, Liverpool)<br>Kurinchi Gurusamy, (Division of Surgery and Interventional Science, University College London)<br>Derek Manas, (Newcastle NHS Foundation Trust, Newcastle)<br>Elena Pizzo, (Statistical sciences, University College London)<br>Eftychia E Psarelli, (University of Liverpool)<br>Brian Davidson, (Centre for Surgical Innovation, Organ Regeneration and Transplant University College London)

For peer review only - http://bmjopen.bmj.com/site/about/guidelines.xhtml

Trial identifiers:	Trial registered with International Standard Randomised Controlled Trial Number at https://www.isrctn.com/ISRCTN14986389<br>Trial ID: ISRCTN14986389<br>Date registered: 09/02/2021<br>PB-PG-1217-20042

For peer review only

For peer review only - http://bmjopen.bmj.com/site/about/guidelines.xhtml

REVISION HISTORY

Version I	Date 29/10/2020	Amendment Text	Description

For peer review only

For peer review only - http://bmjopen.bmj.com/site/about/guidelines.xhtml

Table of Contents

1. TRIAL SUMMARY
2. INTRODUCTION
2.1. Background and rationale
2.2. Objectives
2.3. Trial design
3. METHODS
3.1. Study setting
3.2. Eligibility criteria
3.2.1. Inclusion criteria
3.2.2. Exclusion criteria
3.3. Interventions
3.3.1. Intervention description
3.3.2. Modifications
3.3.3. Adherence
3.3.4. Concomitant care
3.4. Outcomes
3.5. Participant timeline
3.6. Sample size
3.7. Recruitment
3.8. Allocation
3.9. Blinding (masking)
3.9.1. Blinding mechanism
3.9.2. Emergency unblinding
3.10. Data collection
3.10.1. Trial procedures and evaluations
3.10.2. Retention
3.11. Data management
3.12. Statistical methods
3.12.1. Outcomes
3.12.2. Additional analyses
3.12.3. Analysis population and missing data
3.13. Data monitoring
3.13.1. Formal committee
3.13.2. Interim analysis
3.14. Safety/harms
3.15. Auditing
4. ETHICS AND DISSEMINATION
4.1. Research ethics approval
4.2. Protocol amendments
4.3. Informed consent process
4.4. Confidentiality
4.5. Declaration of interests
4.6. Access to data
4.7. Dissemination policy

For peer review only - http://bmjopen.bmj.com/site/about/guidelines.xhtml

4.7.1. Trial results ....
4.7.2. Authorship ....
4.7.3. Reproducible research ....
5. STUDY ADMINISTRATION ....
5.1. Key contacts ....
5.2. Funders ....
5.3. Roles and responsibilities ....
5.3.1. Protocol contributors ....
5.3.2. Sponsor and funder ....
5.3.3. Trial committees ....
6. REFERENCES ....
7. APPENDICES ....
7.1. Informed consent materials ....

For peer review only

For peer review only - http://bmjopen.bmj.com/site/about/guidelines.xhtml

1. TRIAL SUMMARY

World Health Organization Registration Data Set

Title	Treatment of unresectable Locally Advanced Pancreatic cancer with Percutaneous Irreversible Electroporation (IRE) following initial systemic chemotherapy (LAP-PIE) trial: Study protocol for a feasibility randomised controlled trial.
Primary registry and trial identifying number	ISRCTN.org<br>pending
Secondary identifying numbers	PB-PG-1217-20042
Sources of monetary or material support	Sources and types of financial, material, and other support<br>**Funding body:**<br>National Institute for Health Research (NIHR), Research for Patient Benefit (RfPB) programme<br>Central Commissioning Facility<br>Grange House<br>15 Church Street<br>Twickenham<br>TW1 3NL<br>**Training support:**<br>Angiodynamics. Manufacturer of CE marked device that is in use in clinics already. Angiodynamics will provide training and support as normal during this study and supply of the needle probes used through the duration.<br>**Other Support.**<br>Operational Director Liverpool Clinical Trials Centre Block C, Waterhouse Building1-3 Brownlow StreetLiverpoolL69 3GLTel: 0151 794 8897Fax: 0151 794 8931LAP-PIE@liverpool.ac.uk
Primary sponsor	Royal Free London NHS Foundation Trust
Secondary sponsors (if any)	Not applicable
Central contact	Operational Director<br>Liverpool Clinical Trials Centre Block C, Waterhouse Building 1-3 Brownlow Street Liverpool L69 3GL<br>0151 794 8897<br>LAP-PIE@liverpool.ac.uk

For peer review only - http://bmjopen.bmj.com/site/about/guidelines.xhtml

Study officials/Investigators	Brian R. Davidson, MB ChB, MD, FRCS (Eng), FRCPS (Glasg)<br>Division of Surgery and Interventional Science, University College London<br>Royal Free Hospital, 9th Floor (East), 2QG, 10 Pond St, London NW3 2PS<br>020 7794 0500, Ext.33603<br>b.davidson@ucl.ac.uk<br>Study Principal Investigator
Brief title	Treatment of unresectable Locally Advanced Pancreatic cancer with Percutaneous Irreversible Electroporation (IRE) following initial systemic chemotherapy: a feasibility randomised controlled trial
Acronym	LAP-PIE
Countries of recruitment	United Kingdom
Condition(s) or focus of study	Locally advanced pancreatic cancer, Irreversible electroporation, Feasibility trial
Interventions	
IRE (interventional)	Intervention type: Device<br>Intervention name: Irreversible Electroporation<br>Other names: IRE<br>Intervention description: The length of the electrical pulse and amperage will be decided by the treating interventional radiologist and will be between 70-100 microseconds with an amperage of between 20-40 AMPs. The treating interventional radiologist will have relevant expertise in IRE procedures with a minimum of 5 IRE procedures on liver or pancreas performed previously.
Standard of Care (control)	Intervention type: Drug<br>Intervention name: 5-FluoroUracil, Leucovorin, Irinotecan and Oxaliplatin<br>Other names: FOLFIRINOX<br>Intervention description: Patients randomised to receive further chemotherapy will commence treatment within 6 weeks after sMDT or 4 weeks after randomisation, whichever is sooner. The second line chemotherapy regime will be selected at the discretion of the treating

For peer review only - http://bmjopen.bmj.com/site/about/guidelines.xhtml

	oncologist and will adhere to NICE guidelines and local protocols.
Key eligibility criteria	Age eligibility: 18 years or older<br>Sex eligibility: Both<br>Accepts healthy volunteers: No<br><br>Inclusion criteria:<br>1. Tissue confirmation<br>2. Successful completion of FOLFIRINOX<br>3. Cancer deemed inoperable at sMDT review,<br>4. WHO Performance status 0 /1<br>5. Maximum cancer diameter of 3.5 cm at time of IRE treatment<br><br>Exclusion criteria:<br>1. First line chemotherapy other than FOLFIRINOX<br>2. Concomitant malignancy (except skin and prostate cancers)<br>3. Metastatic/Borderline resectable disease<br>4. Untreated gastric outlet/biliary obstruction<br>5. Co-morbidity precluding general anaesthesia +/- neuromuscular blockade<br>6. Indwelling electrical devices<br>7. Chronic Kidney Disease > 3<br>8. History of neurological disease<br>9. Abdominal varices<br>10. Active bleeding<br>11. Anticoagulation which cannot be discontinued<br>12. Coagulopathy
Study design	Study type: Interventional trial<br>Allocation: Randomized<br>Intervention model: Parallel group<br>Primary purpose: Other<br>Phase: Feasibility
Masking	None
Date of enrolment	
Target sample size	50
Recruitment status	Active, not recruiting

For peer review only - http://bmjopen.bmj.com/site/about/guidelines.xhtml

Primary outcomes	To ascertain the feasibility of identifying and recruiting LAPC patients at the selected sites to the LAP-PIE trial. The primary outcome consists of recruitment rate, number of screening failures, number of patients completing study pathway as per protocol and trial withdrawal rate.<br>Timeframe: 36 months
Secondary outcomes	To assess the practicality and technical success of pancreas IRE treatment. Completion rates of IRE procedures, safety and complications of IRE treatment, technical success of intervention, acceptability of pancreas IRE therapy to patients and their clinicians, rate of withdrawal from the study and outcome measures including overall survival (measured from diagnosis and randomisation), progression free survival (measured as a binary outcome) and quality of life will be evaluated.<br>Timeframe: 36 months

For peer review only

For peer review only - http://bmjopen.bmj.com/site/about/guidelines.xhtml

2. INTRODUCTION

2.1. Background and rationale

Pancreas cancer and LAPC

Pancreatic cancer (PC) is one of the deadliest cancers worldwide. It has a five-year survival rate of less than 6% and an increasing incidence (1). The low survival rate is mainly due to late diagnosis, as PC often remains asymptomatic until it becomes advanced. Surgery (pancreatic resection) is the only potentially curative treatment. However, only 10 to 20% of patients are eligible for surgery (2). Approximately 30% of all patients have locally advanced pancreatic cancer (LAPC) at diagnosis and are not considered for surgical resection (3).

Chemotherapy for LAPC

Systemic chemotherapy is the standard treatment for unresectable pancreatic cancer (4). Despite advances in drug therapy for pancreas cancer over the last 20 years the overall survival (OS) and progression free survival (PFS) of these patients has not significantly improved (5). Furthermore, a meta-analysis reported that chemoradiation is not superior to chemotherapy alone in patients with LAPC, with comparable overall survival and higher rates of grade 3–4 adverse events (6).

In the UK, NICE guidelines, published in 2018 recommend systemic combination chemotherapy for both locally unresectable and metastatic pancreatic cancer (7). NICE recommends first-line combination chemotherapy with 5-FluoroUracil, Leucovorin, Irinotecan and Oxaliplatin (FOLFIRINOX) in fit patients (Eastern Cooperative Oncology Group (ECOG) performance status of 0-1). In patients with co-morbidities and restricted activities, or those unable to tolerate this regime, NICE recommends gemcitabine alone (6). Other NICE recommended combinations include Nab-paclitaxel and gemcitabine combined with capecitabine (GemCap) (7). Although FOLFIRINOX shows the best objective response in pancreatic cancer (8, 9) it is associated with more frequent and severe side effects, including neutropenia, diarrhoea and peripheral neuropathy (10).

First Line Chemotherapy

The timing of chemotherapy in relation to IRE therapy may influence outcome. Studies where IRE has been delivered before chemotherapy have reported only modest increases in median OS (11, 12) whereas studies evaluating the impact of IRE after induction treatment report a survival benefit of up to 27 months (13). This may be because chemotherapy responders who have better outcomes are being selected to undergo IRE or alternatively it may suggest that chemotherapy modifies the tumour microenvironment making it more sensitive to IRE.

Repeat staging after first line chemotherapy and possibility of resection

Staging is repeated following first line chemotherapy for LAPC and imaging is reviewed once again regarding suitability for resection. In patients with locally advanced disease undergoing first line treatment with FOLFIRINOX, 25.9% underwent resection, and in this cohort, 78.4% had R0 resection, however the long-term outcomes for these patients is not reported in the studies, and there is still a possibility of recurrence albeit at later date (14).

Locally advanced and borderline unresectable LAPC

The definition of borderline resectable disease is controversial (15), whereas the definition of unresectable locally advanced disease has clear consensus (16).

For peer review only - http://bmjopen.bmj.com/site/about/guidelines.xhtml

Therefore, the LAP-PIE trial will only involve patients with locally advanced disease fulfilling published criteria.

Second line chemotherapy for LAPC

NICE recommends that patients who have previously undergone FOLFIRINOX therapy as first line should be offered Gemcitabine based second-line chemotherapy (17). Other agents used in second line treatments include Capecitabine and combination therapy with Gemcitabine and Capecitabine (18). If new agents for second line therapy are approved by NICE prior to or during the LAP-PIE trial these can be offered in the second line chemotherapy arm and following IRE therapy if considered appropriate by the treating oncologist.

IRE for LAPC

Irreversible electroporation is a novel ablative method for treating solid cancers (19). IRE treatment destroys cancer cells using electricity. It can be delivered via an open approach involving a laparotomy and placement of probes around the cancer or via a percutaneous approach which requires image guidance, via CT or ultrasound guidance. Both methods require the patient to be under general anaesthesia with neuromuscular blockade. A single study using propensity score matching reported a median OS of 21.6 months in patients with LAPC treated with induction chemotherapy followed by IRE (20). A systematic review on pancreas IRE therapy from 2019 reported an OS from diagnosis or treatment of up to 27 months and a morbidity rate of 30% (13).

Complications of pancreas IRE therapy

Reported complications following pancreas IRE include pancreatic fistula, venous and arterial thrombosis, arterial pseudoaneurysm and pancreatic abscess (13). The average morbidity rate in the studies examined is 34% but can reach up to 62% (21) The average rate of severe complications (defined as greater than Grade III on the Clavien-Dindo scale) following IRE is 8% but can be as high as 44% (22). The average procedure related mortality rate in these studies is 3% (12).

There are significant variations in the percentage and severity of complications reported. This difference may be due to the case selection, whether IRE is carried out during surgery or electively in the IR suite, the treatment algorithm, the completeness of reporting from treatment centres or the experience of the radiologist and/or centre. Only two studies specifically mention the experience of clinicians delivering IRE (12, 23) and currently, there is no data on the number of IRE procedures performed and the relationship with outcomes.

IRE is a palliative option and therefore its impact on patient's quality of life (QoL) is an essential consideration. A single-centre prospective study evaluating the QoL pre and post IRE in patients with LAPC demonstrated that IRE had no adverse effect on QoL however, concomitant treatment with chemotherapy after IRE was not taken into account and may confound the results (24). Currently there are no recognised QoL scores that compare IRE with no treatment or systemic chemotherapy. The data regarding the improvement in QoL in LAPC undergoing IRE is weak and needs further exploration.

Aim of the treatment of unresectable Locally Advanced Pancreatic cancer with Percutaneous Irreversible Electroporation (IRE) following initial systemic chemotherapy (LAP-PIE) trial

For peer review only - http://bmjopen.bmj.com/site/about/guidelines.xhtml

The current information available in the literature regarding the use of IRE in the treatment of LAPC is from retrospective and prospective cohort studies. A prospective randomised controlled trial is required to demonstrate cost effectiveness of pancreas IRE therapy. The LAP-PIE trial is a feasibility trial to determine whether patients would be willing to have pancreas IRE treatment, whether they would accept randomisation to IRE or standard of care chemotherapy, whether they would comply with treatment and study data collection and to collect preliminary data on efficacy end points and costs with which to design a subsequent trial on cost effectiveness.

2.2. Objectives

Primary Objective:
To ascertain the feasibility of performing a Randomised Controlled Study on Irreversible Electroporation following initial systemic chemotherapy for Locally Advanced Pancreatic Cancer.

Secondary Objectives:
To Assess:

1. Recruitment to a trial of this nature
2. The practicality and technical success of pancreas IRE treatment
3. The acceptability of pancreas IRE therapy to patients and their clinicians
4. Rate of withdrawal from the study
5. Safety of the IRE treatment
6. Relative importance of different outcome measures (Survival and Quality of Life)

2.3. Trial design

Trial Design
The LAP-PIE trial is a UK multi-centre, feasibility study that will recruit 50 patients with LAPC in whom the disease has remained localised and unresectable following FOLFIRINOX chemotherapy. All participants will be offered further chemotherapy according to standard centre protocols and half will also be offered IRE. It is an open-label study.

3. METHODS

3.1. Study setting

<u>Study setting</u>
The trial will be conducted in Hepato-Pancreato-Biliary (HPB) Centres within the UK. Participating centres will be identified and selected for their expertise to carry out IRE for locally advanced pancreatic cancer (LAPC). Each centre must have the required radiological expertise with pancreas IRE therapy and in addition oncology and surgery support to undertake the trial.

For peer review only - http://bmjopen.bmj.com/site/about/guidelines.xhtml

The IRE manufacturer, Angiodynamics, will ensure that involved centres have appropriate equipment, technical support and the interventional radiologists have adequate advice and mentoring particularly for the initial phase of the trial.

Each centre will complete a feasibility questionnaire to ensure that the centre has the appropriate facilities, qualified personnel and capacity to set up and run the trial in accordance with the current approved protocol and GCP standards.

A list of study sites can be obtained from contacting the central enquiry for the LAP-PIE trial at LAP-PIE@liverpool.ac.uk

3.2. Eligibility criteria

3.2.1. Inclusion criteria

1. Tissue confirmation
   Histopathological confirmation of pancreatic adenocarcinoma
2. Successful completion of FOLFIRINOX
3. Cancer deemed inoperable at sMDT review,
   sMDT (specialist multi-disciplinary team)
4. WHO Performance status 0 /1
   World Health Organisation (WHO)
5. Maximum cancer diameter of 3.5 cm at time of IRE treatment
   Retrospective and prospective studies suggest minimal clinical benefit of treating tumours > 3.5cm

3.2.2. Exclusion criteria

1. • First line chemotherapy other than FOLFIRINOX
2. • Concomitant malignancy (except skin and prostate cancers)
3. • Metastatic/Borderline resectable disease
4. • Untreated gastric outlet/biliary obstruction
5. • Co-morbidity precluding general anaesthesia +/- neuromuscular blockade
6. Indwelling electrical devices
7. Chronic Kidney Disease > 3
8. History of neurological disease
9. Abdominal varices
10. Active bleeding
11. Anticoagulation which cannot be discontinued
12. Coagulopathy
    (INR>1.5)/any one of the following, Hb< 8 g/dL, absolute neutrophil count< 1500 cells/mL, platelet count< 50,000.

3.3. Interventions

For peer review only - http://bmjopen.bmj.com/site/about/guidelines.xhtml

3.3.1. Intervention description

Intervention

Pancreas IRE

Patients will be invited for an anaesthetic pre-assessment to ensure they are fit enough to undergo IRE under general anaesthesia prior to being booked for hospital admission. Patients will be admitted to a ward or radiology admissions unit under joint clinical care of a pancreas interventional radiologist and one of the other site leads (Surgery, Oncology).

The CT CAP performed on completion of FOLFIRINOX will be used by the interventional radiologist to plan IRE therapy. Under general anaesthesia IRE will be delivered percutaneously using CT guidance. Timing of the procedure will be co-ordinated with the treating oncologist to ensure bone marrow recovery following FOLFIRINOX chemotherapy. A broad-spectrum antibiotic, based on individual centres first line prophylaxis for GI infection, will be given prior to the procedure, 6 hours and 12 hours following IRE.

The IRE procedure with be standardised to use between 2-6 IRE needles depending on the size, shape and relationship of critical structures to the tumour, with a maximum, inter-electrode distance of 2cm. A treatment margin of at least 0.5cm will be maintained. The needle placement prior to treatment will be recorded on CT.

IRE therapy will be delivered using ECG synchronisation to ensure the pulse is delivered during diastole. The length of the electrical pulse and amperage will be decided by the treating interventional radiologist and will be between 70-100 microseconds with an amperage of between 20-40 AMPs. The treating interventional radiologist will have relevant expertise in IRE procedures with a minimum of 5 IRE procedures on liver or pancreas performed previously.

Following IRE therapy full blood count, liver function tests, coagulation, renal function and amylase (or lipase) will monitored and patients will be admitted for a minimum of 24 hours to ensure adequate pain management and to monitor and manage any immediate complications. Participants will be discharged with pain relief as required and DVT prophylaxis for 4 weeks.

The intra-operative imaging will be reviewed by the lead radiologist at each site and if the entire tumour has been treated based on these images, the procedure will be deemed as successful. The technical success will be confirmed by central review. Contrast CT follow up scans will be assessed for evidence of tumour growth as indicated by mass enlargement or increased vascularity. In the absence of tumour growth, the procedure will be considered successful at follow up. At the end of the follow up period, serial imaging will be analysed, and the tumour staged. Local disease control will be evaluated according to the Response Evaluation Criteria in Solid Tumours (RECIST). This will be performed by the radiology leads at each site.

Controls (Second line systemic chemotherapy)

Patients randomised to receive further chemotherapy will commence treatment within 6 weeks after sMDT or 4 weeks after randomisation, whichever is sooner. The second line chemotherapy regime will be selected at the discretion of the treating oncologist and will adhere to NICE guidelines and local protocols.

For peer review only - http://bmjopen.bmj.com/site/about/guidelines.xhtml

3.3.2. Modifications

Premature Discontinuation of treatment

Participants may discontinue IRE or standard of care chemotherapy for reasons including, but not limited to:

- Participant-led i.e. request by the participant / person with parental responsibility / legal representative / consultee
- Unacceptable toxicity/side effects
- Intercurrent illness preventing further treatment.
- Pregnancy
- Death
- Clinician-led:
  - Any change in the participant's condition that justifies the discontinuation of treatment in the clinician's opinion.
  - Reasons of non-adherence or non-compliance with treatment or other trial procedures
  - Participant meets an exclusion criterion (either newly developed or not previously recognised)

3.3.3. Adherence

All sites will have a nominated clinical lead in each respective speciality (Surgery, Interventional Radiology and Oncology) who will be responsible for adhering to intervention protocol. They will be invited to attend Investigator meetings and be kept abreast of any changes to the protocol/progress of the trial.

Both the interventional arm (IRE) and the Standard of Care (Chemotherapy) requires patients to attend hospital and therefore no specific adherence strategies have been developed. Participants will be encouraged to attend these appointments.

3.3.4. Concomitant care

Concomitant Medications/Treatments

Concomitant treatment for conditions other than Locally Advanced Pancreatic Cancer may be continued/started throughout the trial in their standard dose as long as it does not affect inclusion or exclusion criteria. Use of concomitant treatment must be recorded in the patient medical record and study CRFs: the name of the drug, dose, start/stop dates and the indication for it are necessary.

Use of any other non-marketed or investigational products during the trial are NOT permitted.

Supportive medications such as analgesics, anti-emetics etc. should be given per local practice. Analgesic use prior to commencing second line therapy should be documented in the Case Report Form (CRF) with drugs, dose and frequency of use.

For peer review only - http://bmjopen.bmj.com/site/about/guidelines.xhtml

For peer review only

Medications Permitted

All medication necessary for the wellbeing of the patient, which is not expected to interfere with the evaluation of the IRE therapy and aftercare may be administered. The local investigator may contact the study team for guidance from the Chief Investigator or delegated medical expert. Particular attention should be given to treatment related to the side effects and risks of the IRE therapy.

IRE will be carried out under general anaesthetic and neuromuscular relaxation. Broad spectrum antibiotics will be administered pre and for 2 doses post procedure.

Medications Not Permitted/ Precautions Required

There are no medications which would contra-indicate pancreas IRE therapy.
As with all interventions patients with abnormal blood clotting or taking anticoagulant medication should be considered with caution and withdrawal of anticoagulant medication or alternative short-lived anticoagulant administered until the patient recovers from the procedure at which time routine medication will be re-commenced.

Data on Concomitant Medication

The systemic chemotherapy (drug/dose/duration) will be recorded for both treatment groups over the 12 month follow up period. Concomitant medication will be collected at baseline and updated through the study duration. Each visit CRF will prompt the local research team to complete the concomitant medication form for any new drugs and to ensure the start and stop date of previous medication is stated.

3.4. Outcomes

Outcomes

The primary objective of the LAP-PIE trial is to ascertain feasibility of such a trial concept. In particular, the ability to identify and recruit LAPC patients at the selected sites to the LAP-PIE trial will be evaluated. The primary outcome consists of recruitment rate, number of screening failures, number of patients completing study pathway as per protocol and trial withdrawal rate.

The secondary objectives of the LAP-PIE trial aim to assess the practicality and technical success of pancreas IRE treatment. Completion rates of IRE procedures, safety and complications of IRE treatment, technical success of intervention, acceptability of pancreas IRE therapy to patients and their clinicians, rate of withdrawal from the study and outcome measures including overall survival (measured from diagnosis and randomisation), progression free survival (measured as a binary outcome) and quality of life will be evaluated.

3.5. Participant timeline

For peer review only - http://bmjopen.bmj.com/site/about/guidelines.xhtml

Schedule of enrolment, interventions, and assessments of the LAP-PIE trial.

	**Study Period**									
	**Enrolment**	**Allocation**	**Post-Allocation**							**Close-out**
**TIMEPOINT**	***-t$_1$***	**0**	***Pre-IRE***	***6 hours post -IRE***	***6 days post -IRE***	***3 months***	***6 months***	***9 months***	***12 months***	
**ENROLMENT:**										
**Eligibility screen**	X									
**PIS given**	X									
Systemic treatment with FOLFIRINOX	X									
sMDT	X									
Informed consent	X									
**Allocation**		X								
**INTERVENTIONS:**										
IRE (intervention)										
Systemic chemotherapy (SoC)										
**ASSESSMENTS:**										
CT CAP	X					X	X	X	X	
Blood tests	X		X	X	X	X	X	X	X	X
HRQoL						X	X	X	X	X
Complications						X	X	X	X	X

PIS; Patient Information Sheet, FOLFIRINOX; 5-FluoroUracil, Leucovorin, Irinotecan and Oxaliplatin, sMDT; specialist Multi-Disciplinary Team, HRQoL; Health Related Quality of Life

For peer review only

For peer review only - http://bmjopen.bmj.com/site/about/guidelines.xhtml

3.6. Sample size

A convenient sample size of 50 patients, randomised equally to the two groups, has been chosen for this feasibility study. Formal sample size calculations are not appropriate for a feasibility study. However, this size will allow us to assess whether the consent rate of 20% has been achieved with a 95% confidence interval of +/- 11%. This sample size will also allow us to assess whether the technical success of IRE is 50% with a 95% confidence interval of +/- 14%, and loss to follow-up is 10% with a 95% confidence interval of +/-8%. In addition, sample sizes between 24 and 50 have been recommended to estimate the standard deviation required for a sample size calculation to allow us to design a large randomised controlled trial aimed at evaluating the cost effectiveness of IRE following chemotherapy for locally advanced pancreatic cancer (24, 25).

The recruitment target is 50 patients in 15 months from 7 UK centres, assuming a recruitment rate of 0.8 patients per site per month

3.7. Recruitment

This is a feasibility trial and one key aspect of feasibility evaluation is the ability to recruit patients with unresectable locally advanced pancreatic cancer into a trial of this nature. We will explore the reasons for participation and non-participation of patients and patients' and clinicians' acceptability of the trial to assist in optimisation of recruitment strategies employed for the subsequent trial. Non-participation can be related to how the clinical trial is presented to the patient, and how the patient assimilates this information. It is therefore important to understand how patients perceive information about potential participation and their experiences of receiving information on the trial.

3.8. Allocation

Allocation Sequence Generation

Participants will be randomised on a 1:1 basis. Randomisation lists shall be produced by the trial statistician using randomly permuted blocks Lists created using Stata (Version 14 or higher) using the add-on package 'ralloc'

Patient allocations will be irrevocably generated upon completion of the web-based randomisation form by a delegated member of the trial research team. Allocation concealment will be ensured as the service will not release the randomisation code until the patient has been recruited into the trial; this takes place after all baseline measurements have been completed.

For peer review only - http://bmjopen.bmj.com/site/about/guidelines.xhtml

3.9. Blinding (masking)

3.9.1. Blinding mechanism

The intervention is a procedure requiring expertise and general anaesthesia therefore it is not possible or appropriate to design this study as a blinded trial

3.9.2. Emergency unblinding

Not a blinded study therefore not applicable

3.10. Data collection

3.10.1. Trial procedures and evaluations

Data Collection Methods

Each participating site will maintain appropriate medical and research records for this trial, in compliance with ICH E6 GCP, Section 4.9 and regulatory and institutional requirements for the protection of confidentiality of subjects.

Data that is to be recorded directly on the electronic Clinical Record Forms (eCRF) or workbooks (i.e. no prior electronic or written record of the data), is to be considered to be source data e.g. questionnaires

Date(s) of informed consent processes (including date of provision of patient information, randomisation number and the fact that the patient is participating in a clinical trial (including possible treatment arms) will be added to the patient's medical record chronologically.

Data will be entered into MACRO and PHAROS by members of the research team at site. Training will be provided prior to any data entry. The eCRF workbooks that replicate each MACRO and PHAROS form must be retained at site for the study.

Questionnaires are a source document and sites will retain a copy at site in the Investigator Site File (ISF) (part of the Trial Master File).

Quality Assurance and Control

Quality assurance will be maintained to the required level via electronic database validations, internal LCTC quality processes and procedures, training of those involved in trial procedures and regular monitoring and audit.

The TMG will be involved in monitoring screening, randomisation and consent rates, as well as compliance with processes detailed in the protocol. Any activity that is determined to be a breach of the study protocol will be recorded on a protocol deviation form and reviewed by the study CI to confirm severity, the impact on the study and whether any corrective and preventative action is required.

The LAP-PIE study will have a designated data management and query resolution plan in place and specific instructions on how to complete the eCRF forms and workbooks. A

For peer review only - http://bmjopen.bmj.com/site/about/guidelines.xhtml

member of the study team will periodically review the study for missing or erroneous/anomalous data based on validations built into the database.

Study Instruments

The EQ5D-5L health questionnaire will be used at Baseline and each follow up visit in order to assess quality of life for all patients on the study. The EQ5D-5L is descriptive system for health-related quality of life states in adults, consisting of five dimensions (Mobility, Self-care, Usual activities, Pain & discomfort, Anxiety & depression), each of which has five severity levels that are described by statements appropriate to that dimension. It has been used to assess quality of life in a number of conditions (ref) and is considered a reliable and valid health related questionnaire (ref).

Data collection forms can be requested by contacting the central LAP-PIE trial team on LAP-PIE@liverpool.ac.uk

3.10.2. Retention

Plans to Promote Participant Retention and Complete Follow-up.

Specific plans to promote participant retention and trial completion are not appropriate for a feasibility trial because part of the primary outcome of feasibility is understanding the reasons for discontinuation. This information will be used to plan a larger randomised control trial where participant retention methods will be developed based on information from this feasibility trial.

Outcome Data to be collected for participants who discontinue/deviate from interventions protocols

Discontinuation from study intervention does not mean discontinuation of the study altogether, and the remaining study procedures, follow up assessment / visits and data collection should be completed as indicated in the protocol (unless consent is specifically withdrawn). Patients finishing the study should have an end of Study eCRF completed, (if due to patient death, there is a notification of death eCRF), detailing the reason for not continuing.

Participant Withdrawal from Follow Up visits

Patients are free to withdraw from follow up at any time without providing a reason, though a reason should be recorded if one is given. Those who wish to withdraw from further follow-up will have the data collected up to the point of that withdrawal included in the analyses. The participant will not contribute further data to the study and the CTU should be informed. The End of Study eCRF should be completed to notify the CTU of this withdrawal.

If patients express a wish to withdraw from follow up, the research team at site should ascertain if this is for all elements of trial follow-up, or if for example data from routine

For peer review only - http://bmjopen.bmj.com/site/about/guidelines.xhtml

assessments can still be collected for the trial. In the case of ongoing adverse events, participants should be given appropriate care under medical supervision until the symptoms of any adverse event resolve or the patient's condition becomes stable. Any SAEs will be notifiable to the CTU via processes detailed in Section 11 even if a participant has withdrawn from follow up.

3.11. Data management

Confidentiality

Patients will consent for the use of their data, including identifiable data such as NHS number and Date of Birth before taking part in the study. Though the consent form will have their name and NHS number, this data will not be used on any eCRF or Workbook. Patients will instead be referred to using their screening number, and then randomisation number once allocated to one of the study arms.

Consent forms with patient identifiable information will be kept within the patient file at the local research site and a copy at the LCTC to evidence that the patient consented to study. At both locations the consent forms will be stored within a locked cabinet/drawer within a secure office and department.

The LCTC as part of The University of Liverpool will preserve the confidentiality of participants taking part in the study. The University of Liverpool is registered as a Data Controller with the Information Commissioners Office.

Breaches of data protection principles or regulations identified by LCTC will be notified promptly to the trial Sponsor and The University of Liverpool's Data Protection Officer and appropriate processes followed

Quality Assurance and Control

Quality assurance will be maintained to the required level via electronic database validations, internal LCTC quality processes and procedures, training of those involved in trial procedures and regular monitoring and audit.

The TMG will be involved in monitoring screening, randomisation and consent rates, as well as compliance with processes detailed in the protocol. Any activity that is determined to be a breach of the study protocol will be recorded on a protocol deviation form and reviewed by the study CI to confirm severity, the impact on the study and whether any corrective and preventative action is required.

The LAP-PIE study will have a designated data management and query resolution plan in place and specific instructions on how to complete the eCRF forms and workbooks. A member of the study team will periodically review the study for missing or erroneous/anomalous data based on validations built into the database.

For peer review only - http://bmjopen.bmj.com/site/about/guidelines.xhtml

The frequency of the LAP-PIE Trial Management Group meeting will be outlined in the monitoring plan, along with what data in the Central Monitoring Report will be reviewed

3.12. Statistical methods

3.12.1. Outcomes

Primary Outcome

1. Recruitment rate will be measured by:

(I) the total number of patient participants randomised per month and
(ii) ratio of successful recruitment to eligible patients approached.

1. Number of screening failures.
2. Number of patients completing study pathway as per protocol will be measures as the number of randomised patients completing the full study pathway with no major protocol deviations.
3. Trial withdrawal rate will be measured using the total number of patients randomised in the study. In addition, withdrawal rates will be presented by trial withdrawal reasons and withdrawal stage for both arms.

Secondary Outcomes

1. Adherence to protocol

The number of minor and major protocol deviations will be collected on a patient participant and site level.

1. Mortality rate

Overall survival will be recorded for all patients.

1. Survival is defined as the number of days between date of randomisation and date of death due to any cause or the date of the last follow-up if the patient is still alive at the time of final trial follow up at 12 months post randomisation.
2. Survival is defined as the number of days between date of diagnosis and date of death due to any cause or the date of the last follow-up if the patient is still alive at the time of final trial follow up at 12 months post randomisation.

An overall survival rate will be calculated as also a 9-month using both definitions.

1. Technical success

Proportion of patients with a successful percutaneous IRE (complete local therapy) as defined in section 10.8 (Assessing Efficacy)

1. Surgical rate. It is possible that patients will be considered for surgery having completed second line therapy (IRE and chemo or chemo alone). If so, the following will be recorded:
2. Number of patients who become eligible for surgery over the total number of patients recruited.
3. Number of patients proceeding to surgery over the total number of patients who become eligible for surgery.

For peer review only - http://bmjopen.bmj.com/site/about/guidelines.xhtml

1. Resection rate (R0/R1)

The ratio will be calculated over numbers undergoing tumour resection in each arm of the trial. Pathology of the specimens will determine the overall R0/R1 resection rate. The R0 resection margin will be recorded according to the Royal College of Pathologists report on Standards and datasets for reporting cancers.

1. Local or systemic disease progression on follow up

The proportion of patients with local or systemic disease progression as defined by RECIST criteria in each arm of the trial will be calculated at 3,6,9 and 12 months post.

1. Chemotherapy-related toxicities.

Formal toxicity assessments will be performed and graded according to section 12. The proportion of patients experiencing grade 3/4 toxicity will be calculated.

1. IRE Procedure related complications

Complications related to IRE therapy will be recorded and the severity grading will be performed according to Clavien-Dindo classification. The proportion of patients experiencing grade III and above toxicity will be calculated.

1. Quality of life

Quality of life will be assessed with the EQ-5D-5L. The questionnaires will be completed as per the Schedule of Trial Procedures in Section 3 by all patients. The data will be scored according to the EQ-5D-5L scoring manual.

1. Return to normal activity

For peer review only

For peer review only - http://bmjopen.bmj.com/site/about/guidelines.xhtml

3.12.2. Additional analyses
Not applicable

3.12.3. Analysis population and missing data
Not applicable for a feasibility trial

3.13. Data monitoring
3.13.1. Formal committee
The Data Monitoring Committee (DMC) will consist of an independent chairperson, plus 2 independent members; who are experts in the field of Pancreatic Cancer, and an independent biostatistician.
The DMC will be responsible for reviewing and assessing recruitment, interim monitoring of safety and effectiveness, trial conduct and external data. The DMC will first convene prior to start of recruitment and will then define frequency of subsequent meetings (at least annually).

Further details of its charter can be requested from central enquires on LAP-PIE@liverpool.ac.uk

3.13.2. Interim analysis
No formal interim analyses will be performed during the course of the feasibility study. The Data and Monitoring Committee (DMC) will assess the actual recruitment rates and make decisions on whether to stop the trial in light of the analyses presented. These analyses will be performed at the LCTC. A decision to discontinue recruitment, in all patients or in selected subgroups will be made only if the result is likely to convince a broad range of clinicians including participants in the trial and the general clinical community. The DMC will make formal recommendations to the Trial Steering Committee (TSC) as to the continuation of the trial.

3.14. Safety/harms
Assessment of Complications
Adverse Events (AE) for the Standard of Care Chemotherapy will be defined using the Common Terminology Criteria for Adverse Events (CTCAE) Version 4.0 (ref). AEs for

For peer review only - http://bmjopen.bmj.com/site/about/guidelines.xhtml

IRE will be defined using the Clavien Dindo classification of surgical complications (ref). Complications greater than grade 3 on the CTCAE scale and greater than grade III on the Clavien Dindo scale will be reported.

A Serious Adverse Event (SAE) for patients randomised to receive Standard of Care Chemotherapy only, will be compared against the reference safety information (RSI) within the current summary of product characteristics (SmPC) for that drug. The RSI will be taken from the latest SmPC available for the drug on https://www.medicines.org.uk/emc. The RSI for IRE will be from the Nanoknife system users' manual.

All safety events which are recorded for the study will be reported following the procedures detailed below. The occurrence of a safety event may come to the attention of research staff during routine study visits, from the participant's notes, directly from the participant or by other means. Note that reporting procedures vary dependent on the nature of the incident (i.e. "serious" events are to be reported to LCTC in an expedited manner). Any questions concerning adverse event reporting should be directed to the LCTC in the first instance. A flowchart is provided in the additional documents to aid in determining reporting procedures for different types of adverse events.

Reporting Safety Events to the LCTC

SAE reporting will be done from the date of randomisation until the end of study period at 12 months following randomisation

All AEs, whether serious or non-serious, that occur during this timeframe will be recorded via the LCTC's pharmacovigilance database: https://lctu.org.uk/PV/login.aspx

Sites will use the LCTC pharmacovigilance database to report adverse, instruction on how to enter AEs on the pharmacovigilance database will be provided to research staff at site initiation as a work instruction. Paper CRFs will be available for download from the LCTC PORTAL for use only in case the pharmacovigilance database is not working. These can be sent in to the LCTC via Fax for upload by the LAP-PIE Study Coordinator or Data Manager.

Follow-up After Adverse Events

All reportable adverse events will be followed until satisfactory resolution or until the investigator responsible for the care of the participant deems the event to be chronic or the patient to be stable.

When reporting "serious" safety events the investigator responsible for the care of the participant will apply the following criteria to provide information relating to event outcomes:

- resolved
- resolved with sequelae (specifying with additional narrative)
- not resolved/ongoing

For peer review only - http://bmjopen.bmj.com/site/about/guidelines.xhtml

- ongoing at final follow-up
- fatal or unknown.

3.15. Auditing

Quality assurance will be maintained to the required level via electronic database validations, internal LCTC quality processes and procedures, training of those involved in trial procedures and regular monitoring and audit.

The Trial Monitoring Group (TMG) will be involved in monitoring screening, randomisation and consent rates, as well as compliance with processes detailed in the protocol. Any activity that is determined to be a breach of the study protocol will be recorded on a protocol deviation form and reviewed by the study CI to confirm severity, the impact on the study and whether any corrective and preventative action is required.

Independent oversight of the trial will be provided by the members of the DMC.

The LAP-PIE study will have a designated data management and query resolution plan in place and specific instructions on how to complete the eCRF forms and workbooks. A member of the study team will periodically review the study for missing or erroneous/anomalous data based on validations built into the database.
The frequency of the LAP-PIE TMG meeting will be outlined in the monitoring plan, along with what data in the Central Monitoring Report will be reviewed

4. ETHICS AND DISSEMINATION

4.1. Research ethics approval

Statement of Compliance

The trial involves the use of a CE marked medical device which is utilised for the intended purpose therefore this trial is not within the remit of the Medical Devices Regulation.

The procedures detailed within this protocol are compliant with the Ionising Radiation (Medical Exposure) Regulations, and appropriate review by a Medical Physics Expert and Clinical Radiation Expert has been undertaken.

Ethical Considerations

The LAP-PIE trial will be conducted in accordance with, but not limited to, the Human Rights Act 1998, the GDPR, Freedom of Information Act 2000 subject to the provisions of sections 41 and 43 thereof, the EU Clinical Trials Directive, the Medicines for Human

For peer review only - http://bmjopen.bmj.com/site/about/guidelines.xhtml

Use (Clinical Trials) Regulations 2004, the Medicines Act 1968, the Human Tissue Act 2004, ICH GCP, the Declaration of Helsinki 1996 and the NHS Research Governance Framework for Health and Social Care, as amended from time to time.

Where patients agree to take part in the study, they will be informed of how data are recorded, collected, stored and processed and may be transferred to other countries, in accordance with The General Data Protection Regulation (GDPR) (EU) 2016/679.

This study may be terminated at the request of the CI, ISDMC, REC if, during the course of the study, concerns about the safety of the study emerge.
The CI will update the ethics committee of any new information related to the intervention when appropriate and this will also be disseminated to the Principal Investigators at each trial centre.

Patients entering the trial who are randomised to have standard of care (SoC) will continue on systemic chemotherapy. As there have been no previous randomised trials of IRE for pancreas cancer, we do not know whether these patients in the SoC arm will have a better or worse survival and quality of life than those receiving IRE.

The trial will abide by the principles of the World Medical Association Declaration of Helsinki and has been designed to be as pragmatic as possible. The protocol has undergone ethical review by an independent Research Ethics Committee and has received a favourable opinion by Brent REC.

Approvals

The trial protocol will receive approval by a National Research Ethics Committee (NREC), but all participating sites must undergo site specific assessment of capacity and capability. A copy of all site approval documents and a copy of the PIS and ICF on local headed paper should be forwarded to LCTC before patients are entered. The LCTC should receive a confirmation of capacity and capability for each new centre via the site's R&D department

4.2. Protocol amendments

4.3. Informed consent process

Written informed consent is required for all patients participating in the LAP-PIE trial. Discussion of objectives, risks and inconveniences of the trial and the conditions under which it is to be conducted are to be provided to patients by staff with appropriate experience. An appropriate Patient Information Sheet (PIS), describing in detail the trial interventions/products, trial procedures and risks will be approved by an independent ethical committee (IEC) and the patient will be asked to read and review the document. Upon reviewing the document, the investigator will explain the research study to the patient and answer any questions that may arise. A contact point where further information about the trial may be obtained will be provided.

For peer review only - http://bmjopen.bmj.com/site/about/guidelines.xhtml

In obtaining and documenting consent, the research team will comply with applicable regulatory requirements and will adhere to GCP and to the ethical principles that have their origin in the Declaration of Helsinki.

Informed consent will be re-affirmed throughout the trial and all discussions and consent will be documented appropriately. If a potential participant does not want to provide consent, they do not have to give a reason.

Written informed consent will be sought from patients who will be approached by the study team and invited to consider participation. This is a discussion that will take place within the hospital that the patient is attending for their LAPC treatment and assessments.

The PIS includes a detailed explanation of the study and makes clear that the rights and welfare of the participants will be protected; it will be emphasised that consent may be declined or withdrawn at any time in the future without the quality of care being adversely affected. The research team will facilitate verbal discussions about the research and the consent process, as well as providing answers to any questions that arise.

All the points on the main study section of the consent form are mandatory for the patient to be eligible, they may not agree to only a selection of criteria. There are optional statements on the consent form related to the translational sub studies and the qualitative evaluation. Patients who do not wish to participate in the sub studies or qualitative evaluation are still eligible for the LAP-PIE trial.

After verbal and written information has been provided, the individual seeking consent must ensure that the patient has fully understood all the information and will ask if they are happy to consent to participation in the trial. Where this is the case, written informed consent will be obtained by means of a dated patient signature on the consent form. This will be countersigned and dated by the person who obtained informed consent i.e. the PI or other appropriately qualified member of the research team who has been delegated this responsibility.

The person obtaining consent, i.e. the person signing off on the consent form with the patient, must be the Principle Investigator or delegated other, a medical practitioner capable of explaining the study rationale and the treatment options to the patient.

The original signed document will be retained in the trial site's Investigator Site File (ISF) and copies will be made:

- One copy provided to the patient for their information,
- One copy transferred to the LCTC
- One copy filed in the participant's medical records.

For peer review only - http://bmjopen.bmj.com/site/about/guidelines.xhtml

N.B. Details of the consent process (date, persons involved, version and type of information sheet and consent form used) must also be recorded directly into the participant's medical records.

4.4. Confidentiality

Patients will consent for the use of their data, including identifiable data such as NHS number and Date of Birth before taking part in the study. Though the consent form will have their name and NHS number, this data will not be used on any eCRF or Workbook. Patients will instead be referred to using their screening number, and then randomisation number once allocated to one of the study arms.

Consent forms with patient identifiable information will be kept within the patient file at the local research site and a copy at the LCTC to evidence that the patient consented to study. At both locations the consent forms will be stored within a locked cabinet/drawer within a secure office and department.

The LCTC as part of The University of Liverpool will preserve the confidentiality of participants taking part in the study. The University of Liverpool is registered as a Data Controller with the Information Commissioners Office.

Breaches of data protection principles or regulations identified by LCTC will be notified promptly to the trial Sponsor and The University of Liverpool's Data Protection Officer and appropriate processes followed.

Records Retention

The investigator at each investigational site will make arrangements to store the essential trial documents, including the Investigator Trial File, until the LCTC informs the investigator that the documents are no longer to be retained.

The PI at each investigational site will make arrangements to store the essential trial documents (as defined by ICH GCP guidelines) including the Investigator Site File, the applicable participant medical records for the full length of the trial's retention period and will arrange for confidential destruction at the end of this period as instructed by the Sponsor.

The PI is also responsible for archiving all relevant source documents so that the trial data can be compared against source data after completion of the trial (e.g. in case of inspection from authorities). They must ensure the continued storage of the documents, even if they, for example, leave the clinic/practice or retire before the end of required storage period. Delegation of responsibility for this must be documented in writing.

4.5. Declaration of interests

For peer review only - http://bmjopen.bmj.com/site/about/guidelines.xhtml

None declared

4.6. Access to data

The Trial Management Group (TMG) will have access to the final trial dataset. The TMG consists of the Chief Investigator, other lead investigators (clinical and non-clinical) and members of the Liverpool Clinical Trials Centre.
Individual Clinicians must undertake not to submit any part of their individual data for publication without the prior consent of the Trial Management Group.

4.7. Dissemination policy

4.7.1. Trial results

The results from different centres will be analysed together and published as soon as possible following completion of the recruitment and follow up period. Individual Clinicians must undertake not to submit any part of their individual data for publication without the prior consent of the Trial Management Group.
The Trial Management Group will form the basis of the Writing Committee and advise on the nature of publications. The Uniform Requirements for Manuscripts Submitted to Biomedical Journals (http://www.icmje.org/) will be respected. . The ISRCTN allocated to this trial will be attached to any publications resulting from this trial.

All publications and submission for presentation will be approved by NIHR and must contain a statement of responsibility as per NIHR standard guidance.

4.7.2. Authorship

All publications will include a list of participants, and if there are named authors, these will include the trial's Chief Investigator(s), Statistician(s) and Trial Manager(s) involved at least. If there are no named authors (i.e. group authorship) then a writing committee will be identified that will include these people, at least.

4.7.3. Reproducible research

On completion of the research, a Final Trial Report will be prepared and submitted to the REC. The results of LAP-PIE will be published regardless of the results.

There are no plans for granting public access, however data sharing will be supported via access through the Trial Management Group (TMG).

5. STUDY ADMINISTRATION

5.1. Key contacts

Study Principal Investigator

Brian R. Davidson, MB ChB, MD, FRCS (Eng), FRCPS (Glasg)
Division of Surgery and Interventional Science, University College London
Royal Free Hospital, 9th Floor (East), 2QG, 10 Pond St, London NW3 2PS
020 7794 0500, Ext.33603

For peer review only - http://bmjopen.bmj.com/site/about/guidelines.xhtml

b.davidson@ucl.ac.uk

Central contact
Operational Director

Liverpool Clinical Trials Centre Block C, Waterhouse Building 1-3 Brownlow Street
Liverpool L69 3GL
0151 794 8897
LAP-PIE@liverpool.ac.uk

Sponsor
Research & Development Office Lower Ground Floor Finance Office (LG/306)
Royal Free London NHS Foundation Trust
Pond Street London
London, London, NW3 2QG
United Kingdom
020 7317 7558
rf.randd@nhs.net

For peer review only

5.2. Funders

Sources and types of financial, material, and other support
Funding body: National Institute for Health Research (NIHR), Research for Patient Benefit (RfPB) programme
Central Commissioning Facility
Grange House
15 Church Street
Twickenham
TW1 3NL
Training support: Angiodynamics. Manufacturer of CE marked device that is in use in clinics already. Angiodynamics will provide training and support as normal during this study and supply of the needle probes used through the duration.
Other Support. Operational Director
Liverpool Clinical Trials Centre
Block C, Waterhouse Building
1-3 Brownlow Street
Liverpool
L69 3GL
Tel: 0151 794 8897
Fax: 0151 794 8931
LAP-PIE@liverpool.ac.uk

5.3. Roles and responsibilities

5.3.1. Protocol contributors

For peer review only - http://bmjopen.bmj.com/site/about/guidelines.xhtml

Zainab L Rai, Veronica Ranieri, Stackpoole M, Littler P, Palmer D, Paula Ghaneh, Gurusamy K, Manas D, Elena Pizzo, Eftychia E Psarelli, Davidson B and the UK LAP-PIE study group

Acknowledgement
This study is funded by the UK NIHR RfPB programme (PB-PG-1217-20042).
The views expressed are those of the author(s) and not necessarily those of the NHS, the NIHR or the Department of Health.

5.3.2. Sponsor and funder

Study sponsor and funder had no role in the study design, data collection and management, data analysis and interpretation and were not involved in the decision to submit report for publication. They do not have ultimate authority over any of these activities.

5.3.3. Trial committees

Liverpool Clinical Trial Centre (LCTC) at the University of Liverpool in collaboration with the Chief Investigator, will have overall management responsibility and will be responsible for trial management activities including (but not limited to), budget administration, Trial Master File management, safety reporting, data management, randomisation, statistical analysis, participating site coordination and IMP management.

Trial Management Group (TMG)

A Trial Management Group (TMG) will be formed comprising the Chief Investigator, other lead investigators (clinical and non-clinical) and members of the LCTC. A representative from the Sponsor will also be part of the meeting, and any reports or minutes generated will be circulated for their review. The TMG are responsible for monitoring all aspects of the progress and conduct of the trial and will be responsible for the day-to-day running and management of the trial. The TMG will meet fortnightly throughout the study unless more frequent meetings are required.

For peer review only - http://bmjopen.bmj.com/site/about/guidelines.xhtml

6. REFERENCES

(1) Ghaneh P, Costello E, Neoptolemos JP. Biology and management of pancreatic cancer. *Postgrad Med J* 2008;84(995):478-97.
(2) Balaban EP, Mangu PB, Khorana AA, et al. Locally Advanced, Unresectable Pancreatic Cancer: American Society of Clinical Oncology Clinical Practice Guideline. *J Clin Oncol.* 2016;34(22):2654-68.
(3) Ghosn M, Ibrahim T, Assi T, et al. Dilemma of first line regimens in metastatic pancreatic adenocarcinoma. *World J Gastroenterol* 2016;22(46):10124-30.
(4) Rahib L, Fleshman JM, Matrisian LM, et al. Evaluation of Pancreatic Cancer Clinical Trials and Benchmarks for Clinically Meaningful Future Trials: A Systematic Review. *JAMA Oncol* 2016;2(9):1209-16.
(5) Wang C, Liu X, Wang X, et al. Effects of chemoradiotherapy and chemotherapy on survival of patients with locally advanced pancreatic cancer: A meta-analysis of randomized controlled trials. *Medicine (Baltimore)* 2018;97(36):
(6) Excellence NIfHaC. Overview | Guidance | NICE: NICE; [Available from: Pancreatic cancer in adults: diagnosis and management |. 2020. [Cited: 10/2020]. Available from: https://www.nice.org.uk/guidance/ng85..
(7) V P, P F, P R, et al. Combination of Irinotecan (CPT11) and 5-fluorouracil With an Analysis of Cellular Determinants of Drug Activity. *Biochemical pharmacology.* 1998;56(10):
(8) M D, E M, M O-K, et al. Randomized Phase II Study Evaluating Oxaliplatin Alone, Oxaliplatin Combined with Infusional 5-FU, and Infusional 5-FU Alone in Advanced Pancreatic Carcinoma Patients. *Annals of oncology: official journal of the European Society for Medical Oncology.* 2004;15(3):
(9) Suker M, Beumer BR, Sadot E, et al. A patient-level meta-analysis of FOLFIRINOX for locally advanced pancreatic cancer. *Lancet Oncol.* 2016;17(6):801-10.
(10) Mansson C, Brahmstaedt R, Nygren P, et al. Percutaneous Irreversible Electroporation as First-line Treatment of Locally Advanced Pancreatic Cancer. *Anticancer Res* 2019;39(5):2509-12.
(11) Belfiore MP, Ronza FM, Romano F, et al. Percutaneous CT-guided irreversible electroporation followed by chemotherapy as a novel neoadjuvant protocol in locally advanced pancreatic cancer: Our preliminary experience. *Int J Surg* 2015;21(Suppl 1):34-9.
(12) Lafranceschina S, Brunetti O, Delvecchio A, et al. Systematic Review of Irreversible Electroporation Role in Management of Locally Advanced Pancreatic Cancer. *Cancers (Basel)* 2019;11(11):7181.
(13) Hajatdoost L, Sedaghat K, Walker EJ, et al. Chemotherapy in Pancreatic Cancer: A Systematic Review. *Medicina (Kaunas)* 2018;54(3):48.
(14) MP C, KJ C, EK F, et al. Pre-treatment Assessment of Resectable and Borderline Resectable Pancreatic Cancer: Expert Consensus Statement. *Annals of surgical oncology* 2009;16(7):
(15) Network NCC. Pancreatic Adenocarcinoma, Version 2.2017, NCCN Clinical Practice Guidelines in Oncology - NCCN-2017-pancreatic-cancer.pdf: NCCN; 2017 [Available from: http://clubedopancreas.pt/wp-content/uploads/2018/06/NCCN-2017-pancreatic-cancer.pdf.

For peer review only - http://bmjopen.bmj.com/site/about/guidelines.xhtml

(16) Excellence NIfHaC. Recommendations. Pancreatic cancer in adults: diagnosis and management | Guidance | NICE: NICE. 2020. [Cited: 10/2020]. Available from: https://www.nice.org.uk/guidance/ng85/chapter/Recommendations#managing-resectable-and-borderline-resectable-pancreatic-cancer..
(17) Wills-Percy. St Luke's Cancer Alliance: Cancer treatment 2018. 2018. [Cited: 10/2020]. Available from: http://stlukescanceralliance.co.uk/wp-content/uploads/2018/08/Gem-Cap-V3-8.18.pdf.
(18) Hurt CN, Falk S, Crosby T, et al. Long-term results and recurrence patterns from SCALOP: a phase II randomised trial of gemcitabine- or capecitabine-based chemoradiation for locally advanced pancreatic cancer. *Br J Cancer* 2017;116(10):1264-70.
(19) He C, Wang J, Sun S, et al. Irreversible electroporation versus radiotherapy after induction chemotherapy on survival in patients with locally advanced pancreatic cancer: a propensity score analysis. *BMC Cancer.* 2019;19(394):
(20) Narayanan G, Hosein PJ, Beulaygue IC, et al. Percutaneous Image-Guided Irreversible Electroporation for the Treatment of Unresectable, Locally Advanced Pancreatic Adenocarcinoma. *J Vasc Interv Radiol.* 2017;28(3):342-8.
(21) Kluger MD, Epelboym I, Schrope BA, et al. Single-Institution Experience with Irreversible Electroporation for T4 Pancreatic Cancer: First 50 Patients. *Ann Surg Oncol.* 2016;23(5):1736-43.
(22) Liu S, Qin Z, Xu J, et al. Irreversible electroporation combined with chemotherapy for unresectable pancreatic carcinoma: a prospective cohort study. *Onco Targets Ther* 2019;12(5):1341-50.
(23) W F, JW R, RCG M. Quality of Life Assessment for Patients Undergoing Irreversible Electroporation (IRE) for Treatment of Locally Advanced Pancreatic Cancer (LAPC). *American journal of surgery.* 2019;218(3):
(24) Julious SA.. Sample size of 12 per group rule of thumb for a pilot study.. *Pharmaceutical Statistics* 2005;4(4):287-91.
(25) Sim J, Lewis M.. The size of a pilot study for a clinical trial should be calculated in relation to considerations of precision and efficiency.. *J Clin Epidemiology.* 2012;65(3):301-8.

For peer review only

For peer review only - http://bmjopen.bmj.com/site/about/guidelines.xhtml

7. APPENDICES

7.1. Informed consent materials

**PATIENT CONSENT FORM (please read carefully)**
**LAP-PIE:** Treatment of unresectable **L**ocally **A**dvanced **P**ancreatic cancer with **P**ercutaneous **I**rreversible **E**lectroporation (IRE) following initial systemic chemotherapy: a feasibility randomised controlled trial<br>**Study ID:** ________________<br>**NHS Number** ____________________

**Name of Researcher: ______________________________**	
**Please initial each box**	
**1. I confirm that I have read and understand the patient information sheet date: ………………….. (Version: …………) describing the above study and have had the opportunity to consider the information, ask questions and have had these answered to my satisfaction.**	

For peer review only

**2. I understand that my participation in this study is voluntary and that I am free to withdraw at any time without giving a reason, without my medical care or legal rights being affected.**	
**3. I understand that sections of my medical notes and data collected during the study may be looked at by responsible individuals involved in this research at my hospital, the University of Liverpool, or from regulatory authorities where it is relevant to my taking part in research. I give permission for these individuals to have access to my records.**	

For peer review only

For peer review only - http://bmjopen.bmj.com/site/about/guidelines.xhtml

**4. I understand that imaging (Xray's, CT scans etc.) and blood test results will be reviewed by a central research team in order to assess success of the treatment I receive.**	
**5. I agree to allow my General Practitioner and any other relevant medical practitioner to be informed of my involvement in the study.**	

For peer review only

For peer review only - http://bmjopen.bmj.com/site/about/guidelines.xhtml

**6. I am aware that there is a qualitative aspect to this study and that patients may be selected at random to discuss their involvement and understanding of the study with a dedicated qualitative researcher. I agree to take part in these interviews if selected.**	
**7. I agree for a copy of this completed consent form to be sent to the University of Liverpool Clinical Trials Centre (where it will be kept in a secure location), to allow confirmation that my consent for the trial has been given.**	

For peer review only

For peer review only - http://bmjopen.bmj.com/site/about/guidelines.xhtml

**8. I understand that information held by the NHS and records maintained by the NHS Information Centre may be used to keep in touch with me and follow-up my health status.**		
**9. I agree that data from the study can be used in future research.**<br><br>10. I am aware that once registered on to the study there is no way to predict or influence which arm I am randomised to.		
11. I agree to take part in the LAP-PIE study		
**This part of the trial is OPTIONAL. Only initial YES if you wish to give permission. Initial NO if you do not give permission.**		
• I give permission for the samples of my blood/urine/tissue specimens to be taken and used for this trial and be transferred to research institutes in the UK and the rest of the world.	**Yes**	**No**
• I give permission for the samples of my blood/urine/tissue specimens to be used in future research and be transferred to research institutes in the UK and the rest of the world.	**Yes**	**No**
• I give permission for my stored pathological specimens to be used for this trial and be transferred to research institutes in the UK and the rest of the world.	**Yes**	**No**

For peer review only - http://bmjopen.bmj.com/site/about/guidelines.xhtml

• I give permission for my stored pathological specimens to be used for future research and be transferred to research institutes in the UK and the rest of the world.	**Yes**	**No**
• I give permission for genetic material (DNA and RNA) to be extracted from blood and cancer tissue specimens for current ethically approved research.	**Yes**	**No**
• I give permission for genetic material (DNA and RNA) to be extracted from blood and cancer tissue specimens for future ethically approved research.	**Yes**	**No**
• I agree that data from the study can be sent outside the European Economic Area (EEA).	**Yes**	**No**

**Name of patient**		**Date**		**Signature**
**Researcher**		**Date**		**Signature**
**Name of person taking consent (if different from researcher)**		**Date**		**Signature**

Please ensure the dates of consent are the same date for everyone, this form needs to be signed by both the patient and the person obtaining consent. If there are any mistake made that need to be corrected, please draw a line through and write the correction next to it; initial and date the correction. One copy of this form is given to the patient, one kept in the Patient File at the Research Site, and one copy is sent to the LCTC

For peer review only - http://bmjopen.bmj.com/site/about/guidelines.xhtml

PATIENT INFORMATION SHEET

Treatment of unresectable **L**ocally **A**dvanced **P**ancreatic cancer with **P**ercutaneous **I**rreversible **E**lectroporation (IRE) following initial systemic chemotherapy: a feasibility randomised controlled trial

You have been invited to take part in a research study. Before you decide, it is important that you understand why the research is being done and what it will involve. Please take time to read the following information carefully and discuss it with others if you wish.

- **Part One** tells you the purpose of the study and what will happen to you if you take part.
- **Part Two** gives you more detailed information about the conduct of the study.

The clinical team in change of your care will go through the details with you, but please ask us if there is anything that is not clear or if you would like more information. Take time to decide whether or not you wish to take part.
Thank you for reading this.

<u>Part One</u>

Please note that Locally Advanced Pancreatic Cancer has been shortened to LAPC throughout this information sheet.

What is the purpose of the study?

The Purpose of the LAP-PIE study is to see how well we are able to recruit patients to a study of this type, and to determine if a new type of cancer treatment is effective in reducing the size of a Pancreatic tumour to the point that it can be surgically removed. The treatment is known as Irreversible Electroporation (IRE), which essentially uses electric pulses to destroy the cancer cells whilst leaving the healthy cells unharmed. We are recruiting patients in order to see how effective this treatment option is compared to standard chemotherapy in patients, and at the same time look at issues in recruitment that will help us design a larger, more long-term study with the same goals.

Why have I been chosen?

You have been chosen as you are a patient who has been diagnosed with advanced pancreatic cancer that is not yet suitable for surgery. In addition, you are either due to start FOLFIRINOX® chemotherapy through the NHS, are currently having it, or at the end of treatment. You were approached by a member of the study team in a hospital that is currently running the LAP-PIE study and have been identified to meet some of our main inclusion criteria.

Do I have to take part?

For peer review only - http://bmjopen.bmj.com/site/about/guidelines.xhtml

No. It is up to you to decide whether or not to take part. If you do agree you will be asked to sign a consent form, but you are free to withdraw at any time afterwards without giving a reason. A decision to withdraw at any time, or a decision not to take part, will not affect the standard of care you receive.

What will happen to me if I take part?

From Randomisation, you will be on study for however long your second line chemotherapy lasts and a further 12 months for follow up.

The visits that you will attend are those that you would normally be required to make for treatment of LAPC; They may last slightly longer for some additional tests and for the research team to record the results of assessments for the study.

The results of standard of care tests such as blood, CT scans and biopsies will also be collected for the purposes of the study. All data is anonymised and kept on a secure system at the Liverpool Clinical Trials Centre.

If you are admitted to hospital for any reason during your time on the study, the details of the visit will be recorded and reviewed by the study team to ensure it is not related to the study itself.

At each visit you will be asked if you have experienced any adverse events or conditions or if you are taking any new medication since the last time you were seen.

What do I have to do?

If you become a patient on this study then you will be first asked to sign a consent form confirming you have read this form and spoken to the research team about the study and are willing to take part. Giving consent to the study means that you understand the assessments and visits necessary and are willing to adhere to the study protocol. You will then have some assessments done to confirm that you are eligible, many of them are tests you will already be having as a part of your standard care. You will have blood taken for laboratory assessment and will have an X-Ray of your chest and CT scan of your abdomen, pelvis and chest. All patients of childbearing potential will be given a test to confirm they are not pregnant before starting the study. There is also a quality of life questionnaire to complete which will ask you to record how you are feeling and describe your overall health.

Some patients will be selected for interviews with a qualitative researcher, which will be a discussion of your understanding of the study and how it was communicated to you. The interviewer will also ask you about your knowledge of the treatment available and your condition. This interview may be done over the phone, or in person during one of your visits to the hospital you are being treated at.

The results of these test will be sent to the LCTC who are running the study who will randomised you using a computer system to one of two arms, both arms are receiving the same standard of care that would be given, however one arm will receive on IRE procedure (more on this below).

For peer review only - http://bmjopen.bmj.com/site/about/guidelines.xhtml

Following randomisation, you will need to attend hospital to receive the therapy that your local oncologist recommends and adhere to all of the test and visits related to it. The choice of chemotherapy that you will be given is the same that would have been chosen regardless of whether you were on this study. Data on the tests and visits you attend will be recorded by the research team and given to the LCTC for the study results. Half of the patients randomised will also receive on IRE procedure at their recruiting hospital trust.

Patients receiving the IRE will be admitted to hospital for a few days during their second line chemotherapy where they will be given the IRE procedure. IRE will involve some pre procedure assessments and some after care in hospital. More details on the procedure are below.

At the end of chemotherapy, you will be reviewed to see if your LAPC is now suitable for surgery. If suitable you will be given the same procedure in hospital as normal for patients with LAPC.

Following the surgery and after caring you will need to attend hospital every 3 months for one year. At these visits you will have bloods taken, a physical exam and there will be X-Rays and CT scans done. You will also be given a quality of life questionnaire to complete at these visits.

Should you be unsuitable for IRE before you are meant to have it, or if your LAPC is not suitable for surgery after finishing chemotherapy, or your oncologist decides to take you off of the chemotherapy for whatever reason, you will still be given the option to remain on follow up for 12 months.

At the end of your involvement with the study, you will be given some final assessments and will not have to attend for any LAP-PIE related assessment, and no further data will be collected about you. Your involvement ends at the last follow up visit, or when you choose to not take part any further.

What is the Treatment that is being tested?

Irreversible Electroporation is a fairly new device, though has been licensed for use on the NHS and in patients with Locally Advanced Pancreatic Cancer. IRE is only routinely available on the NHS for patients with this disease at certain hospitals in the UK, such as the hospital you are attending.

IRE is delivered by inserting long needle probes through the skin and into the pancreas, the ends of them will surround the tumour. Once in place the generator on the IRE device will send electrical impulses down the needles and around the cancerous tissue. The pulses heat the cells and weaken their cell membranes, so they break down over around a 24-hour period following the treatment. IRE has been used previously in treating a number of types of cancer, such as Liver, Pancreatic and Prostate cancers on an experimental basis. IRE is used in order to reduce the size of a tumour so that it is

For peer review only - http://bmjopen.bmj.com/site/about/guidelines.xhtml

more suitable for surgical removal. It is different from other similar treatments in that is does not generate heat, which can cause damage to surrounding tissue.

The needles will be guided using a CT scan, and not open surgery, meaning there will be less recovery time. There will only be one 2-hour long procedure involving IRE for patients in this study. Before the procedure you will have a blood test and a further test 6 hours after your procedure ends. You will be admitted to hospital for a few nights for monitoring until deemed suitable for discharge, you will likely be prescribed pain killers to take as well. 6 days after your IRE treatment you will be asked to return for another blood test.

As IRE uses electricity, a heart monitor will be used to time each pulse, so it does not affect your hearts rhythm. Patients who have pacemakers or other indwelling electrical devices are therefore not suitable for this study.

All patients who take part in this study will still receive chemotherapy as normally indicated for their care, prior to receiving either chemotherapy plus IRE, or just more chemotherapy. It is not known at this time, whether the combination of IRE and Chemotherapy, or just Chemotherapy is more effective at reducing tumour size in LAPC to make it suitable for surgery. This study aims to look at how easy or difficult it is to recruit patients such as yourself and randomise to one option or the other before pancreatic surgery. The side effects, and overall quality of life of patients across the two groups will be compared too. The findings will guide a large study that will compare the two options with greater accuracy.

What are the alternatives for treatment?

Advanced Pancreatic Cancer that is not suitable for surgery is currently treated on the NHS using: Chemotherapy, Chemotherapy and Radiotherapy, or with Stereotactic body radiotherapy (essentially a more targeted, precise way of delivering radiotherapy). Each of these methods have their own levels of efficacy and side effects and given as needed depending on other patient factors such as overall health and the degree of Pancreatic Cancer.
All chemotherapy drugs have their own side effects, mostly nausea and fatigue as well as affecting your appetite. Radiotherapy is also known to carry these side effects and can also cause skin reactions in some cases. Some other devices are available that use direct heat to destroy cancerous tissue, however the temperatures are far greater than those used in IRE and can cause damage to the healthy surrounding tissue.

Your doctor will be able to give you more detail on these treatments.

What are the side effects of the treatment I will receive when taking part?

Patients who are enrolled to this study will receive, or have received, chemotherapy (Folfirinox®) for 6 weeks, before being randomised to Chemotherapy plus IRE, or just

For peer review only - http://bmjopen.bmj.com/site/about/guidelines.xhtml

chemotherapy. The side effects of the Folfirinox and whichever NICE approved chemotherapy given after will be well known and described to you by your oncologist.

The Side effects of the IRE based on early studies are:

- Some pain, though this can be managed with painkillers and will not normally last more than a few days
- Possible pancreatitis; this is an inflammation of the pancreas and can usually be managed with pain relief and you may need to spend a few days in hospital. If more severe this stay in hospital could extend to a few weeks or months, though this is not common
- Some problems with eating or drinking; you could have a diminished appetite or feel sick, or possibly experience heartburn. These symptoms do not normally last long
- A blood clot could develop in a vein near where the IRE was performed. This is rare and can be treated with medication, though would mean you may not be able to have surgery after the IRE, this will be for the doctor at site to decide. Standard practice with IRE is to administer drugs that reduce the risk of clots forming.
- In some very rare cases there may be some leaking of fluid from the bile duct or duodenum (the start of the small intestine). This can be caused by the needles that are used in IRE damaging these areas.

What are the possible disadvantages and risks of taking part?

This study is only recruiting patients who have been given Folfirinox as first line therapy and then their oncologist's choice for second line treatment. Therefore, the side effects and disadvantages of the chemotherapy aspect are the same as they would be regardless of entering the study. The potential side effects and risks associated with IRE are also listed above. It is possible that after undergoing either treatment option then your LAPC will still not be suitable for surgery. There is also always the possibility with any standard of care treatment that side effects, or complications, mean you will have to stop the therapy and another option will have to be considered.

There will also be the need to attend clinic on certain days that cannot be planned out at this stage, which will most likely be during working hours. There will also be more questions about your health and wellbeing asked at each routine visit in order to record the study data. Though most of the tests you will have are already part of standard of care, there will be some extra blood samples taken at various points in the study; all effort will be made to have these done at the same time as the routine bloods for your convenience.

What are the possible benefits of taking part?

We hope that the treatments will help you. However, this cannot be guaranteed. The information we get from this study may help us to improve the future treatment of patients with Advanced Pancreatic Cancer. The results of this feasibility study will also

For peer review only - http://bmjopen.bmj.com/site/about/guidelines.xhtml

help to develop a larger clinical trial into the use of IRE in patients with the same condition.

What happens when the research study stops?
It should be made clear if participants will have continued access to any benefits or interventions obtained during the research. If treatment is unavailable after the trial finishes, this should be explained, and information given on what treatment is available.

The chemotherapy that you will be given from this study are already approved and supplied via standard of care, and therefore will still be available should the study be terminated for any reason at your hospital.

What if there is a problem?
Any complaint about the way you have been dealt with during the study or any possible harm you might suffer will be addressed. The detailed information is given in Part 2 of this information sheet.

Will my taking part in this study be kept confidential?
Yes. All the confidential information about your participation in this study will be kept confidential. The detailed information on this is given in Part 2.

Contact for Further Information
Should you have any further queries regarding this study or about any of the treatments described above,

Please contact:
Appropriate job title:
[Trust to insert contact details]
Contact Number is:
[Trust to insert contact details]

This completes Part One of the Information Sheet. If the information in Part One has interested you and you are considering participation, please continue to read the additional information in Part Two before making any decision.

Part Two

What if new information becomes available?
Sometimes during the course of a research project, new information becomes available about the treatment/drug that is being studied. If this happens, your doctor will tell you about it and discuss with you whether you want to or should continue in the study. If you decide to withdraw your doctor will make arrangements for your care to continue. If you decide to continue in the study you will be asked to sign an updated consent form.

For peer review only - http://bmjopen.bmj.com/site/about/guidelines.xhtml

On receiving new information your doctor might consider it to be in your best interests to withdraw you from the study. He/she will explain the reasons and arrange for your care to continue.
If the study is stopped for any other reason you will be told why, and your continuing care will be arranged.

What will happen if I don't want to carry on with the study?

You retain the right to decide how much of the study you wish to participate in at all times. If there is any part of it you are not happy with you can choose not to have any test, give blood sample or answer any question. If you wish to no longer continue on the study this is your decision and will be respected. If you wish to stop during the chemotherapy phase then you will not be randomised to receive the IRE and may discuss with your doctor what you wish to do next. In all cases data and samples taken for patients who are withdrawn will be kept so they can still be used to help the study. At any time, you may tell the research team that you want any of your data and samples destroyed or not used in the research.

If you do not wish to attend the follow up visits this is entirely up to you. Every effort will be made to still contact you, or your GP for some data, or you may even decide that you no longer wish to be involved in the study at all.

What if there is a problem?
If you have a concern about any aspect of this study, you should ask to speak with the researchers who will do their best to answer your questions. If you remain unhappy and wish to complain formally, you can do this through the normal NHS Complaints Procedure. Details can be obtained from the hospital.
If you are harmed by taking part in this research project and this is due to someone's negligence, then you may have grounds for a legal action for compensation against the NHS Trust where you are being treated, but you may have to pay for your legal costs. The normal National Health Service complaints mechanisms should be available to you (if appropriate).

If you suffer harm arising from the negligent management or negligent design of the research you will have grounds to claim compensation. In the absence of negligence compensation is not payable.
If you experience non-negligent harm arising from the management or design of the research then The University of Liverpool clinical trials insurance may apply for this investigator-initiated study.
In the event of defective product then you may have grounds for a legal action for compensation against the manufacturer, but you may have to pay for your legal costs.

Will my taking part in this study be kept confidential?
The Royal Free Hospital is the sponsor for this study based in the United Kingdom. We will be using information from you and your medical records in order to undertake this study and will act as the data controller for this study. This means that we are

For peer review only - http://bmjopen.bmj.com/site/about/guidelines.xhtml

responsible for looking after your information and using it properly. [Sponsor organisation] will keep identifiable information about you [for x years after the study has finished/ until x].

Your rights to access, change or move your information are limited, as we need to manage your information in specific ways in order for the research to be reliable and accurate. If you withdraw from the study, we will keep the information about you that we have already obtained. To safeguard your rights, we will use the minimum personally identifiable information possible.

If you join the study, some parts of your medical records and the data collected for the study will be looked at by authorised persons from Cancer Research UK or their collaborators who are also involved in organising this research project and may be used in a number of research projects and/or for research related to the development of pharmaceutical products, diagnostics or medical aids by other scientists and research organisations within and outside the European Economic Area, in universities, the NHS or commercial companies involved in medical research worldwide which may also involve discussions with regulatory authorities. Patients' safety data may be reviewed outside the EU, but no one will be able to identify you. Data may also be looked at by representatives of regulatory authorities and by authorised people from the Trust, other NHS bodies or the commercial companies referred to above to check that the study is being carried out correctly. All will have a duty of confidentiality to you as a research participant.

A copy of your completed consent form will be sent to the Liverpool Cancer Trial Unit to allow them to check you have agreed to the trial. This will be kept in a secure location away from all the study data.

Involvement of the General Practitioner/Family Doctor (GP)
With your consent, your GP will be informed of your involvement in the trial. Any other medical practitioners who treat you, e.g. should you be admitted to hospital for any reason, will also be informed.

What will happen to any samples I give?
Some of your samples will be analysed at your hospital for the purpose of your treatment.
With your permission,. The researchers at the University of Liverpool work closely with other scientists in the UK and around Europe and, with your permission, your samples may be transferred to these research collaborators for use in future scientific studies. These samples will be used only for investigating Locally Advance Pancreatic Cancer and will not be used for any commercial purposes.
The samples will be kept in a secure place until we need them; nobody outside of the study will have access to **any** confidential information that you give to us. Confidential

For peer review only - http://bmjopen.bmj.com/site/about/guidelines.xhtml

details (such as your name, address and GP details) will be kept locally and not made available to collaborators.
Your sample will be coded and the researchers carrying out tests on the samples will not be given information they do not need to carry out the tests and analyse the results. Coded is not the same as anonymous. It will be possible to use the codes to identify that a result is from your sample. However, we do not plan to do this unless there is a good reason to do so. We will maintain this information so that we can properly manage the samples donated. For instance, sometimes we may need to update our record of your clinical details to help us interpret the results of tests.

What will happen to the results of the research study?
It is intended that once the study is complete a report will be written, and the results will be published to make them available to the public. They may also be used to apply to the regulatory authorities to make the drug widely available and/or for research related to the development of pharmaceutical products, diagnostics or medical aids. You will not be named or identified in any publication.

What rights do I have to the results of the research?
Any information derived directly or indirectly from this research, as well as any patents, diagnostic tests, drugs, or biological products developed directly or indirectly as a
result of this research may be used for commercial purposes. You have no right to this property or to any share of the profits that may be earned directly or indirectly as a result of this research. However, in signing this form and donating tissue and/or blood samples for this research, you do not give up any rights that you would otherwise have as a participant in research.

Who is organising and funding the research?

The study is funded by the NIHR and the equipment and training needed for IRE is being provided by Angiodynamics, the company who developed the technology.

Your doctor will not receive any payment for including you in this study.
The study is funded by the NIHR, the governing body that oversees and supports research done through the NHS. The money given a grant from them is to support the cost of performing the research such as the assessments, set up costs and time needed working on the study. No one involved in the research receives any payment for being involved, nor incentive to encourage recruitment to the LAP-PIE study.

Who has reviewed the study?
The study has been reviewed for scientific content by members of London Brent Research Ethics Committee (REC) has for ethical considerations and has a favourable opinion.
Thank you for taking the time to read and consider this information sheet. Should you decide to take part in the study, you will be given a copy of the information sheet and a signed consent form to keep.

For peer review only - http://bmjopen.bmj.com/site/about/guidelines.xhtml

For peer review only

For peer review only - http://bmjopen.bmj.com/site/about/guidelines.xhtml

BMJ Open

Treatment of unresectable Locally Advanced Pancreatic cancer with Percutaneous Irreversible Electroporation (IRE) following initial systemic chemotherapy (LAP-PIE) trial: Study protocol for a feasibility randomised controlled trial.

Journal:	*BMJ Open*
Manuscript ID	bmjopen-2021-050166.R1
Article Type:	Protocol
Date Submitted by the Author:	23-Aug-2021
Complete List of Authors:	Rai, Zainab; University College London, Division of Surgery and Interventional Science; Wellcome / EPSRC Centre for Interventional and Surgical Sciences (WEISS),<br>Ranieri, Veronica; Tavistock and Portman NHS Foundation Trust, Research & Development; University College London, Science & Technology Studies<br>Palmer, D. H.; University of Liverpool<br>Littler, Peter; Freeman Hospital<br>Ghaneh, Pauleh; University of Liverpool<br>Gurusamy, Kurinchi; University College London,<br>Manas, Derek; Newcastle Upon Tyne Hospitals NHS Trust<br>Pizzo, Elena; University College London, Applied Health Research<br>Psarelli, Eftychia; University of Liverpool, Liverpool Clinical Trials Centre<br>Gilmore, Roopinder; Royal Free London NHS Foundation Trust, Oncology; University College London<br>Peddu, Praveen; King's College Hospital NHS Trust, Department of Radiology,<br>Bartlett, David; University Hospitals Birmingham NHS Foundation Trust<br>de Liguori Carino, Nicola; Manchester Royal Infirmary, Regional Hepato-Pancreato-Biliary Unit<br>Davidson, Brian; Royal Free London NHS Foundation Trust, HPB Surgery
**Primary Subject Heading**:	Oncology
Secondary Subject Heading:	Radiology and imaging, Evidence based practice, Surgery
Keywords:	Adult oncology < ONCOLOGY, SURGERY, Pancreatic surgery < SURGERY, Interventional radiology < RADIOLOGY & IMAGING, Clinical trials < THERAPEUTICS

SCHOLARONE™
Manuscripts

Author Declaration

Research Ethics

The trial protocol has received the favourable opinion of the Brent Research Ethics Committee (REC).

Competing interests

We wish to confirm that there are no known conflicts of interest associated with this publication and there has been no significant financial support for this work that could have influenced its outcome.

Consent for publication

Not applicable

Availability of Data and Material

Not applicable

Funding

The LAP-PIE trial is funded by the National Institute for Health Research (NIHR) under its Research for Patient Benefit (RfPB) Programme (PB-PG-1217-20042).

EP is supported by the National Institute for Health Research ARC North Thames. The views expressed in this publication are those of the author(s) and not necessarily those of the National Institute for Health Research or the Department of Health and Social Care.

Authorship

All listed authors meet the ICMJE criteria. We attest that all authors contributed significantly to the creation of this manuscript, each having fulfilled criteria as established by the ICMJE.

We confirm that the manuscript has been read and approved by all named authors.

We confirm that the order of authors listed in the manuscript has been approved by all named authors.

Acknowledgement

This project is funded by the National Institute for Health Research (NIHR) under its Research for Patient Benefit (RfPB) Programme (PB-PG-1217-20042). The views expressed are those of the author(s) and not necessarily those of the NIHR or the Department of Health and Social Care.

Word count: 4517

For peer review only

Title

Treatment of unresectable **L**ocally **A**dvanced **P**ancreatic cancer with **P**ercutaneous **I**rreversible **E**lectroporation (IRE) following initial systemic chemotherapy (LAP-PIE) trial: Study protocol for a feasibility randomised controlled trial.

ZL Rai, V Ranieri, D Palmer, P Littler, P Ghaneh, K Gurusamy, D Manas, E Pizzo, EE Psarelli, Roopinder Gilmore, Praveen Peddu, David C Bartlett, Nico de'Liguori Carino Davidson BR and the UK LAP-PIE study group

Corresponding author: ZL Rai email: Zainab.rai.19@ucl.ac.uk

Author Contributions

ZLR: manuscript preparation. VR: trial management. DP: protocol design, trial management and co-applicant. PL: protocol design, trial management and co-applicant. EEP: statistical analysis plan. KG: major contribution to design and funding application. DM: protocol design, trial management and co-applicant. PG: protocol design, trial management and co-applicant. EP: health economic analysis design. RG: protocol design, site lead, PP: protocol design, site lead, DB: site lead, NLC: site lead. BRD: principal investigator and grant holder, overall trial management, study design and review of manuscript. All authors read and approved the final manuscript.

Keywords

Locally advanced pancreatic cancer, Irreversible electroporation, Feasibility study, Randomised controlled trial, qualitative assessment

Abstract

Background

Approximately 30% of patients with pancreas cancer have unresectable locally advanced disease, which is currently treated with systemic chemotherapy. A new treatment option of Irreversible Electroporation (IRE) has been investigated for these patients since 2005. Cohort studies suggest that IRE confers a survival advantage, but with associated, procedure related complications. Selection bias may account for improved survival and there have been no prospective randomised trials evaluating the harms and benefits of therapy. The aim of this trial is to evaluate the feasibility of a randomised comparison of IRE therapy with chemotherapy versus chemotherapy alone in patients with locally advanced pancreatic cancer (LAPC).

Strengths and limitations of this study

- This trial is the first randomised comparison of IRE therapy with chemotherapy versus chemotherapy alone in patients with LAPC and will aim to evaluate the feasibility of recruitment of LAPC patients across multiple pancreas cancer specialist units in the UK.
- The results will give a clearer indication on the impact, if any, of IRE on LAPC.
- As a feasibility study, it is not powered to show effectiveness however it is likely that the data will help inform a larger multi-centre trial.

- Experience in optimising recruitment, site set up and developing local radiology expertise will be crucial for the success of the subsequent trial which will be powered from data collected during this feasibility study.
- A potential limitation of the study is inadequate recruitment due to competing trials recruiting patients with locally advanced pancreatic cancer.

Methods and analysis

Eligible patients with locally advanced pancreatic cancer who have undergone first line FOLFIRINOX chemotherapy will be randomised to receive either a single session of IRE followed by (if indicated) further chemotherapy or to chemotherapy alone (standard of care). Fifty patients from up to seven specialist pancreas centres in the UK will be recruited over a period of 15 months. Trial follow up will be 12 months. The primary outcome measure is ability to recruit. Secondary objectives include practicality and technical success of treatment, acceptability of treatment to patients and clinicians and safety of treatment. A qualitative study has been incorporated to evaluate the patient and clinician perspective of the LAP-PIE trial. It is likely that the data obtained will guide the structure, the primary outcome measure, the power, and the duration of a subsequent multi-centre RCT aimed at establishing the clinical efficiency and cost effectiveness of pancreas IRE therapy.

Ethics and Dissemination

The protocol has received a favourable opinion by London-Brent Research Ethics Committee reference number 21/LO/0077.

Results will be analysed following completion of trial recruitment and follow up. Results will be presented to international conferences with an interest in oncology, hepato-pancreatico-biliary surgery and interventional radiology and be published in a peer-reviewed journal.

Introduction

Pancreas cancer and LAPC

Pancreatic cancer (PC) is one of the deadliest cancers worldwide. It has a five-year survival rate of less than 6% and an increasing incidence (1). The low survival rate is mainly due to late diagnosis, as PC often remains asymptomatic until it becomes advanced. Surgery (pancreatic resection) is the only potentially curative

treatment. However, only 10 to 20% of patients are eligible for surgery (2). Approximately 30% of all patients have locally advanced pancreatic cancer (LAPC) at diagnosis and are not considered for surgical resection (3).

Chemotherapy for LAPC

Systemic chemotherapy is the standard treatment for unresectable pancreatic cancer (4). Despite advances in drug therapy for pancreas cancer over the last 20 years the overall survival (OS) and progression free survival (PFS) of these patients has not significantly improved (5). Furthermore, a meta-analysis reported that chemoradiation is not superior to chemotherapy alone in patients with LAPC, with comparable overall survival and higher rates of grade 3–4 adverse events (6).

In the UK NICE guidelines, published in 2018 recommend systemic combination chemotherapy for both locally unresectable and metastatic pancreatic cancer (7). NICE recommends first-line combination chemotherapy with 5-FluoroUracil, Leucovorin, Irinotecan and Oxaliplatin (FOLFIRINOX) in fit patients (Eastern Cooperative Oncology Group (ECOG) performance status of 0-1). In patients with co-morbidities and restricted activities, or those unable to tolerate this regime, NICE recommends gemcitabine alone or gemcitabine based therapy (6). Although FOLFIRINOX shows the best objective response in pancreatic cancer (8, 9) it is associated with more frequent and severe side effects, including neutropenia, diarrhoea and peripheral neuropathy (10).

Repeat staging after first line chemotherapy and possibility of resection

Staging is repeated following first line chemotherapy for LAPC and imaging is reviewed once again regarding suitability for resection. In patients with locally advanced disease undergoing first line treatment with FOLFIRINOX, 25.9%

For peer review only - http://bmjopen.bmj.com/site/about/guidelines.xhtml

underwent resection, with an R0 resection rate of 78.4%, however the long-term outcomes for these patients is not reported in the studies, and there is still a possibility of recurrence albeit at later date. (11).

IRE for LAPC

Irreversible electroporation is a novel ablative method for treating solid cancers (12). IRE treatment destroys cancer cells using electricity. It can be delivered via an open approach involving a laparotomy and placement of probes around the cancer or via a percutaneous approach which requires image guidance, via CT or ultrasound. Both methods require the patient to be under general anaesthesia with neuromuscular blockade. A single study using propensity score matching reported a median OS of 21.6 months in patients with LAPC treated with induction chemotherapy followed by IRE (13). A systematic review on pancreas IRE therapy from 2019 reported an OS from diagnosis or treatment of up to 27 months and a morbidity rate of 30% (14).

First Line, Up-front Chemotherapy

The timing of chemotherapy in relation to IRE therapy may influence outcome. Studies where IRE has been delivered before chemotherapy have reported only modest increases in median OS (15, 16) whereas a systematic review evaluating the impact of IRE after induction treatment reported an overall survival of 27 months (14). This may be because chemotherapy responders who have better outcomes are being selected to undergo IRE or alternatively it may suggest that chemotherapy modifies the tumour microenvironment making it more sensitive to IRE.

Second line chemotherapy for LAPC

For peer review only - http://bmjopen.bmj.com/site/about/guidelines.xhtml

NICE recommends that patients who have previously undergone FOLFIRINOX therapy as first line should be offered Gemcitabine based second-line chemotherapy if their cancer has progressed on FOLFIRIONOX (17).

Locally advanced and borderline unresectable LAPC

The definition of borderline resectable disease is controversial (18), whereas the definition of unresectable locally advanced disease has clear consensus, namely the involvement of the coeliac artery or encasement of the superior mesenteric artery of more that 180º and or the involvement of the superior mesenteric vein or portal vein with no distant metastasis (19). Therefore, the LAP-PIE trial will only involve patients with locally advanced disease fulfilling published criteria.

Complications of pancreas IRE therapy

Reported complications following pancreas IRE include pancreatic fistula, venous and arterial thrombosis, arterial pseudoaneurysm and pancreatic abscess (14). The mean morbidity rate in the studies examined is 34%, but can reach up to 62% (20) The average rate of severe complications (defined as greater than Grade III on the Clavien-Dindo scale) following IRE is 8% but can be as high as 44% (21). The average procedure related mortality rate in these studies is 3% (12).

There are significant variations in the percentage and severity of complications reported. This difference may be due to the case selection, whether IRE is carried out during surgery (via an open or laparoscopic approach) or with image-guidance in the Interventional Radiology (IR) suite, the treatment algorithm, the completeness of reporting from treatment centres or the experience of the radiologist and/or centre. Only two studies specifically mention the experience of clinicians delivering IRE (16,

For peer review only

22) and currently, there is limited published data available exploring the number of IRE procedures performed and the relationship with outcomes (23).

IRE is a palliative option and therefore its impact on patient's quality of life (QoL) is an essential consideration. A single-centre prospective study evaluating the QoL pre and post IRE in patients with LAPC demonstrated that IRE had no adverse effect on QoL in the short-term however, concomitant treatment with chemotherapy after IRE as well as other inter-related factors may confound the results (24). We were unable to find any studies comparing QoL changes following IRE to a control group. The published data to date, regarding the improvement in QoL in patients with LAPC undergoing IRE is weak and needs further exploration.

The current information available in the literature regarding the use of IRE in the treatment of LAPC is from retrospective and prospective cohort studies. A prospective randomised controlled trial is required to demonstrate efficiency and cost effectiveness of pancreas IRE therapy. The LAP-PIE trial is a feasibility trial to determine whether patients would be willing to have pancreas IRE treatment, whether they would accept randomisation to IRE or standard of care chemotherapy, whether they would comply with treatment and study data collection and to collect preliminary data on efficacy end points and indicative costs related to health resources, with which to design a subsequent trial on treatment efficiency and cost effectiveness.

Methods and analysis

Study design and setting

The LAP-PIE trial is a UK multi-centre feasibility study that will recruit 50 patients with LAPC in whom the disease has remained localised and unresectable

For peer review only

following FOLFIRINOX between 3- to 6-months of chemotherapy. All participants will be offered palliative chemotherapy according to standard centre protocols and half will also be offered one IRE procedure. If new agents for second line therapy are approved by NICE prior to or during the LAP-PIE trial these can be offered in the second line chemotherapy arm and following IRE therapy if considered appropriate by the treating oncologist. It is an open-label study. Patient enrolment is expected to last 15 months assuming a recruitment rate of 0.8 patients per site per month across 7 UK centres and an opening site rate of 0.5 per month. The trial will be conducted in specialist pancreas cancer centres. IRE will be delivered across all trial centres. Interventional radiologists performing pancreas IRE in the trial will have performed a minimum of 5 previous pancreas or liver IRE ablations prior to administrating IRE to participants enrolled in the trial. Additional technical support will be available from manufacturers of the equipment, AngioDynamics ®. Figure 1 summarises the trial design and Table 1 demonstrates the schedule of enrolment, interventions and assessments.

Participants

Adults (≥18 years) with LAPC following first line chemotherapy with FOLFIRINOX will be eligible. Only patients who have completed FOLFIRINOX as their first line chemotherapy will be eligible. Patients who have received FOLFIRINOX as second line therapy prior to inclusion will not be eligible. Following FOLFIRINOX chemotherapy patients will be re-staged with CT chest, abdomen and pelvis (CAP) +/- an FDG Positron Emission Tomography (PET) scan, depending on local site protocols. The imaging will be reviewed in a specialist multi-disciplinary team (sMDT) meeting.

For peer review only - http://bmjopen.bmj.com/site/about/guidelines.xhtml

Patients with borderline resectable disease will be excluded. The definition for this is summarised in Table 2. Other exclusion criteria include untreated biliary or gastric outlet obstruction, coagulopathy, chronic kidney disease (CKD) stage ≥ 3 (the criteria for CKD stage 3 is an estimated glomerular filtration rate of between 30 and 59) (25), indwelling electrical devices, abdominal varices precluding safe access to the pancreas and significant co-morbidities that would contraindicate general anaesthesia and neuromuscular blockade. A contraindication, as per manufacturers guidance, is epilepsy as the electrical discharges from IRE treatment may in theory result in seizure activity through pulsatile brain stimulation. Although the overall impact of IRE on seizure activity and neurological conditions remains unclear (26), in the interest of robust patient safety, patients with neurological conditions will be excluded. The full inclusion and exclusion criteria are summarised in Figure 2.

Figure 1: Trial flow diagram

sMDT; specialist multidisciplinary team, IRE; irreversible electroporation, SoC; standard of care, CTCAP; computed tomography chest abdomen pelvis, QoL quality of life, PFS; progression free survival, OS; overall survival.

Figure 2: Inclusion and exclusion criteria of the LAP-PIE trial

LAPC: Locally Advanced Pancreatic Cancer, FOLFIRINOX; 5-FluoroUracil, Leucovorin, Irinotecan and Oxaliplatin, sMDT; specialist multidisciplinary team, IRE; irreversible electroporation, IR; interventional radiologist, WHO; world health organisation, CKD; chronic kidney disease, Hb; haemoglobin

For peer review only - http://bmjopen.bmj.com/site/about/guidelines.xhtml

Table 1. Schedule of enrolment, interventions, and assessments of the LAP-PIE trial.

	**Study Period**									
	**Enrolment**	**Allocation**	**Post-Allocation Follow-up Time Points**							**Close-out**
**TIMEPOINT**	***-t₁***	**0**	***Pre-IRE***	***6 hours post -IRE***	***6 days post -IRE***	***3 months***	***6 months***	***9 months***	***12 months***	
**ENROLMENT:**										
**Eligibility screen**	X									
**PIS given**	X									
Systemic treatment with FOLFIRINOX	X									
sMDT	X									
Informed consent	X									
**Allocation**		X								
**INTERVENTIONS:**										
IRE (intervention)			—	—	—	—	—	—	—	
Systemic chemotherapy (SoC)						—	—	—	—	
**ASSESSMENTS:**										
CT CAP	X					X	X	X	X	
Blood tests	X		X	X	X	X	X	X	X	X
HRQoL						X	X	X	X	X
Complications						X	X	X	X	X

PIS patient information sheet, sMDT specialist multidisciplinary team, IRE irreversible electroporation, SoC standard of care, CT CAP computed tomography chest abdomen pelvis, HRQoL health related quality of life.

For peer review only

Table 2: Definition of borderline resectable pancreatic cancer (18)

	**Borderline Resectable**	**Unresectable**
**Celiac artery**	Tumour without encasement or abutment	Tumour contact > 180º
**Superior Mesenteric artery**	Tumour abutment </= 180º	Tumour contact > 180º
**Superior Mesenteric vein (SMV)/Portal vein (PV)**	Tumour abutment of SMV/PV > 180º or abutting</= 180º with irregularity of the vein ± thrombosis with anatomical structures that still permit safe and complete resection with vein reconstruction	SMV/PV unreconstructible secondary to tumour involvement or occlusion
**Common hepatic artery**	Reconstructible short segment abutment	Unreconstructible interface between tumour mass and vessel

Adapted from Callery MP, Chang KJ, Fishman EK et al. Pre-treatment assessment of resectable and borderline resectable cancer: expert consensus statement. Ann Surg Oncol 2009; 16:1727-33.

Randomisation

Following confirmation of eligibility randomisation will be performed by the Liverpool Clinical Trials Centre (LCTC) using a web-based randomisation procedure. The LCTC will inform local centres of the treatment arm and the local LAP-PIE Clinical Trials Nurse will inform the patient and make arrangements for clinical review and the date for the chosen therapy.

Intervention

Pancreas IRE

Patients will be invited for an anaesthetic pre-assessment to ensure they are fit enough to undergo IRE under general anaesthesia prior to being booked for hospital admission. Patients will be admitted to a ward or radiology admissions unit under joint clinical care of a pancreas interventional radiologist and one of the other site leads (Surgery, Oncology).

The CT CAP performed on completion of FOLFIRINOX will be used by the interventional radiologist to plan IRE therapy. Under general anaesthesia IRE will be delivered percutaneously using CT guidance. Timing of the procedure will be co-

For peer review only - http://bmjopen.bmj.com/site/about/guidelines.xhtml

ordinated with the treating oncologist to ensure bone marrow recovery following FOLFIRINOX chemotherapy. A broad-spectrum antibiotic, based on individual centres first line prophylaxis for GI infection, will be given prior to the procedure and 6 hours and 12 hours following IRE.

The IRE procedure with be standardised to use between 2-6 IRE needles depending on the size, shape and relationship of critical structures to the tumour, with a maximum inter-electrode distance of 2cm. A treatment margin of at least 0.5cm will be maintained. The needle placement prior to treatment will be recorded on CT.

IRE therapy will be delivered using ECG synchronisation to ensure the pulse is delivered during diastole. The length of the electrical pulse and amperage will be decided by the treating interventional radiologist and will be between 70-100 microseconds with an amperage of between 20-40 AMPs. The treating interventional radiologist will have relevant expertise in IRE procedures with a minimum of 5 IRE procedures on liver or pancreas performed previously.

Following IRE therapy, full blood count, liver function tests, coagulation, renal function, and amylase (or lipase) will be monitored and patients will be admitted for a minimum of 24 hours to ensure adequate pain management and to monitor and manage any immediate complications. Participants will be discharged with pain relief as required and DVT prophylaxis for 4 weeks.

The intra-operative imaging will be reviewed by the lead radiologist at each site and if the entire tumour has been treated based on these images, the procedure will be deemed as successful. The technical success will be confirmed by central review. Contrast CT follow up scans will be assessed for evidence of tumour growth as indicated by mass enlargement or increased vascularity. In the absence of tumour growth, the procedure will be considered successful at follow up. At the end of the

For peer review only

For peer review only - http://bmjopen.bmj.com/site/about/guidelines.xhtml

follow up period, serial imaging will be analysed, and the tumour staged. Local disease control will be evaluated according to the Response Evaluation Criteria in Solid Tumours (RECIST). This will be performed by the radiology leads at each site.

Following IRE, patients in this arm will be offered second line chemotherapy, which is the current standard of care. The drug choice and timing of chemotherapy will be decided by treating oncologists and will adhere to NICE guidelines.

Controls (Second line systemic chemotherapy)

Patients randomised to receive further chemotherapy (chemotherapy alone) will commence treatment within 6 weeks after sMDT or 4 weeks after randomisation, whichever is sooner. The second line chemotherapy regime will be selected at the discretion of the treating oncologist and will adhere to NICE guidelines and local protocols.

Outcomes

The primary objective of the LAP-PIE trial is to ascertain feasibility of such a trial concept. In particular, the ability to identify and recruit LAPC patients at the selected sites to the LAP-PIE trial will be evaluated. The primary outcome consists of recruitment rate, number of screening failures, number of patients completing study pathway as per protocol and trial withdrawal rate.

The secondary objectives of the LAP-PIE trial aim to assess the practicality and technical success rate of pancreas IRE treatment. Technical success will be determined by the interventional radiologist performing the procedure. The procedure will be deemed to be technically successful if the entire tumour has been treated based on imaging performed during the intervention. Completion and complication rates of IRE procedures will be evaluated. The proportion of patients in whom percutaneous IRE was successful, defined as no local growth on follow up, will be determined as will

For peer review only

the proportion of patients who became eligible for surgery following IRE and the R0/R1 resection rates in these participants. Acceptability of pancreas IRE therapy to patients and their clinicians, rate of withdrawal from the study and outcome measures including overall survival (measured from randomisation), progression free survival and quality of life will be evaluated. Finally, health related costs, including indicative costs related to health resource use in both arms, social costs of attending IRE treatment such as cost of travel to and from a tertiary centre, time off work and social support costs will be evaluated.

Follow up

All patients will be clinically reviewed every 3 months during which a CT CAP will be performed. Additional imaging can be performed if clinically indicated. Other investigations will include biochemistry and haematology, CA19-9 levels, CRP, quality of life as measured by a validated questionnaire (EQ5D-5L) (27) and a clinical review of any adverse events experienced. This information will be recorded in an electronic case report form (eCRF).

Sample size calculation

Formal sample size calculations are not appropriate for a feasibility study. Sample sizes between 24 and 50 have been recommended to estimate the standard deviation required for a sample size calculation to allow for the design of a subsequent randomised controlled trial aimed at evaluating the cost effectiveness of IRE in patients with LAPC (28, 29). A convenient sample size of 50 patients, randomised equally to the two groups, has been selected.

For peer review only

Patient and Public Involvement (PPI)

PPI representatives have been involved in designing the trial ensuring that patient interests are central to the study. The design of the trial was presented to the Pancreas Cancer Action Day which has a large representation from patients and carers and received extremely positive feedback. Two patient representatives will serve on the Trial management group (TMG) and will be involved in informing, monitoring, and helping to interpret the findings from the study. Both patient representatives were involved in the trial design and in developing the trial protocol and patient information sheets. As the trial progresses, they will help address any patient related concerns and will have input into the interpretation of the trial outcomes and how this will be communicated to the patient user groups. They will then be involved, along with other patient advocates, the trial management team and other key invited participants in the discussion of the trial findings at a focus group meeting and the preparation of the protocol for a subsequent trial addressing efficacy and cost effectiveness of IRE therapy in pancreas cancer. Once the data analysis and interpretation has been completed they will contribute to the dissemination of the trial outcome information to patients support groups via social media.

Assessment of feasibility

One aspect of the feasibility evaluation of this trial is to evaluate the ability to recruit LAPC patients into a trial of this nature. The average number of patients with unresectable locally advanced pancreatic cancer within UK specialist pancreas units is 60 patients per centre per year (Royal Free Hospital London audit data 2017, unpublished). Staging following FOLFIRINOX chemotherapy will demonstrate localised but unresectable disease in approximately 40 out of 60 patients (65%) (12). These patients will be eligible for the LAP-PIE trial. We anticipate recruiting 20% of

For peer review only

potential participants to the LAP-PIE trial which equates to 8 per centre per year. Recruitment within 5 centres would allow 40 UK patients per year. A recruitment time of 15 months would allow 50 patients to be recruited.

Assessment of Complications

Adverse Events (AE) for the Standard of Care Chemotherapy will be defined using the Common Terminology Criteria for Adverse Events (CTCAE) Version 4.0 (30). AEs for IRE will be defined using the Clavien Dindo classification of surgical complications (31). Complications greater than grade 3 on the CTCAE scale and greater than grade III on the Clavien Dindo scale will be reported.

A Serious Adverse Event (SAE) for patients randomised to receive Standard of Care Chemotherapy only will be compared against the reference safety information (RSI) within the current summary of product characteristics (SmPC) for that drug. The RSI will be taken from the latest SmPC available for the drug on https://www.medicines.org.uk/emc. The RSI for IRE will be from the Nanoknife system users' manual.

Suspected Unexpected Serious Adverse Reaction (SUSAR) will be defined as life threatening, serious and non-recognised complications. 2 or more reports of SUSARs related to IRE therapy will result in the cessation of trial activity in all centres to allow for a thorough investigation into the safety of IRE. Any premature halt in the trial will be reported to the Medicines and Healthcare products Regulatory Agency (MHRA) and the Regional Ethics Committee (REC).

Qualitative Study in LAP-PIE trial

A qualitative study will be carried out to understand patients' and clinicians' perspectives and experiences of the procedure and the design of the trial. All patients

For peer review only

approached to participate in the trial will be invited to participate. A purposive sample of healthcare professionals involved in the trial will also be invited. Trial nurses, local R&D (research and development) personnel and Network CRN (clinical research network) managers will be included. This information will be gathered via interviews by an experienced qualitative researcher.

Interviews will be transcribed verbatim and managed using computer-assisted qualitative data analysis software (iNVivo) (32). Emerging data will be used to modify trial information and protocol. This may include the development of training materials for staff and changes to participant information sheets.

Translational Study

A translational sub study will evaluate prognostic markers for outcome. Imaging prior to and during IRE therapy will be correlated with outcome as will the immune response to IRE therapy by assessing circulating immune responses (including absolute lymphocyte count, eosinophils, neutrophil: leucocyte ratio, Tregs and IFNγ) at baseline, 6 hours and 6 days post therapy.

Progress to full trial

The criteria to progress to a subsequent full trial will be determined quantitatively as (A) the ability to recruit 50 patients to the study across the study sites (B) a consent rate of 20+/-11%, (C) overall technical success rate of 50+/-14% and (D) a loss to follow-up of 10+/-8%.

For peer review only

Ethics

The trial will abide by the principles of the World Medical Association Declaration of Helsinki and has approval from London Brent Research Ethics Committee (REC), reference number 21/LO/0077. The trial has been registered on ISRTCN (International Standard Randomised Controlled Trial Number) https://doi.org/10.1186/ISRCTN14986389

Publication and Dissemination

Results from different centres will be analysed and published together following completion of the recruitment and follow up period. This will be presented to an international conference with an interest in hepato-pancreaticobiliary surgery, oncology and interventional radiology and published in a peer- reviewed journal.

Discussion

Trial justification

Local ablative therapy using IRE in patients with LAPC has been shown to be beneficial in prospective and retrospective studies (median survival 27 months) (14), however the increase in survival reported may be due to patient selection for IRE rather than the effect of IRE (selection bias). A systematic review of IRE therapy in LAPC included retrospective and prospective studies but no randomised controlled trials (RCT) (14, 33). Moreover, the heterogeneity among individual trial participants including varying concomitant treatments prevented combination of data sets making it difficult to establish the true impact of IRE. Despite this, the systematic review is a useful aid in summarising findings including complication rates following IRE and

For peer review only

highlighting the current knowledge gaps such as the relationship between number of procedures performed and outcome and the impact of IRE on quality of life.

A study using propensity score matching (PSM) reported a median OS of LAPC patients undergoing IRE of 21 months (13), similar to that reported by the systematic review (27 months) (14). Such a study may effectively adjust for observed variables between cohorts and can contribute to a more precise estimation of treatment response. However, selection bias cannot be completely eliminated because PSM does not adjust for unobserved differences between groups. In order to address selection bias, a well-designed randomised control trial is required.

Before IRE can be justified by NICE to be incorporated into routine clinical practice, a randomised control trial is needed. Whilst a larger trial is required to investigate IRE treatment in locally advanced pancreatic cancer, patients and clinicians may not be willing to enter the trial and therefore currently a large RCT is justified but not feasible.

Importance of doing a feasibility study

Clinical trials comparing different treatment types have high rates of failure (34). This is particularly true where there has been a lack of feasibility testing and qualitative evaluation of the study design (26,27). Furthermore, if a feasibility study demonstrates that a full trial would be unlikely to meet the required patient recruitment rate, intervention acceptability and other feasibility outcomes then the potential wasted cost of a larger clinical trial is avoided making feasibility trials an important first step (35). A feasibility trial will enable a realistic assessment of the capability of registered sites in patient enrolment and will compare IRE to the current standard of care. The qualitative study will explore the patient evaluation of information provided, the quality and clarity of this information, the individual providing the information, the clinical equipoise,

For peer review only

support and attitude of other clinicians and general practitioners and the views and experiences of patients' family and friends. The feasibility trial will provide information about challenges that may be encountered during enrolment and these factors can be mitigated for if a larger RCT is justified and subsequently planned.

Exclusion of borderline resectable disease

Patients with borderline resectable disease have been excluded from the LAP-PIE trial. The exact definition of borderline resectable pancreatic disease (BRPD) varies, but broadly it is characterised as tumour abutting or involving vascular structures (18). A number of studies have suggested that neoadjuvant chemotherapy or chemoradiotherapy may downsize the tumour allowing resection and preventing local recurrence (36-38). The LAP-PIE trial will exclude patients with BRPD because of the variability in radiological criteria used for BRPD and to avoid patients being recruited to the trial who would be considered in some pancreas units for surgical intervention.

Choice of FOLFIRINOX as first line

Previous studies have reported no significant improvement in survival when IRE is used as a first line treatment for unresectable LAPC (15, 16). Furthermore, systemic chemotherapy has previously been shown to downgrade a proportion of locally advanced unresectable cancer into resectable disease and therefore delivering IRE first line may result in an unnecessary, invasive procedure in a group of patients whose tumours may be downgraded and who would go on to have potentially curative resectional surgery.

FOLFIRINOX was specifically selected as the regime of choice because it has been shown to result in the greatest number of patients downgraded to resectable

For peer review only - http://bmjopen.bmj.com/site/about/guidelines.xhtml

disease (10). Therefore, the patients recruited into the LAP-PIE trial will be those with truly unresectable disease in whom the impact of IRE can be evaluated.

Translational studies

The translational study will determine the radiological and immune response of patients undergoing IRE and determine its biological significance with emphasis on overall survival and disease response. This data will provide valuable information about the type of patient that is likely to benefit the most from IRE and therefore form the basis of a management design that is tailor made to individual patients.

Mandated imaging in the LAP-PIE trial is a CT scan and this will provide information about contrast enhancement features. The images will be reviewed and information about tumour characteristics such as size, histology and grade will be collected. The relationship between treatment parameters and imaging findings during the IRE procedure, as well as pre-treatment PET scans, will be analysed. The data generated will be used to develop a mathematical model to predict IRE responses and help understand the significance of radiological changes and their biological significance. Blood samples will be taken from patients enrolled in the study and analysed for peripheral immune response markers. This will include absolute lymphocyte count and eosinophils, neutrophil: leucocyte ratio, Tregs and IFNγ. Previous studies have demonstrated a detectable peripheral immune response in patients following IRE (39, 40). Analysing the immune response of patients undergoing IRE will provide information about whether a peripheral immune response can be determined by simple biochemical tests in these patients and if this can predict treatment efficacy. This information may be used in the future to predict treatment response and guide future patient selection.

For peer review only

The qualitative sub-study will provide the opportunity to collate and analyse the experiences of both patients and clinicians in order to understand the perspectives and experiences of the procedure and trial providing useful information on the barriers and facilitators experienced by both groups.

Preliminary studies have shown that IRE in addition to chemotherapy may provide survival benefit in patients with locally advanced pancreatic cancer (14-16) (21-23) (33). However, this may be due to patient selection for IRE. There are no randomised controlled trials addressing the issue. The LAP-PIE trial is the first UK-based RCT of pancreas IRE in LAPC patients. The trial will assess feasibility and safety of delivering this intervention and if successful, will progress to a subsequent large scale RCT trial in assessing the efficacy and cost-effectiveness of IRE therapy in pancreatic cancer. This research is required to investigate whether IRE improves survival, health-related quality of life, or both.

For peer review only

References

1. Are C, Chowdhury S, Ahmad H, Ravipati A, Song T, Shrikandhe S, et al. Predictive global trends in the incidence and mortality of pancreatic cancer based on geographic location, socio-economic status, and demographic shift. J Surg Oncol. 2016;114(6):736-42.
2. Ghaneh P, Costello E, Neoptolemos JP. Biology and management of pancreatic cancer. Postgrad Med J. 2008;84(995):478-97.
3. Balaban EP, Mangu PB, Khorana AA, Shah MA, Mukherjee S, Crane CH, et al. Locally Advanced, Unresectable Pancreatic Cancer: American Society of Clinical Oncology Clinical Practice Guideline. J Clin Oncol. 2016;34(22):2654-68.
4. Ghosn M, Ibrahim T, Assi T, El Rassy E, Kourie HR, Kattan J. Dilemma of first line regimens in metastatic pancreatic adenocarcinoma. World J Gastroenterol. 2016;22(46):10124-30.
5. Rahib L, Fleshman JM, Matrisian LM, Berlin JD. Evaluation of Pancreatic Cancer Clinical Trials and Benchmarks for Clinically Meaningful Future Trials: A Systematic Review. JAMA Oncol. 2016;2(9):1209-16.
6. Wang C, Liu X, Wang X, Wang Y, Cha N. Effects of chemoradiotherapy and chemotherapy on survival of patients with locally advanced pancreatic cancer: A meta-analysis of randomized controlled trials. Medicine (Baltimore). 2018;97(36):e12260.
7. Excellence NIfHaC. Overview | Pancreatic cancer in adults: diagnosis and management | Guidance | NICE: NICE; 2020 [Available from: https://www.nice.org.uk/guidance/ng85.
8. Pavillard V, Formento P, Rostagno P, Formento JL, Fischel JL, Francoual M, et al. Combination of irinotecan (CPT11) and 5-fluorouracil with an analysis of cellular determinants of drug activity. Biochem Pharmacol. 1998;56(10):1315-22.
9. Ducreux M, Mitry E, Ould-Kaci M, Boige V, Seitz JF, Bugat R, et al. Randomized phase II study evaluating oxaliplatin alone, oxaliplatin combined with infusional 5-FU, and infusional 5-FU alone in advanced pancreatic carcinoma patients. Ann Oncol. 2004;15(3):467-73.
10. Suker M, Beumer BR, Sadot E, Marthey L, Faris JE, Mellon EA, et al. FOLFIRINOX for locally advanced pancreatic cancer: a systematic review and patient-level meta-analysis. Lancet Oncol. 2016;17(6):801-10.
11. Hajatdoost L, Sedaghat K, Walker EJ, Thomas J, Kosari S. Chemotherapy in Pancreatic Cancer: A Systematic Review. Medicina (Kaunas). 542018.
12. Hurt CN, Falk S, Crosby T, McDonald A, Ray R, Joseph G, et al. Long-term results and recurrence patterns from SCALOP: a phase II randomised trial of gemcitabine- or capecitabine-based chemoradiation for locally advanced pancreatic cancer. Br J Cancer. 2017;116(10):1264-70.
13. He C, Wang J, Sun S, Zhang Y, Lin X, Lao X, et al. Irreversible electroporation versus radiotherapy after induction chemotherapy on survival in patients with locally advanced pancreatic cancer: a propensity score analysis. BMC Cancer. 192019.
14. Lafranceschina S, Brunetti O, Delvecchio A, Conticchio M, Ammendola M, Currò G, et al. Systematic Review of Irreversible Electroporation Role in Management of Locally Advanced Pancreatic Cancer. Cancers (Basel). 112019.

For peer review only

15. Mansson C, Brahmstaedt R, Nygren P, Nilsson A, Urdzik J, Karlson BM. Percutaneous Irreversible Electroporation as First-line Treatment of Locally Advanced Pancreatic Cancer. Anticancer Res. 2019;39(5):2509-12.
16. Belfiore MP, Ronza FM, Romano F, Ianniello GP, De Lucia G, Gallo C, et al. Percutaneous CT-guided irreversible electroporation followed by chemotherapy as a novel neoadjuvant protocol in locally advanced pancreatic cancer: Our preliminary experience. Int J Surg. 2015;21 Suppl 1:S34-9.
17. Excellence NIfHaC. Recommendations | Pancreatic cancer in adults: diagnosis and management | Guidance | NICE: NICE; 2020 [Available from: https://www.nice.org.uk/guidance/ng85/chapter/Recommendations#managing-resectable-and-borderline-resectable-pancreatic-cancer.
18. Callery MP, Chang KJ, Fishman EK, Talamonti MS, William Traverso L, Linehan DC. Pretreatment assessment of resectable and borderline resectable pancreatic cancer: expert consensus statement. Ann Surg Oncol. 2009;16(7):1727-33.
19. Network NCC. Pancreatic Adenocarcinoma, Version 2.2017, NCCN Clinical Practice Guidelines in Oncology - NCCN-2017-pancreatic-cancer.pdf: NCCN; 2017 [Available from: http://clubedopancreas.pt/wp-content/uploads/2018/06/NCCN-2017-pancreatic-cancer.pdf.
20. Narayanan G, Hosein PJ, Beulaygue IC, Froud T, Scheffer HJ, Venkat SR, et al. Percutaneous Image-Guided Irreversible Electroporation for the Treatment of Unresectable, Locally Advanced Pancreatic Adenocarcinoma. J Vasc Interv Radiol. 2017;28(3):342-8.
21. Kluger MD, Epelboym I, Schrope BA, Mahendraraj K, Hecht EM, Susman J, et al. Single-Institution Experience with Irreversible Electroporation for T4 Pancreatic Cancer: First 50 Patients. Ann Surg Oncol. 2016;23(5):1736-43.
22. Liu S, Qin Z, Xu J, Zeng J, Chen J, Niu L, et al. Irreversible electroporation combined with chemotherapy for unresectable pancreatic carcinoma: a prospective cohort study. Onco Targets Ther. 2019;12:1341-50.
23. Philips P, Hays D, Martin RC. Irreversible electroporation ablation (IRE) of unresectable soft tissue tumors: learning curve evaluation in the first 150 patients treated. PloS one. 2013;8(11).
24. Field W, Rostas JW, Martin RCG. Quality of life assessment for patients undergoing irreversible electroporation (IRE) for treatment of locally advanced pancreatic cancer (LAPC). Am J Surg. 2019;218(3):571-8.
25. Outcomes KDIG. KDIGO 2012 Clinical Practice Guidelines for Evaluation and Management of Chronic Kidney Disease 2013 [Available from: https://kdigo.org/wp-content/uploads/2017/02/KDIGO_2012_CKD_GL.pdf.
26. Goddard GV. Development of epileptic seizures through brain stimulation at low intensity. Nature. 1967;214(5092).
27. Group E. EQ-5D-5L 2009 [Available from: https://euroqol.org/eq-5d-instruments/eq-5d-5l-about/.
28. Julious SA. Sample size of 12 per group rule of thumb for a pilot study. Pharmaceutical Statistics. 2020;4(4):287-91.
29. Sim J, Lewis M. The size of a pilot study for a clinical trial should be calculated in relation to considerations of precision and efficiency. J Clin Epidemiol. 2012;65(3):301-8.
30. Common Terminology Criteria for Adverse Events (CTCAE) - CTCAE_4.03_2010-06-14_QuickReference_5x7.pdf 2020 [Available from: https://www.ncbi.nlm.nih.gov/pubmed/.

For peer review only - http://bmjopen.bmj.com/site/about/guidelines.xhtml

31. Dindo D, Demartines N, Clavien PA. Classification of surgical complications: a new proposal with evaluation in a cohort of 6336 patients and results of a survey. Ann Surg. 2004;240(2):205-13.
32. NVIVO. Qualitative Data Analysis Software | NVivo 2020 [Available from: https://www.qsrinternational.com/nvivo-qualitative-data-analysis-software/home.
33. Rai ZL, Feakins R, Pallett LJ, Manas D, BR D. Irreversible Electroporation (IRE) in Locally Advanced Pancreatic Cancer: A Review of Current Clinical Outcomes, Mechanism of Action and Opportunities for Synergistic Therapy. Journal of clinical medicine. 2021;10(8).
34. Fogel DB. Factors associated with clinical trials that fail and opportunities for improving the likelihood of success: A review. Contemp Clin Trials Commun. 2018;11:156-64.
35. Morgan B, Hejdenberg J, Hinrichs-Krapels S, Armstrong D. Do feasibility studies contribute to, or avoid, waste in research? PLoS One. 2018;13(4):e0195951.
36. Pisters PW, Abbruzzese JL, Janjan NA, Cleary KR, Charnsangavej C, Goswitz MS, et al. Rapid-fractionation preoperative chemoradiation, pancreaticoduodenectomy, and intraoperative radiation therapy for resectable pancreatic adenocarcinoma. J Clin Oncol. 1998;16(12):3843-50.
37. Yeung RS, Weese JL, Hoffman JP, Solin LJ, Paul AR, Engstrom PF, et al. Neoadjuvant chemoradiation in pancreatic and duodenal carcinoma. A Phase II Study. Cancer. 1993;72(7):2124-33.
38. Evans DB, Rich TA, Byrd DR, Cleary KR, Connelly JH, Levin B, et al. Preoperative chemoradiation and pancreaticoduodenectomy for adenocarcinoma of the pancreas. Arch Surg. 1992;127(11):1335-9.
39. He C, Wang J, Sun S, Zhang Y, Li S. Immunomodulatory Effect after Irreversible Electroporation in Patients with Locally Advanced Pancreatic Cancer. J Oncol. 2019;2019:9346017.
40. Pandit H, Hong YK, Li Y, Rostas J, Pulliam Z, Li SP, et al. Evaluating the Regulatory Immunomodulation Effect of Irreversible Electroporation (IRE) in Pancreatic Adenocarcinoma. Ann Surg Oncol. 2019;26(3):800-6.

For peer review only

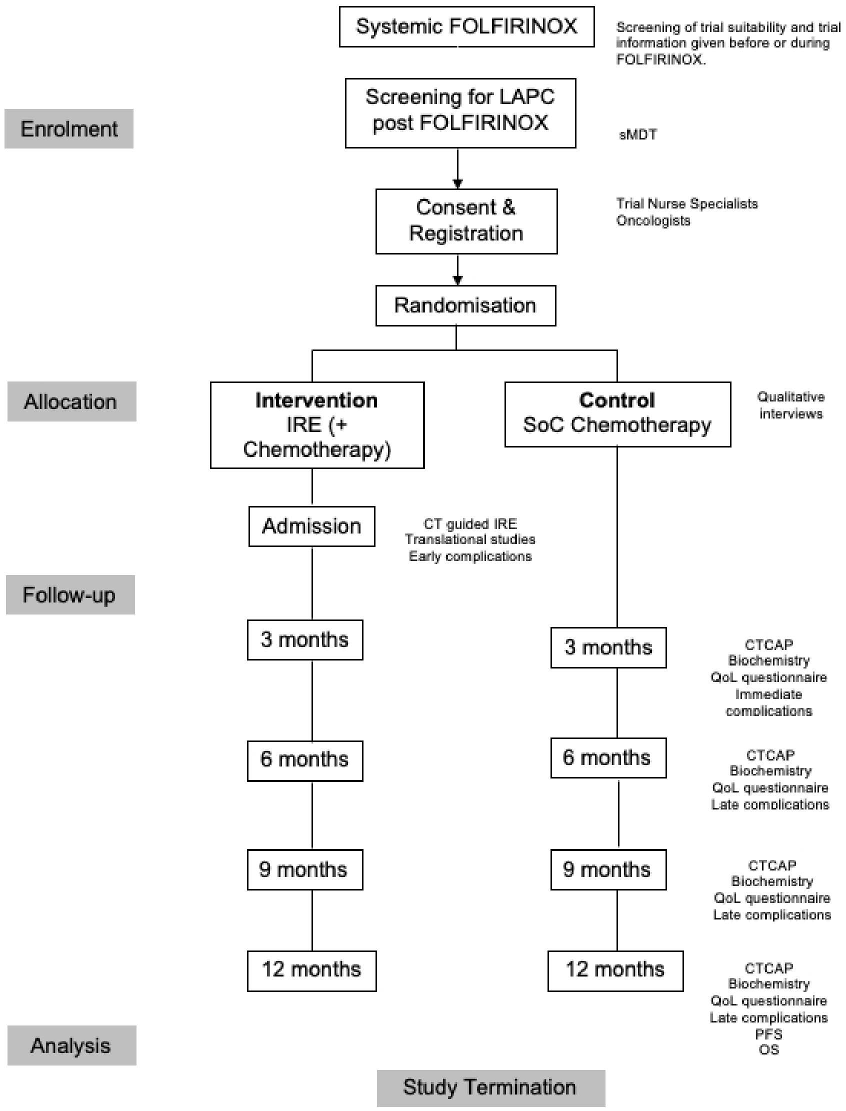

Figure 1: Trial flow diagram

230x256mm (72 x 72 DPI)

Inclusion criteria

- Tissue confirmation of adenocarcinoma
- Successful completion of FOLFIRINOX
- Cancer deemed unresectable at sMDT review
- Ability to provide informed consent
- ≥ 18 years of age
- Considered amenable to IRE by pancreas IR
- WHO Performance status 0 /1.
- Maximum cancer diameter of 3.5 cm at time of IRE treatment.
- Medically fit for general anaesthetic

Exclusion Criteria

- First line chemotherapy other than FOLFIRINOX
- Concomitant malignancy (except skin and prostate cancers)
- Metastatic/Borderline resectable disease
- Untreated gastric outlet/biliary obstruction
- Co-morbidity precluding general anaesthesia +/- neuromuscular blockade
- Indwelling electrical devices
- CKD≥3
- History of neurological disease
- Abdominal varices
- Active bleeding
- Anticoagulation which cannot be discontinued,
- Coagulopathy (INR>1.5)/**any one** of the following:
    - Hb< 8 g/dL
    - absolute neutrophil count< 1500 cells/mL
    - platelet count< 50,000

For peer review only

Treatment of unresectable Locally Advanced Pancreatic cancer with Percutaneous Irreversible Electroporation (IRE) following initial systemic chemotherapy (LAP-PIE) trial: Study protocol for a feasibility randomised controlled trial.

Protocol

Version II- August 2021

Study Principal Investigator:	Brian R. Davidson, MB ChB, MD, FRCS (Eng.), FRCPS (Glasg), Division of Surgery and Interventional Science, University College London
Sponsors:	Royal Free London NHS Foundation Trust
Protocol contributors:	Zainab Rai, (Division of Surgery and Interventional Science, University College London)<br>Veronica Ranieri, (Division of Surgery and Interventional Science, University College London)<br>Michael Stackpoole, (Liverpool Clinical Trials Unit, Liverpool)<br>Peter Littler, (Interventional Radiology, Freeman Hospital, Newcastle)<br>Dan Palmer, (Liverpool Clinical Trials Unit, Liverpool)<br>Paula Ghaneh, (Liverpool Clinical Trials Unit, Liverpool)<br>Kurinchi Gurusamy, (Division of Surgery and Interventional Science, University College London)<br>Derek Manas, (Newcastle NHS Foundation Trust, Newcastle)<br>Elena Pizzo, (Statistical sciences, University College London)<br>Eftychia E Psarelli, (University of Liverpool)<br>Roopinder Gilmore(Royal Free London NHS Foundation Trust)<br>Praveen Peddu (King's College Hospital NHS Trust),

For peer review only - http://bmjopen.bmj.com/site/about/guidelines.xhtml

David C Bartlett, (University Hospitals Birmingham NHS Foundation Trust Birmingham)
Nico de'Liguori Carino (Manchester Royal Infirmary)
Brian Davidson, (Centre for Surgical Innovation, Organ Regeneration and Transplant University College London)

Trial identifiers: Trial registered with International Standard Randomised Controlled Trial Number at https://www.isrctn.com/ISRCTN14986389
Trial ID: ISRCTN14986389
Date registered: 09/02/2021
PB-PG-1217-20042

For peer review only

For peer review only - http://bmjopen.bmj.com/site/about/guidelines.xhtml

REVISION HISTORY

Version I	Date 29/10/2020	Amendment Text	Description
Version II	Date 14/08/2021		As per Reviewer's comments

For peer review only

For peer review only - http://bmjopen.bmj.com/site/about/guidelines.xhtml

Table of Contents

1. TRIAL SUMMARY ....
2. INTRODUCTION ....
2.1. Background and rationale..
2.2. Objectives...
2.3. Trial design ....
3. METHODS...
3.1. Study setting..
3.2. Eligibility criteria...
3.2.1. Inclusion criteria ....
3.2.2. Exclusion criteria ....
3.3. Interventions ....
3.3.1. Intervention description ....
3.3.2. Modifications..
3.3.3. Adherence ....
3.3.4. Concomitant care ....
3.4. Outcomes ....
3.5. Participant timeline ....
3.6. Sample size ....
3.7. Recruitment ....
3.8. Allocation ....
3.9. Blinding (masking) ....
3.9.1. Blinding mechanism ....
3.9.2. Emergency unblinding...
3.10. Data collection ....
3.10.1. Trial procedures and evaluations ....
3.10.2. Retention ....
3.11. Data management..
3.12. Statistical methods ....
3.12.1. Outcomes ....
3.12.2. Additional analyses ....
3.12.3. Analysis population and missing data ....
3.13. Data monitoring ....
3.13.1. Formal committee...
3.13.2. Interim analysis ....
3.14. Safety/harms ....
3.15. Auditing..
4. ETHICS AND DISSEMINATION...
4.1. Research ethics approval ....
4.2. Protocol amendments...
4.3. Informed consent process ....
4.4. Confidentiality ....
4.5. Declaration of interests...
4.6. Access to data ....
4.7. Dissemination policy..

For peer review only - http://bmjopen.bmj.com/site/about/guidelines.xhtml

4.7.1. Trial results ....
4.7.2. Authorship ....
4.7.3. Reproducible research ....
5. STUDY ADMINISTRATION ....
5.1. Key contacts ....
5.2. Funders ....
5.3. Roles and responsibilities ....
5.3.1. Protocol contributors ....
5.3.2. Sponsor and funder ....
5.3.3. Trial committees ....
6. REFERENCES ....
7. APPENDICES ....
7.1. Informed consent materials ....

For peer review only

For peer review only - http://bmjopen.bmj.com/site/about/guidelines.xhtml

1. TRIAL SUMMARY

World Health Organization Registration Data Set

Title	Treatment of unresectable Locally Advanced Pancreatic cancer with Percutaneous Irreversible Electroporation (IRE) following initial systemic chemotherapy (LAP-PIE) trial: Study protocol for a feasibility randomised controlled trial.
Primary registry and trial identifying number	Trial registered with International Standard Randomised Controlled Trial Number at https://www.isrctn.com/ISRCTN14986389<br>Trial ID: ISRCTN14986389<br>Date registered: 09/02/2021
Secondary identifying numbers	PB-PG-1217-20042
Sources of monetary or material support	Sources and types of financial, material, and other support<br>**Funding body:**<br>National Institute for Health Research (NIHR), Research for Patient Benefit (RfPB) programme<br>Central Commissioning Facility<br>Grange House<br>15 Church Street<br>Twickenham<br>TW1 3NL<br>**Training support:**<br>Angiodynamics. Manufacturer of CE marked device that is in use in clinics already. Angiodynamics will provide training and support as normal during this study and supply of the needle probes used through the duration.<br>**Other Support.**<br>Operational Director Liverpool Clinical Trials Centre Block C, Waterhouse Building1-3 Brownlow StreetLiverpoolL69 3GLTel: 0151 794 8897Fax: 0151 794 8931LAP-PIE@liverpool.ac.uk
Primary sponsor	Royal Free London NHS Foundation Trust
Secondary sponsors (if any)	Not applicable
Central contact	Operational Director<br>Liverpool Clinical Trials Centre Block C, Waterhouse Building 1-3 Brownlow Street Liverpool L69 3GL<br>0151 794 8897

For peer review only - http://bmjopen.bmj.com/site/about/guidelines.xhtml

	LAP-PIE@liverpool.ac.uk
Study officials/Investigators	Brian R. Davidson, MB ChB, MD, FRCS (Eng), FRCPS (Glasg)<br>Division of Surgery and Interventional Science, University College London<br>Royal Free Hospital, 9th Floor (East), 2QG, 10 Pond St, London NW3 2PS<br>020 7794 0500, Ext.33603<br>b.davidson@ucl.ac.uk<br>Study Principal Investigator
Brief title	Treatment of unresectable Locally Advanced Pancreatic cancer with Percutaneous Irreversible Electroporation (IRE) following initial systemic chemotherapy: a feasibility randomised controlled trial
Acronym	LAP-PIE
Countries of recruitment	United Kingdom
Condition(s) or focus of study	Locally advanced pancreatic cancer, Irreversible electroporation, Feasibility trial
Interventions	
IRE (interventional)	Intervention type: Device<br>Intervention name: Irreversible Electroporation<br>Other names: IRE<br>Intervention description: The length of the electrical pulse and amperage will be decided by the treating interventional radiologist and will be between 70-100 microseconds with an amperage of between 20-40 AMPs. The treating interventional radiologist will have relevant expertise in IRE procedures with a minimum of 5 IRE procedures on liver or pancreas performed previously.
Standard of Care (control)	Intervention type: Drug<br>Intervention name: 5-FluoroUracil, Leucovorin, Irinotecan and Oxaliplatin<br>Other names: FOLFIRINOX<br>Intervention description: Patients randomised to receive further chemotherapy will commence treatment within 6 weeks after sMDT or 4 weeks after randomisation, whichever is sooner. The second line chemotherapy

For peer review only - http://bmjopen.bmj.com/site/about/guidelines.xhtml

	regime will be selected at the discretion of the treating oncologist and will adhere to NICE guidelines and local protocols.
Key eligibility criteria	Age eligibility: 18 years or older<br>Sex eligibility: Both<br>Accepts healthy volunteers: No<br><br>Inclusion criteria:<br>1. Tissue confirmation<br>2. Successful completion of FOLFIRINOX<br>3. Cancer deemed inoperable at sMDT review,<br>4. WHO Performance status 0 /1<br>5. Maximum cancer diameter of 3.5 cm at time of IRE treatment<br><br>Exclusion criteria:<br>1. First line chemotherapy other than FOLFIRINOX<br>2. Concomitant malignancy (except skin and prostate cancers)<br>3. Metastatic/Borderline resectable disease<br>4. Untreated gastric outlet/biliary obstruction<br>5. Co-morbidity precluding general anaesthesia +/- neuromuscular blockade<br>6. Indwelling electrical devices<br>7. Chronic Kidney Disease > 3<br>8. History of neurological disease<br>9. Abdominal varices<br>10. Active bleeding<br>11. Anticoagulation which cannot be discontinued<br>12. Coagulopathy
Study design	Study type: Interventional trial<br>Allocation: Randomized<br>Intervention model: Parallel group<br>Primary purpose: Other<br>Phase: Feasibility
Masking	None
Date of enrolment	
Target sample size	50
Recruitment status	Active, not recruiting

For peer review only
For peer review only - http://bmjopen.bmj.com/site/about/guidelines.xhtml

Primary outcomes	To ascertain the feasibility of identifying and recruiting LAPC patients at the selected sites to the LAP-PIE trial. The primary outcome consists of recruitment rate, number of screening failures, number of patients completing study pathway as per protocol and trial withdrawal rate. Timeframe: 36 months
Secondary outcomes	To assess the practicality and technical success of pancreas IRE treatment. Completion rates of IRE procedures, safety and complications of IRE treatment, technical success of intervention, acceptability of pancreas IRE therapy to patients and their clinicians, rate of withdrawal from the study and outcome measures including overall survival (measured from diagnosis and randomisation), progression free survival (measured as a binary outcome) and quality of life will be evaluated. Timeframe: 36 months

For peer review only

For peer review only - http://bmjopen.bmj.com/site/about/guidelines.xhtml

2. INTRODUCTION

2.1. Background and rationale

Surgery (pancreatic resection) is the only treatment with the potential to cure pancreatic cancer. However only 10 to 20% of people are eligible for surgery. One of the major reasons is that the cancer has spread into the surrounding structures, often the superior mesenteric vein and/or artery. This circumstance affecting about 30% of all patients is termed locally advanced pancreatic cancer (LAPC). Currently, the recommended treatment for LAPC is chemotherapy. Those with a response to chemotherapy would again be considered for resection. Overall 65% of patients post chemotherapy remain with localised but unresectable disease (UK SCALOP trial induction protocol data).

These individuals will be invited to participate in this study.

Irreversible electroporation (IRE) is a new method of treating localised cancers which are not suitable for surgery. The IRE treatment destroys cancer cells using electricity. Under general anaesthetic the IRE electrodes are inserted into the borders of the pancreas cancer prior to an electrical charge being passed through the cancer. Early studies of IRE in pancreas cancer show that IRE treatment may increase how long people with LAPC live but these studies were not controlled or randomised. The improved outcome could therefore be related to patient selection. In addition, major complications may result from this treatment (eg acute pancreas inflammation, acute pancreatitis), particularly when the treatment is carried out at open surgery rather than percutaneously.
There is therefore uncertainty on the clinical application of pancreas IRE therapy.

2.2. Objectives

Primary Objective:
To ascertain the feasibility of performing a Randomised Controlled Study on Irreversible Electroporation following initial systemic chemotherapy for Locally Advanced Pancreatic Cancer.

Secondary Objectives:
To Assess:
1. Recruitment to a trial of this nature
2. The practicality and technical success of pancreas IRE treatment
3. The acceptability of pancreas IRE therapy to patients and their clinicians
4. Rate of withdrawal from the study
5. Safety of the IRE treatment
6. Relative importance of different outcome measures (Survival and Quality of Life)

2.3. Trial design

For peer review only - http://bmjopen.bmj.com/site/about/guidelines.xhtml

Trial Design
The LAP-PIE trial is a UK multi-centre, feasibility study that will recruit 50 patients with LAPC in whom the disease has remained localised and unresectable following FOLFIRINOX chemotherapy. All participants will be offered further chemotherapy according to standard centre protocols and half will also be offered IRE. It is an open-label study.

3. METHODS

3.1. Study setting

We are proposing to run a small-scale study (a "feasibility study") that will recruit 50 people with LAPC whose disease has remained localised to the pancreas but is unresectable following initial chemotherapy. All will be offered further chemotherapy according to standard centre protocols and half will also be offered IRE. We will assess whether patients are willing to participate in this study and complete the designated treatment. We will assess the completeness of pancreas IRE therapy (technical success) and the complications of the procedure. We will follow the study participants for 12 months assessing their quality of life and cancer response from imaging.

Nested within the feasibility trial there are qualitative and translational components. The qualitative study will assess patients' and clinicians' perspectives surrounding involvement and participation in a trial of this nature. The translational component aims to determine whether there is a biological significance between post-IRE imaging and peripheral immune response and treatment efficacy.

Each centre will complete a feasibility questionnaire to ensure that the centre has the appropriate facilities, qualified personnel and capacity to set up and run the trial in accordance with the current approved protocol and GCP standards.

A list of study sites can be obtained from contacting the central enquiry for the LAP-PIE trial at LAP-PIE@liverpool.ac.uk

3.2. Eligibility criteria

3.2.1. Inclusion criteria

1. Tissue confirmation
   Histopathological confirmation of pancreatic adenocarcinoma
2. Successful completion of FOLFIRINOX
3. Cancer deemed inoperable at sMDT review,
   sMDT (specialist multi-disciplinary team)
4. WHO Performance status 0 /1
   World Health Organisation (WHO)

For peer review only - http://bmjopen.bmj.com/site/about/guidelines.xhtml

5. Maximum cancer diameter of 3.5 cm at time of IRE treatment
   Retrospective and prospective studies suggest minimal clinical benefit of treating tumours > 3.5cm

3.2.2. Exclusion criteria

1. • First line chemotherapy other than FOLFIRINOX
2. • Concomitant malignancy (except skin and prostate cancers)
3. • Metastatic/Borderline resectable disease
4. • Untreated gastric outlet/biliary obstruction
5. • Co-morbidity precluding general anaesthesia +/- neuromuscular blockade
6. Indwelling electrical devices
7. Chronic Kidney Disease > 3
8. History of neurological disease
9. Abdominal varices
10. Active bleeding
11. Anticoagulation which cannot be discontinued
12. Coagulopathy
    (INR>1.5)/any one of the following, Hb< 8 g/dL, absolute neutrophil count< 1500 cells/mL, platelet count< 50,000.

3.3. Interventions

3.3.1. Intervention description

Intervention

Irreversible electroporation (IRE)

This new form of cancer treatment involves application of electric pulses to increase the permeability of cancer cell membranes. IRE induces cancer cell apoptosis which peaks at about 24 hours after IRE is performed. IRE has been used for ablation of several solid cancers including liver, pancreatic, and prostate cancers on an experimental basis. In pancreatic cancer, IRE has been used in patients for ablation of locally advanced pancreatic cancer either by open surgery, laparoscopically or percutaneously under CT (computed tomography) guidance or ultrasound guidance. In this study all IRE will be delivered percutaneously with CT guidance. The radiologist delivering IRE treatment in this study will be required to have performed a minimum of 5 previous liver or pancreas IRE ablations prior to delivering treatment to patients enrolled in the trial. Post-IRE treatment, patient will be admitted for a minimum 24 hours for pain relief and monitoring and management of immediate procedure-related complications.

Controls (Second line systemic chemotherapy)

Patients randomised to receive further chemotherapy will commence treatment within 6 weeks after sMDT or 4 weeks after randomisation, whichever is sooner. The second line chemotherapy regime will be selected at the discretion of the treating oncologist and will adhere to NICE guidelines and local protocols.

For peer review only - http://bmjopen.bmj.com/site/about/guidelines.xhtml

3.3.2. Modifications

Premature Discontinuation of treatment

Participants may discontinue IRE or standard of care chemotherapy for reasons including, but not limited to:

- Participant-led i.e. request by the participant / person with parental responsibility / legal representative / consultee
- Unacceptable toxicity/side effects
- Intercurrent illness preventing further treatment.
- Pregnancy
- Death
- Clinician-led:
  - Any change in the participant's condition that justifies the discontinuation of treatment in the clinician's opinion.
  - Reasons of non-adherence or non-compliance with treatment or other trial procedures
  - Participant meets an exclusion criterion (either newly developed or not previously recognised)

3.3.3. Adherence

All sites will have a nominated clinical lead in each respective speciality (Surgery, Interventional Radiology and Oncology) who will be responsible for adhering to intervention protocol. They will be invited to attend Investigator meetings and be kept abreast of any changes to the protocol/progress of the trial.

Both the interventional arm (IRE) and the Standard of Care (Chemotherapy) requires patients to attend hospital and therefore no specific adherence strategies have been developed. Participants will be encouraged to attend these appointments.

3.3.4. Concomitant care

Concomitant Medications/Treatments

Concomitant treatment for conditions other than Locally Advanced Pancreatic Cancer may be continued/started throughout the trial in their standard dose as long as it does not affect inclusion or exclusion criteria. Use of concomitant treatment must be recorded in the patient medical record and study CRFs: the name of the drug, dose, start/stop dates and the indication for it are necessary.

Use of any other non-marketed or investigational products during the trial are NOT permitted.

Supportive medications such as analgesics, anti-emetics etc. should be given per local practice. Analgesic use prior to commencing second line therapy should be documented in the Case Report Form (CRF) with drugs, dose and frequency of use.

For peer review only - http://bmjopen.bmj.com/site/about/guidelines.xhtml

Medications Permitted

All medication necessary for the wellbeing of the patient, which is not expected to interfere with the evaluation of the IRE therapy and aftercare may be administered. The local investigator may contact the study team for guidance from the Chief Investigator or delegated medical expert. Particular attention should be given to treatment related to the side effects and risks of the IRE therapy.

IRE will be carried out under general anaesthetic and neuromuscular relaxation. Broad spectrum antibiotics will be administered pre and for 2 doses post procedure.

Medications Not Permitted/ Precautions Required

There are no medications which would contra-indicate pancreas IRE therapy.
As with all interventions patients with abnormal blood clotting or taking anticoagulant medication should be considered with caution and withdrawal of anticoagulant medication or alternative short-lived anticoagulant administered until the patient recovers from the procedure at which time routine medication will be re-commenced.

Data on Concomitant Medication

The systemic chemotherapy (drug/dose/duration) will be recorded for both treatment groups over the 12 month follow up period. Concomitant medication will be collected at baseline and updated through the study duration. Each visit CRF will prompt the local research team to complete the concomitant medication form for any new drugs and to ensure the start and stop date of previous medication is stated.

3.4. Outcomes

Outcomes

The primary objective of the LAP-PIE trial is to ascertain feasibility of such a trial concept. In particular, the ability to identify and recruit LAPC patients at the selected sites to the LAP-PIE trial will be evaluated. The primary outcome consists of recruitment rate, number of screening failures, number of patients completing study pathway as per protocol and trial withdrawal rate.

The secondary objectives of the LAP-PIE trial aim to assess the practicality and technical success of pancreas IRE treatment. Completion rates of IRE procedures, safety and complications of IRE treatment, technical success of intervention, acceptability of pancreas IRE therapy to patients and their clinicians, rate of withdrawal from the study and outcome measures including overall survival (measured from diagnosis and randomisation), progression free survival (measured as a binary outcome) and quality of life will be evaluated.

3.5. Participant timeline

For peer review only - http://bmjopen.bmj.com/site/about/guidelines.xhtml

Schedule of enrolment, interventions, and assessments of the LAP-PIE trial.

	**Study Period**									
	**Enrolment**	**Allocation**	**Post-Allocation**							**Close-out**
**TIMEPOINT**	***-t₁***	**0**	***Pre-IRE***	***6 hours post -IRE***	***6 days post -IRE***	***3 months***	***6 months***	***9 months***	***12 months***	
**ENROLMENT:**										
**Eligibility screen**	X									
**PIS given**	X									
Systemic treatment with FOLFIRINOX	X									
sMDT	X									
Informed consent	X									
**Allocation**		X								
**INTERVENTIONS:**										
IRE (intervention)			◆———	———	———	———	———	———	———◆	
Systemic chemotherapy (SoC)						◆———	———	———	———◆	
**ASSESSMENTS:**										
CT CAP	X					X	X	X	X	
Blood tests	X		X	X	X	X	X	X	X	X
HRQoL						X	X	X	X	X
Complications						X	X	X	X	X

PIS; Patient Information Sheet, FOLFIRINOX; 5-FluoroUracil, Leucovorin, Irinotecan and Oxaliplatin, sMDT; specialist Multi-Disciplinary Team, HRQoL; Health Related Quality of Life

For peer review only

For peer review only - http://bmjopen.bmj.com/site/about/guidelines.xhtml

3.6. Sample size

A convenient sample size of 50 patients, randomised equally to the two groups, has been chosen for this feasibility study. Formal sample size calculations are not appropriate for a feasibility study. However, this size will allow us to assess whether the consent rate of 20% has been achieved with a 95% confidence interval of +/- 11%. This sample size will also allow us to assess whether the technical success of IRE is 50% with a 95% confidence interval of +/- 14%, and loss to follow-up is 10% with a 95% confidence interval of +/-8%. In addition, sample sizes between 24 and 50 have been recommended to estimate the standard deviation required for a sample size calculation to allow us to design a large randomised controlled trial aimed at evaluating the cost effectiveness of IRE following chemotherapy for locally advanced pancreatic cancer (24, 25).

The recruitment target is 50 patients in 15 months from 7 UK centres, assuming a recruitment rate of 0.8 patients per site per month

3.7. Recruitment

This is a feasibility trial and one key aspect of feasibility evaluation is the ability to recruit patients with unresectable locally advanced pancreatic cancer into a trial of this nature. We will explore the reasons for participation and non-participation of patients and patients' and clinicians' acceptability of the trial to assist in optimisation of recruitment strategies employed for the subsequent trial. Non-participation can be related to how the clinical trial is presented to the patient, and how the patient assimilates this information. It is therefore important to understand how patients perceive information about potential participation and their experiences of receiving information on the trial.

3.8. Allocation

Allocation Sequence Generation

Participants will be randomised on a 1:1 basis. Randomisation lists shall be produced by the trial statistician using randomly permuted blocks Lists created using Stata (Version 14 or higher) using the add-on package 'ralloc'

Patient allocations will be irrevocably generated upon completion of the web-based randomisation form by a delegated member of the trial research team. Allocation concealment will be ensured as the service will not release the randomisation code until the patient has been recruited into the trial; this takes place after all baseline measurements have been completed.

For peer review only - http://bmjopen.bmj.com/site/about/guidelines.xhtml

3.9. Blinding (masking)

3.9.1. Blinding mechanism

The intervention is a procedure requiring expertise and general anaesthesia therefore it is not possible or appropriate to design this study as a blinded trial

3.9.2. Emergency unblinding

Not a blinded study therefore not applicable

3.10. Data collection

3.10.1. Trial procedures and evaluations

Data Collection Methods

Each participating site will maintain appropriate medical and research records for this trial, in compliance with ICH E6 GCP, Section 4.9 and regulatory and institutional requirements for the protection of confidentiality of subjects.

Data that is to be recorded directly on the electronic Clinical Record Forms (eCRF) or workbooks (i.e. no prior electronic or written record of the data), is to be considered to be source data e.g. questionnaires

Date(s) of informed consent processes (including date of provision of patient information, randomisation number and the fact that the patient is participating in a clinical trial (including possible treatment arms) will be added to the patient's medical record chronologically.

Data will be entered into MACRO and PHAROS by members of the research team at site. Training will be provided prior to any data entry. The eCRF workbooks that replicate each MACRO and PHAROS form must be retained at site for the study.

Questionnaires are a source document and sites will retain a copy at site in the Investigator Site File (ISF) (part of the Trial Master File).

Quality Assurance and Control

Quality assurance will be maintained to the required level via electronic database validations, internal LCTC quality processes and procedures, training of those involved in trial procedures and regular monitoring and audit.

The TMG will be involved in monitoring screening, randomisation and consent rates, as well as compliance with processes detailed in the protocol. Any activity that is determined to be a breach of the study protocol will be recorded on a protocol deviation form and reviewed by the study CI to confirm severity, the impact on the study and whether any corrective and preventative action is required.

The LAP-PIE study will have a designated data management and query resolution plan in place and specific instructions on how to complete the eCRF forms and workbooks. A

For peer review only - http://bmjopen.bmj.com/site/about/guidelines.xhtml

For peer review only

member of the study team will periodically review the study for missing or erroneous/anomalous data based on validations built into the database.

Study Instruments

The EQ5D-5L health questionnaire will be used at Baseline and each follow up visit in order to assess quality of life for all patients on the study. The EQ5D-5L is descriptive system for health-related quality of life states in adults, consisting of five dimensions (Mobility, Self-care, Usual activities, Pain & discomfort, Anxiety & depression), each of which has five severity levels that are described by statements appropriate to that dimension. It has been used to assess quality of life in a number of conditions (ref) and is considered a reliable and valid health related questionnaire (ref).

Data collection forms can be requested by contacting the central LAP-PIE trial team on LAP-PIE@liverpool.ac.uk

3.10.2. Retention

Plans to Promote Participant Retention and Complete Follow-up.

Specific plans to promote participant retention and trial completion are not appropriate for a feasibility trial because part of the primary outcome of feasibility is understanding the reasons for discontinuation. This information will be used to plan a larger randomised control trial where participant retention methods will be developed based on information from this feasibility trial.

Outcome Data to be collected for participants who discontinue/deviate from interventions protocols

Discontinuation from study intervention does not mean discontinuation of the study altogether, and the remaining study procedures, follow up assessment / visits and data collection should be completed as indicated in the protocol (unless consent is specifically withdrawn). Patients finishing the study should have an end of Study eCRF completed, (if due to patient death, there is a notification of death eCRF), detailing the reason for not continuing.

Participant Withdrawal from Follow Up visits

Patients are free to withdraw from follow up at any time without providing a reason, though a reason should be recorded if one is given. Those who wish to withdraw from further follow-up will have the data collected up to the point of that withdrawal included in the analyses. The participant will not contribute further data to the study and the CTU should be informed. The End of Study eCRF should be completed to notify the CTU of this withdrawal.

If patients express a wish to withdraw from follow up, the research team at site should ascertain if this is for all elements of trial follow-up, or if for example data from routine

For peer review only - http://bmjopen.bmj.com/site/about/guidelines.xhtml

assessments can still be collected for the trial. In the case of ongoing adverse events, participants should be given appropriate care under medical supervision until the symptoms of any adverse event resolve or the patient's condition becomes stable. Any SAEs will be notifiable to the CTU via processes detailed in Section 11 even if a participant has withdrawn from follow up.

3.11. Data management

Confidentiality

Patients will consent for the use of their data, including identifiable data such as NHS number and Date of Birth before taking part in the study. Though the consent form will have their name and NHS number, this data will not be used on any eCRF or Workbook. Patients will instead be referred to using their screening number, and then randomisation number once allocated to one of the study arms.

Consent forms with patient identifiable information will be kept within the patient file at the local research site and a copy at the LCTC to evidence that the patient consented to study. At both locations the consent forms will be stored within a locked cabinet/drawer within a secure office and department.

The LCTC as part of The University of Liverpool will preserve the confidentiality of participants taking part in the study. The University of Liverpool is registered as a Data Controller with the Information Commissioners Office.

Breaches of data protection principles or regulations identified by LCTC will be notified promptly to the trial Sponsor and The University of Liverpool's Data Protection Officer and appropriate processes followed

Quality Assurance and Control

Quality assurance will be maintained to the required level via electronic database validations, internal LCTC quality processes and procedures, training of those involved in trial procedures and regular monitoring and audit.

The TMG will be involved in monitoring screening, randomisation and consent rates, as well as compliance with processes detailed in the protocol. Any activity that is determined to be a breach of the study protocol will be recorded on a protocol deviation form and reviewed by the study CI to confirm severity, the impact on the study and whether any corrective and preventative action is required.

The LAP-PIE study will have a designated data management and query resolution plan in place and specific instructions on how to complete the eCRF forms and workbooks. A member of the study team will periodically review the study for missing or erroneous/anomalous data based on validations built into the database.

For peer review only - http://bmjopen.bmj.com/site/about/guidelines.xhtml

The frequency of the LAP-PIE Trial Management Group meeting will be outlined in the monitoring plan, along with what data in the Central Monitoring Report will be reviewed

3.12. Statistical methods

3.12.1. Outcomes

Primary Outcome

1. Recruitment rate will be measured by:

(I) the total number of patient participants randomised per month and

(ii) ratio of successful recruitment to eligible patients approached.

1. Number of screening failures.
2. Number of patients completing study pathway as per protocol will be measures as the number of randomised patients completing the full study pathway with no major protocol deviations.
3. Trial withdrawal rate will be measured using the total number of patients randomised in the study. In addition, withdrawal rates will be presented by trial withdrawal reasons and withdrawal stage for both arms.

Secondary Outcomes

Adherence to protocol

The number of minor and major protocol deviations will be collected on a patient participant and site level.

Mortality rate

Overall survival will be recorded for all patients.

1. Survival is defined as the number of days between date of randomisation and date of death due to any cause or the date of the last follow-up if the patient is still alive at the time of final trial follow up at 12 months post randomisation.
2. Survival is defined as the number of days between date of diagnosis and date of death due to any cause or the date of the last follow-up if the patient is still alive at the time of final trial follow up at 12 months post randomisation.

An overall survival rate will be calculated as also a 9-month using both definitions.

Technical success

Proportion of patients with a successful percutaneous IRE (complete local therapy) as defined in section 10.8 (Assessing Efficacy)

Surgical rate.

It is possible that patients will be considered for surgery having completed second line therapy (IRE and chemo or chemo alone). If so, the following will be recorded:

1. Number of patients who become eligible for surgery over the total number of patients recruited.
2. Number of patients proceeding to surgery over the total number of patients who become eligible for surgery.

For peer review only - http://bmjopen.bmj.com/site/about/guidelines.xhtml

Resection rate (R0/R1)
The ratio will be calculated over numbers undergoing tumour resection in each arm of the trial. Pathology of the specimens will determine the overall R0/R1 resection rate. The R0 resection margin will be recorded according to the Royal College of Pathologists report on Standards and datasets for reporting cancers.

Local or systemic disease progression on follow up
The proportion of patients with local or systemic disease progression as defined by RECIST criteria in each arm of the trial will be calculated at 3,6,9 and 12 months post.

Chemotherapy-related toxicities.
Formal toxicity assessments will be performed and graded according to section 12. The proportion of patients experiencing grade 3/4 toxicity will be calculated.

IRE Procedure related complications
Complications related to IRE therapy will be recorded and the severity grading will be performed according to Clavien-Dindo classification. The proportion of patients experiencing grade III and above toxicity will be calculated.

Quality of life
Quality of life will be assessed with the EQ-5D-5L. The questionnaires will be completed as per the Schedule of Trial Procedures in Section 3 by all patients. The data will be scored according to the EQ-5D-5L scoring manual.

3.12.2. Additional analyses
Not applicable

3.12.3. Analysis population and missing data
Not applicable for a feasibility trial

3.13. Data monitoring
3.13.1. Formal committee
The Data Monitoring Committee (DMC) will consist of an independent chairperson, plus 2 independent members; who are experts in the field of Pancreatic Cancer, and an independent biostatistician.
The DMC will be responsible for reviewing and assessing recruitment, interim monitoring of safety and effectiveness, trial conduct and external data. The DMC will first convene prior to start of recruitment and will then define frequency of subsequent meetings (at least annually).

Further details of its charter can be requested from central enquires on LAP-PIE@liverpool.ac.uk

3.13.2. Interim analysis

For peer review only - http://bmjopen.bmj.com/site/about/guidelines.xhtml

No formal interim analyses will be performed during the course of the feasibility study. The Data and Monitoring Committee (DMC) will assess the actual recruitment rates and make decisions on whether to stop the trial in light of the analyses presented. These analyses will be performed at the LCTC. A decision to discontinue recruitment, in all patients or in selected subgroups will be made only if the result is likely to convince a broad range of clinicians including participants in the trial and the general clinical community. The DMC will make formal recommendations to the Trial Steering Committee (TSC) as to the continuation of the trial.

3.14. Safety/harms

Assessment of Complications

Adverse Events (AE) for the Standard of Care Chemotherapy will be defined using the Common Terminology Criteria for Adverse Events (CTCAE) Version 4.0 (ref). AEs for IRE will be defined using the Clavien Dindo classification of surgical complications (ref). Complications greater than grade 3 on the CTCAE scale and greater than grade III on the Clavien Dindo scale will be reported.

A Serious Adverse Event (SAE) for patients randomised to receive Standard of Care Chemotherapy only, will be compared against the reference safety information (RSI) within the current summary of product characteristics (SmPC) for that drug. The RSI will be taken from the latest SmPC available for the drug on https://www.medicines.org.uk/emc. The RSI for IRE will be from the Nanoknife system users' manual.

All safety events which are recorded for the study will be reported following the procedures detailed below. The occurrence of a safety event may come to the attention of research staff during routine study visits, from the participant's notes, directly from the participant or by other means. Note that reporting procedures vary dependent on the nature of the incident (i.e. "serious" events are to be reported to LCTC in an expedited manner). Any questions concerning adverse event reporting should be directed to the LCTC in the first instance. A flowchart is provided in the additional documents to aid in determining reporting procedures for different types of adverse events.

Reporting Safety Events to the LCTC

SAE reporting will be done from the date of randomisation until the end of study period at 12 months following randomisation

All AEs, whether serious or non-serious, that occur during this timeframe will be recorded via the LCTC's pharmacovigilance database: https://lctu.org.uk/PV/login.aspx

Sites will use the LCTC pharmacovigilance database to report adverse, instruction on how to enter AEs on the pharmacovigilance database will be provided to research staff at site initiation as a work instruction. Paper CRFs will be available for download from the LCTC PORTAL for use only in case the pharmacovigilance database is not working.

For peer review only - http://bmjopen.bmj.com/site/about/guidelines.xhtml

These can be sent in to the LCTC via Fax for upload by the LAP-PIE Study Coordinator or Data Manager.

Follow-up After Adverse Events
All reportable adverse events will be followed until satisfactory resolution or until the investigator responsible for the care of the participant deems the event to be chronic or the patient to be stable.

When reporting "serious" safety events the investigator responsible for the care of the participant will apply the following criteria to provide information relating to event outcomes:

- resolved
- resolved with sequelae (specifying with additional narrative)
- not resolved/ongoing
- ongoing at final follow-up
- fatal or unknown.

3.15. Auditing

Quality assurance will be maintained to the required level via electronic database validations, internal LCTC quality processes and procedures, training of those involved in trial procedures and regular monitoring and audit.

The Trial Monitoring Group (TMG) will be involved in monitoring screening, randomisation and consent rates, as well as compliance with processes detailed in the protocol. Any activity that is determined to be a breach of the study protocol will be recorded on a protocol deviation form and reviewed by the study CI to confirm severity, the impact on the study and whether any corrective and preventative action is required.

Independent oversight of the trial will be provided by the members of the DMC.

The LAP-PIE study will have a designated data management and query resolution plan in place and specific instructions on how to complete the eCRF forms and workbooks. A member of the study team will periodically review the study for missing or erroneous/anomalous data based on validations built into the database.
The frequency of the LAP-PIE TMG meeting will be outlined in the monitoring plan, along with what data in the Central Monitoring Report will be reviewed

4. ETHICS AND DISSEMINATION

4.1. Research ethics approval

Statement of Compliance

The trial involves the use of a CE marked medical device which is utilised for the intended purpose therefore this trial is not within the remit of the Medical Devices Regulation.

For peer review only - http://bmjopen.bmj.com/site/about/guidelines.xhtml

The procedures detailed within this protocol are compliant with the Ionising Radiation (Medical Exposure) Regulations, and appropriate review by a Medical Physics Expert and Clinical Radiation Expert has been undertaken.

Ethical Considerations

The LAP-PIE trial will be conducted in accordance with, but not limited to, the Human Rights Act 1998, the GDPR, Freedom of Information Act 2000 subject to the provisions of sections 41 and 43 thereof, the EU Clinical Trials Directive, the Medicines for Human Use (Clinical Trials) Regulations 2004, the Medicines Act 1968, the Human Tissue Act 2004, ICH GCP, the Declaration of Helsinki 1996 and the NHS Research Governance Framework for Health and Social Care, as amended from time to time.

Where patients agree to take part in the study, they will be informed of how data are recorded, collected, stored and processed and may be transferred to other countries, in accordance with The General Data Protection Regulation (GDPR) (EU) 2016/679.

This study may be terminated at the request of the CI, ISDMC, REC if, during the course of the study, concerns about the safety of the study emerge.
The CI will update the ethics committee of any new information related to the intervention when appropriate and this will also be disseminated to the Principal Investigators at each trial centre.

Patients entering the trial who are randomised to have standard of care (SoC) will continue on systemic chemotherapy. As there have been no previous randomised trials of IRE for pancreas cancer, we do not know whether these patients in the SoC arm will have a better or worse survival and quality of life than those receiving IRE.

The trial will abide by the principles of the World Medical Association Declaration of Helsinki and has been designed to be as pragmatic as possible. The protocol has undergone ethical review by an independent Research Ethics Committee and has received a favourable opinion by Brent REC.

Approvals

The trial protocol will receive approval by a National Research Ethics Committee (NREC), but all participating sites must undergo site specific assessment of capacity and capability. A copy of all site approval documents and a copy of the PIS and ICF on local headed paper should be forwarded to LCTC before patients are entered. The LCTC should receive a confirmation of capacity and capability for each new centre via the site's R&D department

4.3. Informed consent process

Written informed consent is required for all patients participating in the LAP-PIE trial. Discussion of objectives, risks and inconveniences of the trial and the conditions under which it is to be conducted are to be provided to patients by staff with appropriate experience. An appropriate Patient Information Sheet (PIS), describing in detail the trial interventions/products, trial procedures and risks will be approved by an independent

For peer review only - http://bmjopen.bmj.com/site/about/guidelines.xhtml

ethical committee (IEC) and the patient will be asked to read and review the document. Upon reviewing the document, the investigator will explain the research study to the patient and answer any questions that may arise. A contact point where further information about the trial may be obtained will be provided.

In obtaining and documenting consent, the research team will comply with applicable regulatory requirements and will adhere to GCP and to the ethical principles that have their origin in the Declaration of Helsinki.

Informed consent will be re-affirmed throughout the trial and all discussions and consent will be documented appropriately. If a potential participant does not want to provide consent, they do not have to give a reason.

Written informed consent will be sought from patients who will be approached by the study team and invited to consider participation. This is a discussion that will take place within the hospital that the patient is attending for their LAPC treatment and assessments.

The PIS includes a detailed explanation of the study and makes clear that the rights and welfare of the participants will be protected; it will be emphasised that consent may be declined or withdrawn at any time in the future without the quality of care being adversely affected. The research team will facilitate verbal discussions about the research and the consent process, as well as providing answers to any questions that arise.

All the points on the main study section of the consent form are mandatory for the patient to be eligible, they may not agree to only a selection of criteria. There are optional statements on the consent form related to the translational sub studies and the qualitative evaluation. Patients who do not wish to participate in the sub studies or qualitative evaluation are still eligible for the LAP-PIE trial.

After verbal and written information has been provided, the individual seeking consent must ensure that the patient has fully understood all the information and will ask if they are happy to consent to participation in the trial. Where this is the case, written informed consent will be obtained by means of a dated patient signature on the consent form. This will be countersigned and dated by the person who obtained informed consent i.e. the PI or other appropriately qualified member of the research team who has been delegated this responsibility.

The person obtaining consent, i.e. the person signing off on the consent form with the patient, must be the Principle Investigator or delegated other, a medical practitioner capable of explaining the study rationale and the treatment options to the patient.

The original signed document will be retained in the trial site's Investigator Site File (ISF) and copies will be made:

- One copy provided to the patient for their information,

For peer review only - http://bmjopen.bmj.com/site/about/guidelines.xhtml

- One copy transferred to the LCTC
- One copy filed in the participant's medical records.

N.B. Details of the consent process (date, persons involved, version and type of information sheet and consent form used) must also be recorded directly into the participant's medical records.

4.4. Confidentiality

Patients will consent for the use of their data, including identifiable data such as NHS number and Date of Birth before taking part in the study. Though the consent form will have their name and NHS number, this data will not be used on any eCRF or Workbook. Patients will instead be referred to using their screening number, and then randomisation number once allocated to one of the study arms.

Consent forms with patient identifiable information will be kept within the patient file at the local research site and a copy at the LCTC to evidence that the patient consented to study. At both locations the consent forms will be stored within a locked cabinet/drawer within a secure office and department.

The LCTC as part of The University of Liverpool will preserve the confidentiality of participants taking part in the study. The University of Liverpool is registered as a Data Controller with the Information Commissioners Office.

Breaches of data protection principles or regulations identified by LCTC will be notified promptly to the trial Sponsor and The University of Liverpool's Data Protection Officer and appropriate processes followed.

Records Retention

The investigator at each investigational site will make arrangements to store the essential trial documents, including the Investigator Trial File, until the LCTC informs the investigator that the documents are no longer to be retained.

The PI at each investigational site will make arrangements to store the essential trial documents (as defined by ICH GCP guidelines) including the Investigator Site File, the applicable participant medical records for the full length of the trial's retention period and will arrange for confidential destruction at the end of this period as instructed by the Sponsor.

The PI is also responsible for archiving all relevant source documents so that the trial data can be compared against source data after completion of the trial (e.g. in case of inspection from authorities). They must ensure the continued storage of the documents, even if they, for example, leave the clinic/practice or retire before the end of required storage period. Delegation of responsibility for this must be documented in writing.

For peer review only - http://bmjopen.bmj.com/site/about/guidelines.xhtml

4.5. Declaration of interests

None declared

4.6. Access to data

The Trial Management Group (TMG) will have access to the final trial dataset. The TMG consists of the Chief Investigator, other lead investigators (clinical and non-clinical) and members of the Liverpool Clinical Trials Centre.
Individual Clinicians must undertake not to submit any part of their individual data for publication without the prior consent of the Trial Management Group.

4.7. Dissemination policy

4.7.1. Trial results

This will be the first controlled trial of pancreas IRE therapy in locally advanced pancreas cancer and will have implications for pancreas cancer patients in the UK and Internationally. The trial protocol will be published at the outset. Data from the trial will be presented at National and International meetings (AUGIS, IHPBA, NCRI, BASO) and the trial will be published in a high impact journal. Further dissemination of trial results will be to charitable organisations and patient groups including dissemination via social media. This dissemination will be facilitated by trial outcome literature reviewed and disseminated by our PPI members. The study is not powered to show effectiveness and it is likely that the data will form the basis of an efficiency and cost effectiveness application for a larger multi centre trial. Experience in optimising recruitment, site set up and developing local radiology expertise will be crucial for the success of the subsequent trial which will be powered from data collected during the feasibility study.

4.7.2. Authorship

All publications will include a list of participants, and if there are named authors, these will include the trial's Chief Investigator(s), Statistician(s) and Trial Manager(s) involved at least. If there are no named authors (i.e. group authorship) then a writing committee will be identified that will include these people, at least.

4.7.3. Reproducible research

On completion of the research, a Final Trial Report will be prepared and submitted to the REC. The results of LAP-PIE will be published regardless of the results.

There are no plans for granting public access, however data sharing will be supported via access through the Trial Management Group (TMG).

5. STUDY ADMINISTRATION

5.1. Key contacts

Study Principal Investigator

Brian R. Davidson, MB ChB, MD, FRCS (Eng), FRCPS (Glasg)

For peer review only - http://bmjopen.bmj.com/site/about/guidelines.xhtml

Division of Surgery and Interventional Science, University College London
Royal Free Hospital, 9th Floor (East), 2QG, 10 Pond St, London NW3 2PS
020 7794 0500, Ext.33603
b.davidson@ucl.ac.uk

Co-Principal Investigator
Zainab L. Rai, MBBS, BSc, MRCS (Eng)
Division of Surgery and Interventional Science, University College London
Royal Free Hospital, 9th Floor (East), 2QG, 10 Pond St, London NW3 2PS
Zainab.rai@nhs.net

Central contact
Operational Director

Liverpool Clinical Trials Centre Block C, Waterhouse Building 1-3 Brownlow Street
Liverpool L69 3GL
0151 794 8897
LAP-PIE@liverpool.ac.uk

Sponsor
Research & Development Office Lower Ground Floor Finance Office (LG/306)
Royal Free London NHS Foundation Trust
Pond Street London
London, London, NW3 2QG
United Kingdom
020 7317 7558
rf.randd@nhs.net

5.2. Funders

For peer review only

For peer review only - http://bmjopen.bmj.com/site/about/guidelines.xhtml

Sources and types of financial, material, and other support
Funding body: National Institute for Health Research (NIHR), Research for Patient Benefit (RfPB) programme
Central Commissioning Facility
Grange House
15 Church Street
Twickenham
TW1 3NL
Training support: Angiodynamics. Manufacturer of CE marked device that is in use in clinics already. Angiodynamics will provide training and support as normal during this study and supply of the needle probes used through the duration.
Other Support. Operational Director
Liverpool Clinical Trials Centre
Block C, Waterhouse Building
1-3 Brownlow Street
Liverpool
L69 3GL
Tel: 0151 794 8897
Fax: 0151 794 8931
LAP-PIE@liverpool.ac.uk

For peer review only

5.3. Roles and responsibilities

5.3.1. Protocol contributors

Zainab L Rai, Veronica Ranieri, Stackpoole M, Littler P, Palmer D, Paula Ghaneh, Gurusamy K, Manas D, Elena Pizzo, Eftychia E Psarelli, Roopinder Gilmore, Praveen Peddu, David C Bartlett, Nico de'Liguori Carino, Davidson B and the UK LAP-PIE study group

Acknowledgement
This study is funded by the UK NIHR RfPB programme (PB-PG-1217-20042). The views expressed are those of the author(s) and not necessarily those of the NHS, the NIHR or the Department of Health.

5.3.2. Sponsor and funder

Study sponsor and funder had no role in the study design, data collection and management, data analysis and interpretation and were not involved in the decision to submit report for publication. They do not have ultimate authority over any of these activities.

5.3.3. Trial committees

Liverpool Clinical Trial Centre (LCTC) at the University of Liverpool in collaboration with the Chief Investigator, will have overall management responsibility and will be responsible for trial management activities including (but not limited to), budget

For peer review only - http://bmjopen.bmj.com/site/about/guidelines.xhtml

administration, Trial Master File management, safety reporting, data management, randomisation, statistical analysis, participating site coordination and IMP management.

Trial Management Group (TMG)

A Trial Management Group (TMG) will be formed comprising the Chief Investigator, other lead investigators (clinical and non-clinical) and members of the LCTC. A representative from the Sponsor will also be part of the meeting, and any reports or minutes generated will be circulated for their review. The TMG are responsible for monitoring all aspects of the progress and conduct of the trial and will be responsible for the day-to-day running and management of the trial. The TMG will meet fortnightly throughout the study unless more frequent meetings are required.

For peer review only

For peer review only - http://bmjopen.bmj.com/site/about/guidelines.xhtml

6. REFERENCES

(1) Ghaneh P, Costello E, Neoptolemos JP. Biology and management of pancreatic cancer. *Postgrad Med J* 2008;84(995):478-97.

(2) Balaban EP, Mangu PB, Khorana AA, et al. Locally Advanced, Unresectable Pancreatic Cancer: American Society of Clinical Oncology Clinical Practice Guideline. *J Clin Oncol.* 2016;34(22):2654-68.

(3) Ghosn M, Ibrahim T, Assi T, et al. Dilemma of first line regimens in metastatic pancreatic adenocarcinoma. *World J Gastroenterol* 2016;22(46):10124-30.

(4) Rahib L, Fleshman JM, Matrisian LM, et al. Evaluation of Pancreatic Cancer Clinical Trials and Benchmarks for Clinically Meaningful Future Trials: A Systematic Review. *JAMA Oncol* 2016;2(9):1209-16.

(5) Wang C, Liu X, Wang X, et al. Effects of chemoradiotherapy and chemotherapy on survival of patients with locally advanced pancreatic cancer: A meta-analysis of randomized controlled trials. *Medicine (Baltimore)* 2018;97(36):

(6) Excellence NIfHaC. Overview | Guidance | NICE: NICE; [Available from: Pancreatic cancer in adults: diagnosis and management |. 2020. [Cited: 10/2020]. Available from: https://www.nice.org.uk/guidance/ng85..

(7) V P, P F, P R, et al. Combination of Irinotecan (CPT11) and 5-fluorouracil With an Analysis of Cellular Determinants of Drug Activity. *Biochemical pharmacology.* 1998;56(10):

(8) M D, E M, M O-K, et al. Randomized Phase II Study Evaluating Oxaliplatin Alone, Oxaliplatin Combined with Infusional 5-FU, and Infusional 5-FU Alone in Advanced Pancreatic Carcinoma Patients. *Annals of oncology: official journal of the European Society for Medical Oncology.* 2004;15(3):

(9) Suker M, Beumer BR, Sadot E, et al. A patient-level meta-analysis of FOLFIRINOX for locally advanced pancreatic cancer. *Lancet Oncol.* 2016;17(6):801-10.

(10) Mansson C, Brahmstaedt R, Nygren P, et al. Percutaneous Irreversible Electroporation as First-line Treatment of Locally Advanced Pancreatic Cancer. *Anticancer Res* 2019;39(5):2509-12.

(11) Belfiore MP, Ronza FM, Romano F, et al. Percutaneous CT-guided irreversible electroporation followed by chemotherapy as a novel neoadjuvant protocol in locally advanced pancreatic cancer: Our preliminary experience. *Int J Surg* 2015;21(Suppl 1):34-9.

(12) Lafranceschina S, Brunetti O, Delvecchio A, et al. Systematic Review of Irreversible Electroporation Role in Management of Locally Advanced Pancreatic Cancer. *Cancers (Basel)* 2019;11(11):7181.

(13) Hajatdoost L, Sedaghat K, Walker EJ, et al. Chemotherapy in Pancreatic Cancer: A Systematic Review. *Medicina (Kaunas)* 2018;54(3):48.

(14) MP C, KJ C, EK F, et al. Pre-treatment Assessment of Resectable and Borderline Resectable Pancreatic Cancer: Expert Consensus Statement. *Annals of surgical oncology* 2009;16(7):

(15) Network NCC. Pancreatic Adenocarcinoma, Version 2.2017, NCCN Clinical Practice Guidelines in Oncology - NCCN-2017-pancreatic-cancer.pdf: NCCN; 2017 [Available from: http://clubedopancreas.pt/wp-content/uploads/2018/06/NCCN-2017-pancreatic-cancer.pdf.

For peer review only - http://bmjopen.bmj.com/site/about/guidelines.xhtml

(16) Excellence NIfHaC. Recommendations. Pancreatic cancer in adults: diagnosis and management | Guidance | NICE: NICE. 2020. [Cited: 10/2020]. Available from: https://www.nice.org.uk/guidance/ng85/chapter/Recommendations#managing-resectable-and-borderline-resectable-pancreatic-cancer..
(17) Wills-Percy. St Luke's Cancer Alliance: Cancer treatment 2018. 2018. [Cited: 10/2020]. Available from: http://stlukescanceralliance.co.uk/wp-content/uploads/2018/08/Gem-Cap-V3-8.18.pdf.
(18) Hurt CN, Falk S, Crosby T, et al. Long-term results and recurrence patterns from SCALOP: a phase II randomised trial of gemcitabine- or capecitabine-based chemoradiation for locally advanced pancreatic cancer. *Br J Cancer* 2017;116(10):1264-70.
(19) He C, Wang J, Sun S, et al. Irreversible electroporation versus radiotherapy after induction chemotherapy on survival in patients with locally advanced pancreatic cancer: a propensity score analysis. *BMC Cancer.* 2019;19(394):
(20) Narayanan G, Hosein PJ, Beulaygue IC, et al. Percutaneous Image-Guided Irreversible Electroporation for the Treatment of Unresectable, Locally Advanced Pancreatic Adenocarcinoma. *J Vasc Interv Radiol.* 2017;28(3):342-8.
(21) Kluger MD, Epelboym I, Schrope BA, et al. Single-Institution Experience with Irreversible Electroporation for T4 Pancreatic Cancer: First 50 Patients. *Ann Surg Oncol.* 2016;23(5):1736-43.
(22) Liu S, Qin Z, Xu J, et al. Irreversible electroporation combined with chemotherapy for unresectable pancreatic carcinoma: a prospective cohort study. *Onco Targets Ther* 2019;12(5):1341-50.
(23) W F, JW R, RCG M. Quality of Life Assessment for Patients Undergoing Irreversible Electroporation (IRE) for Treatment of Locally Advanced Pancreatic Cancer (LAPC). *American journal of surgery.* 2019;218(3):
(24) Julious SA.. Sample size of 12 per group rule of thumb for a pilot study.. *Pharmaceutical Statistics* 2005;4(4):287-91.
(25) Sim J, Lewis M.. The size of a pilot study for a clinical trial should be calculated in relation to considerations of precision and efficiency.. *J Clin Epidemiology.* 2012;65(3):301-8.

For peer review only

For peer review only - http://bmjopen.bmj.com/site/about/guidelines.xhtml

7. APPENDICES

7.1. Informed consent materials

**PATIENT CONSENT FORM (please read carefully)**
**LAP-PIE:** Treatment of unresectable **L**ocally **A**dvanced **P**ancreatic cancer with **P**ercutaneous **I**rreversible **E**lectroporation (IRE) following initial systemic chemotherapy: a feasibility randomised controlled trial<br>**Study ID:** _________________<br>**NHS Number** _____________________

**Name of Researcher: ____**	
**Please initial each box**	
**1. I confirm that I have read and understand the patient information sheet date: ………………….. (Version: ....) describing the above study and have had the opportunity to consider the information, ask questions and have had these answered to my satisfaction.**	

For peer review only - http://bmjopen.bmj.com/site/about/guidelines.xhtml

**2. I understand that my participation in this study is voluntary and that I am free to withdraw at any time without giving a reason, without my medical care or legal rights being affected.**	
**3. I understand that sections of my medical notes and data collected during the study may be looked at by responsible individuals involved in this research at my hospital, the University of Liverpool, or from regulatory authorities where it is relevant to my taking part in research. I give permission for these individuals to have access to my records.**	

For peer review only - http://bmjopen.bmj.com/site/about/guidelines.xhtml

**4. I understand that imaging (Xray's, CT scans etc.) and blood test results will be reviewed by a central research team in order to assess success of the treatment I receive.**	
**5. I agree to allow my General Practitioner and any other relevant medical practitioner to be informed of my involvement in the study.**	

For peer review only

For peer review only - http://bmjopen.bmj.com/site/about/guidelines.xhtml

**6. I am aware that there is a qualitative aspect to this study and that patients may be selected at random to discuss their involvement and understanding of the study with a dedicated qualitative researcher. I agree to take part in these interviews if selected.**	
**7. I agree for a copy of this completed consent form to be sent to the University of Liverpool Clinical Trials Centre (where it will be kept in a secure location), to allow confirmation that my consent for the trial has been given.**	

For peer review only

For peer review only - http://bmjopen.bmj.com/site/about/guidelines.xhtml

**8. I understand that information held by the NHS and records maintained by the NHS Information Centre may be used to keep in touch with me and follow-up my health status.**	
**9. I agree that data from the study can be used in future research.**<br><br>10. I am aware that once registered on to the study there is no way to predict or influence which arm I am randomised to.	
11. I agree to take part in the LAP-PIE study	

**This part of the trial is OPTIONAL. Only initial YES if you wish to give permission. Initial NO if you do not give permission.**		
• I give permission for the samples of my blood/urine/tissue specimens to be taken and used for this trial and be transferred to research institutes in the UK and the rest of the world.	**Yes**	**No**
• I give permission for the samples of my blood/urine/tissue specimens to be used in future research and be transferred to research institutes in the UK and the rest of the world.	**Yes**	**No**
• I give permission for my stored pathological specimens to be used for this trial and be transferred to research institutes in the UK and the rest of the world.	**Yes**	**No**

For peer review only - http://bmjopen.bmj.com/site/about/guidelines.xhtml

• I give permission for my stored pathological specimens to be used for future research and be transferred to research institutes in the UK and the rest of the world.	**Yes**	**No**
• I give permission for genetic material (DNA and RNA) to be extracted from blood and cancer tissue specimens for current ethically approved research.	**Yes**	**No**
• I give permission for genetic material (DNA and RNA) to be extracted from blood and cancer tissue specimens for future ethically approved research.	**Yes**	**No**
• I agree that data from the study can be sent outside the European Economic Area (EEA).	**Yes**	**No**

**Name of patient**		**Date**		**Signature**
**Researcher**		**Date**		**Signature**
**Name of person taking consent (if different from researcher)**		**Date**		**Signature**

Please ensure the dates of consent are the same date for everyone, this form needs to be signed by both the patient and the person obtaining consent.
If there are any mistake made that need to be corrected, please draw a line through and write the correction next to it; initial and date the correction.
One copy of this form is given to the patient, one kept in the Patient File at the Research Site, and one copy is sent to the LCTC

For peer review only

For peer review only - http://bmjopen.bmj.com/site/about/guidelines.xhtml

PATIENT INFORMATION SHEET

Treatment of unresectable **L**ocally **A**dvanced **P**ancreatic cancer with **P**ercutaneous **I**rreversible **E**lectroporation (IRE) following initial systemic chemotherapy: a feasibility randomised controlled trial

You have been invited to take part in a research study. Before you decide, it is important that you understand why the research is being done and what it will involve. Please take time to read the following information carefully and discuss it with others if you wish.

- **Part One** tells you the purpose of the study and what will happen to you if you take part.
- **Part Two** gives you more detailed information about the conduct of the study.

The clinical team in change of your care will go through the details with you, but please ask us if there is anything that is not clear or if you would like more information. Take time to decide whether or not you wish to take part.
Thank you for reading this.

Part One

Please note that Locally Advanced Pancreatic Cancer has been shortened to LAPC throughout this information sheet.

What is the purpose of the study?

The Purpose of the LAP-PIE study is to see how well we are able to recruit patients to a study of this type, and to determine if a new type of cancer treatment is effective in reducing the size of a Pancreatic tumour to the point that it can be surgically removed. The treatment is known as Irreversible Electroporation (IRE), which essentially uses electric pulses to destroy the cancer cells whilst leaving the healthy cells unharmed. We are recruiting patients in order to see how effective this treatment option is compared to standard chemotherapy in patients, and at the same time look at issues in recruitment that will help us design a larger, more long-term study with the same goals.

Why have I been chosen?

You have been chosen as you are a patient who has been diagnosed with advanced pancreatic cancer that is not yet suitable for surgery. In addition, you are either due to start FOLFIRINOX® chemotherapy through the NHS, are currently having it, or at the end of treatment. You were approached by a member of the study team in a hospital that is currently running the LAP-PIE study and have been identified to meet some of our main inclusion criteria.

Do I have to take part?

For peer review only - http://bmjopen.bmj.com/site/about/guidelines.xhtml

No. It is up to you to decide whether or not to take part. If you do agree you will be asked to sign a consent form, but you are free to withdraw at any time afterwards without giving a reason. A decision to withdraw at any time, or a decision not to take part, will not affect the standard of care you receive.

What will happen to me if I take part?

From Randomisation, you will be on study for however long your second line chemotherapy lasts and a further 12 months for follow up.
The visits that you will attend are those that you would normally be required to make for treatment of LAPC; They may last slightly longer for some additional tests and for the research team to record the results of assessments for the study.
The results of standard of care tests such as blood, CT scans and biopsies will also be collected for the purposes of the study. All data is anonymised and kept on a secure system at the Liverpool Clinical Trials Centre.
If you are admitted to hospital for any reason during your time on the study, the details of the visit will be recorded and reviewed by the study team to ensure it is not related to the study itself.
At each visit you will be asked if you have experienced any adverse events or conditions or if you are taking any new medication since the last time you were seen.

What do I have to do?

If you become a patient on this study then you will be first asked to sign a consent form confirming you have read this form and spoken to the research team about the study and are willing to take part. Giving consent to the study means that you understand the assessments and visits necessary and are willing to adhere to the study protocol. You will then have some assessments done to confirm that you are eligible, many of them are tests you will already be having as a part of your standard care. You will have blood taken for laboratory assessment and will have an X-Ray of your chest and CT scan of your abdomen, pelvis and chest. All patients of childbearing potential will be given a test to confirm they are not pregnant before starting the study. There is also a quality of life questionnaire to complete which will ask you to record how you are feeling and describe your overall health.

Some patients will be selected for interviews with a qualitative researcher, which will be a discussion of your understanding of the study and how it was communicated to you. The interviewer will also ask you about your knowledge of the treatment available and your condition. This interview may be done over the phone, or in person during one of your visits to the hospital you are being treated at.

The results of these test will be sent to the LCTC who are running the study who will randomised you using a computer system to one of two arms, both arms are receiving the same standard of care that would be given, however one arm will receive on IRE procedure (more on this below).

For peer review only - http://bmjopen.bmj.com/site/about/guidelines.xhtml

Following randomisation, you will need to attend hospital to receive the therapy that your local oncologist recommends and adhere to all of the test and visits related to it. The choice of chemotherapy that you will be given is the same that would have been chosen regardless of whether you were on this study. Data on the tests and visits you attend will be recorded by the research team and given to the LCTC for the study results. Half of the patients randomised will also receive on IRE procedure at their recruiting hospital trust.

Patients receiving the IRE will be admitted to hospital for a few days during their second line chemotherapy where they will be given the IRE procedure. IRE will involve some pre procedure assessments and some after care in hospital. More details on the procedure are below.

At the end of chemotherapy, you will be reviewed to see if your LAPC is now suitable for surgery. If suitable you will be given the same procedure in hospital as normal for patients with LAPC.

Following the surgery and after caring you will need to attend hospital every 3 months for one year. At these visits you will have bloods taken, a physical exam and there will be X-Rays and CT scans done. You will also be given a quality of life questionnaire to complete at these visits.

Should you be unsuitable for IRE before you are meant to have it, or if your LAPC is not suitable for surgery after finishing chemotherapy, or your oncologist decides to take you off of the chemotherapy for whatever reason, you will still be given the option to remain on follow up for 12 months.

At the end of your involvement with the study, you will be given some final assessments and will not have to attend for any LAP-PIE related assessment, and no further data will be collected about you. Your involvement ends at the last follow up visit, or when you choose to not take part any further.

What is the Treatment that is being tested?

Irreversible Electroporation is a fairly new device, though has been licensed for use on the NHS and in patients with Locally Advanced Pancreatic Cancer. IRE is only routinely available on the NHS for patients with this disease at certain hospitals in the UK, such as the hospital you are attending.

IRE is delivered by inserting long needle probes through the skin and into the pancreas, the ends of them will surround the tumour. Once in place the generator on the IRE device will send electrical impulses down the needles and around the cancerous tissue. The pulses heat the cells and weaken their cell membranes, so they break down over around a 24-hour period following the treatment. IRE has been used previously in treating a number of types of cancer, such as Liver, Pancreatic and Prostate cancers on an experimental basis. IRE is used in order to reduce the size of a tumour so that it is

For peer review only - http://bmjopen.bmj.com/site/about/guidelines.xhtml

For peer review only

more suitable for surgical removal. It is different from other similar treatments in that is does not generate heat, which can cause damage to surrounding tissue.

The needles will be guided using a CT scan, and not open surgery, meaning there will be less recovery time. There will only be one 2-hour long procedure involving IRE for patients in this study. Before the procedure you will have a blood test and a further test 6 hours after your procedure ends. You will be admitted to hospital for a few nights for monitoring until deemed suitable for discharge, you will likely be prescribed pain killers to take as well. 6 days after your IRE treatment you will be asked to return for another blood test.

As IRE uses electricity, a heart monitor will be used to time each pulse, so it does not affect your hearts rhythm. Patients who have pacemakers or other indwelling electrical devices are therefore not suitable for this study.

All patients who take part in this study will still receive chemotherapy as normally indicated for their care, prior to receiving either chemotherapy plus IRE, or just more chemotherapy. It is not known at this time, whether the combination of IRE and Chemotherapy, or just Chemotherapy is more effective at reducing tumour size in LAPC to make it suitable for surgery. This study aims to look at how easy or difficult it is to recruit patients such as yourself and randomise to one option or the other before pancreatic surgery. The side effects, and overall quality of life of patients across the two groups will be compared too. The findings will guide a large study that will compare the two options with greater accuracy.

What are the alternatives for treatment?

Advanced Pancreatic Cancer that is not suitable for surgery is currently treated on the NHS using: Chemotherapy, Chemotherapy and Radiotherapy, or with Stereotactic body radiotherapy (essentially a more targeted, precise way of delivering radiotherapy). Each of these methods have their own levels of efficacy and side effects and given as needed depending on other patient factors such as overall health and the degree of Pancreatic Cancer.
All chemotherapy drugs have their own side effects, mostly nausea and fatigue as well as affecting your appetite. Radiotherapy is also known to carry these side effects and can also cause skin reactions in some cases. Some other devices are available that use direct heat to destroy cancerous tissue, however the temperatures are far greater than those used in IRE and can cause damage to the healthy surrounding tissue.

Your doctor will be able to give you more detail on these treatments.

What are the side effects of the treatment I will receive when taking part?

Patients who are enrolled to this study will receive, or have received, chemotherapy (Folfirinox®) for 6 weeks, before being randomised to Chemotherapy plus IRE, or just

For peer review only - http://bmjopen.bmj.com/site/about/guidelines.xhtml

chemotherapy. The side effects of the Folfirinox and whichever NICE approved chemotherapy given after will be well known and described to you by your oncologist.

The Side effects of the IRE based on early studies are:

- Some pain, though this can be managed with painkillers and will not normally last more than a few days
- Possible pancreatitis; this is an inflammation of the pancreas and can usually be managed with pain relief and you may need to spend a few days in hospital. If more severe this stay in hospital could extend to a few weeks or months, though this is not common
- Some problems with eating or drinking; you could have a diminished appetite or feel sick, or possibly experience heartburn. These symptoms do not normally last long
- A blood clot could develop in a vein near where the IRE was performed. This is rare and can be treated with medication, though would mean you may not be able to have surgery after the IRE, this will be for the doctor at site to decide. Standard practice with IRE is to administer drugs that reduce the risk of clots forming.
- In some very rare cases there may be some leaking of fluid from the bile duct or duodenum (the start of the small intestine). This can be caused by the needles that are used in IRE damaging these areas.

What are the possible disadvantages and risks of taking part?

This study is only recruiting patients who have been given Folfirinox as first line therapy and then their oncologist's choice for second line treatment. Therefore, the side effects and disadvantages of the chemotherapy aspect are the same as they would be regardless of entering the study. The potential side effects and risks associated with IRE are also listed above. It is possible that after undergoing either treatment option then your LAPC will still not be suitable for surgery. There is also always the possibility with any standard of care treatment that side effects, or complications, mean you will have to stop the therapy and another option will have to be considered.

There will also be the need to attend clinic on certain days that cannot be planned out at this stage, which will most likely be during working hours. There will also be more questions about your health and wellbeing asked at each routine visit in order to record the study data. Though most of the tests you will have are already part of standard of care, there will be some extra blood samples taken at various points in the study; all effort will be made to have these done at the same time as the routine bloods for your convenience.

What are the possible benefits of taking part?

We hope that the treatments will help you. However, this cannot be guaranteed. The information we get from this study may help us to improve the future treatment of patients with Advanced Pancreatic Cancer. The results of this feasibility study will also

For peer review only - http://bmjopen.bmj.com/site/about/guidelines.xhtml

help to develop a larger clinical trial into the use of IRE in patients with the same condition.

What happens when the research study stops?
It should be made clear if participants will have continued access to any benefits or interventions obtained during the research. If treatment is unavailable after the trial finishes, this should be explained, and information given on what treatment is available.

The chemotherapy that you will be given from this study are already approved and supplied via standard of care, and therefore will still be available should the study be terminated for any reason at your hospital.

What if there is a problem?
Any complaint about the way you have been dealt with during the study or any possible harm you might suffer will be addressed. The detailed information is given in Part 2 of this information sheet.

Will my taking part in this study be kept confidential?
Yes. All the confidential information about your participation in this study will be kept confidential. The detailed information on this is given in Part 2.

Contact for Further Information
Should you have any further queries regarding this study or about any of the treatments described above,

Please contact:
Appropriate job title:
[Trust to insert contact details]
Contact Number is:
[Trust to insert contact details]

This completes Part One of the Information Sheet. If the information in Part One has interested you and you are considering participation, please continue to read the additional information in Part Two before making any decision.

Part Two

What if new information becomes available?
Sometimes during the course of a research project, new information becomes available about the treatment/drug that is being studied. If this happens, your doctor will tell you about it and discuss with you whether you want to or should continue in the study. If you decide to withdraw your doctor will make arrangements for your care to continue. If you decide to continue in the study you will be asked to sign an updated consent form.

For peer review only - http://bmjopen.bmj.com/site/about/guidelines.xhtml

On receiving new information your doctor might consider it to be in your best interests to withdraw you from the study. He/she will explain the reasons and arrange for your care to continue.
If the study is stopped for any other reason you will be told why, and your continuing care will be arranged.

What will happen if I don't want to carry on with the study?

You retain the right to decide how much of the study you wish to participate in at all times. If there is any part of it you are not happy with you can choose not to have any test, give blood sample or answer any question. If you wish to no longer continue on the study this is your decision and will be respected. If you wish to stop during the chemotherapy phase then you will not be randomised to receive the IRE and may discuss with your doctor what you wish to do next. In all cases data and samples taken for patients who are withdrawn will be kept so they can still be used to help the study. At any time, you may tell the research team that you want any of your data and samples destroyed or not used in the research.

If you do not wish to attend the follow up visits this is entirely up to you. Every effort will be made to still contact you, or your GP for some data, or you may even decide that you no longer wish to be involved in the study at all.

What if there is a problem?
If you have a concern about any aspect of this study, you should ask to speak with the researchers who will do their best to answer your questions. If you remain unhappy and wish to complain formally, you can do this through the normal NHS Complaints Procedure. Details can be obtained from the hospital.
If you are harmed by taking part in this research project and this is due to someone's negligence, then you may have grounds for a legal action for compensation against the NHS Trust where you are being treated, but you may have to pay for your legal costs. The normal National Health Service complaints mechanisms should be available to you (if appropriate).

If you suffer harm arising from the negligent management or negligent design of the research you will have grounds to claim compensation. In the absence of negligence compensation is not payable.
If you experience non-negligent harm arising from the management or design of the research then The University of Liverpool clinical trials insurance may apply for this investigator-initiated study.
In the event of defective product then you may have grounds for a legal action for compensation against the manufacturer, but you may have to pay for your legal costs.

Will my taking part in this study be kept confidential?
The Royal Free Hospital is the sponsor for this study based in the United Kingdom. We will be using information from you and your medical records in order to undertake this study and will act as the data controller for this study. This means that we are

For peer review only - http://bmjopen.bmj.com/site/about/guidelines.xhtml

responsible for looking after your information and using it properly. [Sponsor organisation] will keep identifiable information about you [for x years after the study has finished/ until x].

Your rights to access, change or move your information are limited, as we need to manage your information in specific ways in order for the research to be reliable and accurate. If you withdraw from the study, we will keep the information about you that we have already obtained. To safeguard your rights, we will use the minimum personally identifiable information possible.

If you join the study, some parts of your medical records and the data collected for the study will be looked at by authorised persons from Cancer Research UK or their collaborators who are also involved in organising this research project and may be used in a number of research projects and/or for research related to the development of pharmaceutical products, diagnostics or medical aids by other scientists and research organisations within and outside the European Economic Area, in universities, the NHS or commercial companies involved in medical research worldwide which may also involve discussions with regulatory authorities. Patients' safety data may be reviewed outside the EU, but no one will be able to identify you. Data may also be looked at by representatives of regulatory authorities and by authorised people from the Trust, other NHS bodies or the commercial companies referred to above to check that the study is being carried out correctly. All will have a duty of confidentiality to you as a research participant.

A copy of your completed consent form will be sent to the Liverpool Cancer Trial Unit to allow them to check you have agreed to the trial. This will be kept in a secure location away from all the study data.

Involvement of the General Practitioner/Family Doctor (GP)
With your consent, your GP will be informed of your involvement in the trial. Any other medical practitioners who treat you, e.g. should you be admitted to hospital for any reason, will also be informed.

What will happen to any samples I give?
Some of your samples will be analysed at your hospital for the purpose of your treatment.
With your permission,. The researchers at the University of Liverpool work closely with other scientists in the UK and around Europe and, with your permission, your samples may be transferred to these research collaborators for use in future scientific studies. These samples will be used only for investigating Locally Advance Pancreatic Cancer and will not be used for any commercial purposes.
The samples will be kept in a secure place until we need them; nobody outside of the study will have access to **any** confidential information that you give to us. Confidential

For peer review only - http://bmjopen.bmj.com/site/about/guidelines.xhtml

details (such as your name, address and GP details) will be kept locally and not made available to collaborators.
Your sample will be coded and the researchers carrying out tests on the samples will not be given information they do not need to carry out the tests and analyse the results. Coded is not the same as anonymous. It will be possible to use the codes to identify that a result is from your sample. However, we do not plan to do this unless there is a good reason to do so. We will maintain this information so that we can properly manage the samples donated. For instance, sometimes we may need to update our record of your clinical details to help us interpret the results of tests.

What will happen to the results of the research study?
It is intended that once the study is complete a report will be written, and the results will be published to make them available to the public. They may also be used to apply to the regulatory authorities to make the drug widely available and/or for research related to the development of pharmaceutical products, diagnostics or medical aids. You will not be named or identified in any publication.

What rights do I have to the results of the research?
Any information derived directly or indirectly from this research, as well as any patents, diagnostic tests, drugs, or biological products developed directly or indirectly as a
result of this research may be used for commercial purposes. You have no right to this property or to any share of the profits that may be earned directly or indirectly as a result of this research. However, in signing this form and donating tissue and/or blood samples for this research, you do not give up any rights that you would otherwise have as a participant in research.

Who is organising and funding the research?

The study is funded by the NIHR and the equipment and training needed for IRE is being provided by Angiodynamics, the company who developed the technology.

Your doctor will not receive any payment for including you in this study.
The study is funded by the NIHR, the governing body that oversees and supports research done through the NHS. The money given a grant from them is to support the cost of performing the research such as the assessments, set up costs and time needed working on the study. No one involved in the research receives any payment for being involved, nor incentive to encourage recruitment to the LAP-PIE study.

Who has reviewed the study?
The study has been reviewed for scientific content by members of London Brent Research Ethics Committee (REC) has for ethical considerations and has a favourable opinion.
Thank you for taking the time to read and consider this information sheet. Should you decide to take part in the study, you will be given a copy of the information sheet and a signed consent form to keep.

For peer review only - http://bmjopen.bmj.com/site/about/guidelines.xhtml

For peer review only

For peer review only - http://bmjopen.bmj.com/site/about/guidelines.xhtml

BMJ Open

Treatment of unresectable Locally Advanced Pancreatic cancer with Percutaneous Irreversible Electroporation (IRE) following initial systemic chemotherapy (LAP-PIE) trial: Study protocol for a feasibility randomised controlled trial.

Journal:	*BMJ Open*
Manuscript ID	bmjopen-2021-050166.R2
Article Type:	Protocol
Date Submitted by the Author:	25-Nov-2021
Complete List of Authors:	Rai, Zainab; University College London, Division of Surgery and Interventional Science; Wellcome / EPSRC Centre for Interventional and Surgical Sciences (WEISS),<br>Ranieri, Veronica; Tavistock and Portman NHS Foundation Trust, Research & Development; University College London, Science & Technology Studies<br>Palmer, D. H.; University of Liverpool<br>Littler, Peter; Freeman Hospital<br>Ghaneh, Pauleh; University of Liverpool<br>Gurusamy, Kurinchi; University College London,<br>Manas, Derek; Newcastle Upon Tyne Hospitals NHS Trust<br>Pizzo, Elena; University College London, Applied Health Research<br>Psarelli, Eftychia; University of Liverpool, Liverpool Clinical Trials Centre<br>Gilmore, Roopinder; Royal Free London NHS Foundation Trust, Oncology; University College London<br>Peddu, Praveen; King's College Hospital NHS Trust, Department of Radiology,<br>Bartlett, David; University Hospitals Birmingham NHS Foundation Trust<br>de Liguori Carino, Nicola; Manchester Royal Infirmary, Regional Hepato-Pancreato-Biliary Unit<br>Davidson, Brian; Royal Free London NHS Foundation Trust, HPB Surgery
**Primary Subject Heading**:	Oncology
Secondary Subject Heading:	Radiology and imaging, Evidence based practice, Surgery
Keywords:	Adult oncology < ONCOLOGY, SURGERY, Pancreatic surgery < SURGERY, Interventional radiology < RADIOLOGY & IMAGING, Clinical trials < THERAPEUTICS

SCHOLARONE™
Manuscripts

Author Declaration

Research Ethics

The trial protocol has received the favourable opinion of the Brent Research Ethics Committee (REC).

Competing interests

We wish to confirm that there are no known conflicts of interest associated with this publication and there has been no significant financial support for this work that could have influenced its outcome.

Consent for publication

Not applicable

Availability of Data and Material

Not applicable

Funding

The LAP-PIE trial is funded by the National Institute for Health Research (NIHR) under its Research for Patient Benefit (RfPB) Programme (PB-PG-1217-20042).

EP is supported by the National Institute for Health Research ARC North Thames. The views expressed in this publication are those of the author(s) and not necessarily those of the National Institute for Health Research or the Department of Health and Social Care.

Authorship

All listed authors meet the ICMJE criteria.   We attest that all authors contributed significantly to the creation of this manuscript, each having fulfilled criteria as established by the ICMJE.

We confirm that the manuscript has been read and approved by all named authors.

We confirm that the order of authors listed in the manuscript has been approved by all named authors.

Acknowledgement

This project is funded by the National Institute for Health Research (NIHR) under its Research for Patient Benefit (RfPB) Programme (PB-PG-1217-20042). The views expressed are those of the author(s) and not necessarily those of the NIHR or the Department of Health and Social Care.

Word count: 4517

For peer review only

Title

Treatment of unresectable **L**ocally **A**dvanced **P**ancreatic cancer with **P**ercutaneous **I**rreversible **E**lectroporation (IRE) following initial systemic chemotherapy (LAP-PIE) trial: Study protocol for a feasibility randomised controlled trial.

ZL Rai, V Ranieri, D Palmer, P Littler, P Ghaneh, K Gurusamy, D Manas, E Pizzo, EE Psarelli, Roopinder Gilmore, Praveen Peddu, David C Bartlett, Nico de'Liguori Carino Davidson BR and the UK LAP-PIE study group

Corresponding author: ZL Rai email: Zainab.rai.19@ucl.ac.uk

Author Contributions

ZLR: manuscript preparation. VR: trial management. DP: protocol design, trial management and co-applicant. PL: protocol design, trial management and co-applicant. EEP: statistical analysis plan. KG: major contribution to design and funding application. DM: protocol design, trial management and co-applicant. PG: protocol design, trial management and co-applicant. EP: health economic analysis design. RG: protocol design, site lead, PP: protocol design, site lead, DB: site lead, NLC: site lead. BRD: principal investigator and grant holder, overall trial management, study design and review of manuscript. All authors read and approved the final manuscript.

Keywords

Locally advanced pancreatic cancer, Irreversible electroporation, Feasibility study, Randomised controlled trial, qualitative assessment

Abstract

Background

Approximately 30% of patients with pancreas cancer have unresectable locally advanced disease, which is currently treated with systemic chemotherapy. A new treatment option of Irreversible Electroporation (IRE) has been investigated for these patients since 2005. Cohort studies suggest that IRE confers a survival advantage, but with associated, procedure related complications. Selection bias may account for improved survival and there have been no prospective randomised trials evaluating the harms and benefits of therapy. The aim of this trial is to evaluate the feasibility of a randomised comparison of IRE therapy with chemotherapy versus chemotherapy alone in patients with locally advanced pancreatic cancer (LAPC).

Methods and analysis

Eligible patients with locally advanced pancreatic cancer who have undergone first line FOLFIRINOX chemotherapy will be randomised to receive either a single session of IRE followed by (if indicated) further chemotherapy or to chemotherapy alone (standard of care). Fifty patients from up to seven specialist pancreas centres in the UK will be recruited over a period of 15 months. Trial follow up will be 12 months. The primary outcome measure is ability to recruit. Secondary objectives include practicality and technical success of treatment, acceptability of treatment to patients and clinicians and safety of treatment. A qualitative study has been incorporated to evaluate the

patient and clinician perspective of the LAP-PIE trial. It is likely that the data obtained will guide the structure, the primary outcome measure, the power, and the duration of a subsequent multi-centre RCT aimed at establishing the clinical efficiency of pancreas IRE therapy. Indicative procedure related costings will be collected in this feasibility trial which will inform the cost evaluation in the subsequent study on efficiency.

Ethics and Dissemination

The protocol has received approval by London-Brent Research Ethics Committee reference number 21/LO/0077.

Results will be analysed following completion of trial recruitment and follow up. Results will be presented to international conferences with an interest in oncology, hepato-pancreatico-biliary surgery and interventional radiology and be published in a peer-reviewed journal.

Strengths and limitations of this study

- This trial is the first randomised comparison of IRE therapy with FOLFIRINOX versus FOLFIRINOX alone in patients with LAPC and will aim to evaluate the feasibility of recruitment of LAPC patients across multiple pancreas cancer specialist units in the UK.
- As a feasibility study, it is not powered to show effectiveness however it is likely that the data will help inform a larger multi-centre trial.
- Experience in optimising recruitment, site set up and developing local radiology expertise will be crucial for the success of the subsequent trial which will be powered from data collected during this feasibility study.
- A potential limitation of the study is inadequate recruitment due to competing trials recruiting patients with locally advanced pancreatic cancer.

For peer review only - http://bmjopen.bmj.com/site/about/guidelines.xhtml

Introduction

Pancreas cancer and LAPC

Pancreatic cancer (PC) is one of the deadliest cancers worldwide. It has a five-year survival rate of less than 6% and an increasing incidence (1). The low survival rate is mainly due to late diagnosis, as PC often remains asymptomatic until it becomes advanced. Surgery (pancreatic resection) is the only potentially curative

treatment. However, only 10 to 20% of patients are eligible for surgery (2). Approximately 30% of all patients have locally advanced pancreatic cancer (LAPC) at diagnosis and are not considered for surgical resection (3).

Chemotherapy for LAPC

Systemic chemotherapy is the standard treatment for unresectable pancreatic cancer (4). Despite advances in drug therapy for pancreas cancer over the last 20 years the overall survival (OS) and progression free survival (PFS) of these patients has not significantly improved (5). Furthermore, a meta-analysis reported that chemoradiation is not superior to chemotherapy alone in patients with LAPC, with comparable overall survival and higher rates of grade 3–4 adverse events (6).

In the UK NICE guidelines, published in 2018 recommend systemic combination chemotherapy for both locally unresectable and metastatic pancreatic cancer (7). NICE recommends first-line combination chemotherapy with 5-FluoroUracil, Leucovorin, Irinotecan and Oxaliplatin (FOLFIRINOX) in fit patients (Eastern Cooperative Oncology Group (ECOG) performance status of 0-1). In patients with co-morbidities and restricted activities, or those unable to tolerate this regime, NICE recommends gemcitabine alone or gemcitabine based therapy (6). Although FOLFIRINOX shows the best objective response in pancreatic cancer (8, 9) it is associated with more frequent and severe side effects, including neutropenia, diarrhoea and peripheral neuropathy (10).

Repeat staging after first line chemotherapy and possibility of resection

Staging is repeated following first line chemotherapy for LAPC and imaging is reviewed once again regarding suitability for resection. In patients with locally advanced disease undergoing first line treatment with FOLFIRINOX, 25.9%

For peer review only - http://bmjopen.bmj.com/site/about/guidelines.xhtml

underwent resection, with an R0 resection rate of 78.4%, however the long-term outcomes for these patients is not reported in the studies, and there is still a possibility of recurrence albeit at later date. (11).

IRE for LAPC

Irreversible electroporation is a novel ablative method for treating solid cancers (12). IRE treatment destroys cancer cells using electricity. It can be delivered via an open approach involving a laparotomy and placement of probes around the cancer or via a percutaneous approach which requires image guidance, via CT or ultrasound. Both methods require the patient to be under general anaesthesia with neuromuscular blockade. A single study using propensity score matching reported a median OS of 21.6 months in patients with LAPC treated with induction chemotherapy followed by IRE (13). A systematic review on pancreas IRE therapy from 2019 reported an OS from diagnosis or treatment of up to 27 months and a morbidity rate of 30% (14).

First Line, Up-front Chemotherapy

The timing of chemotherapy in relation to IRE therapy may influence outcome. Studies where IRE has been delivered before chemotherapy have reported only modest increases in median OS (15, 16) whereas a systematic review evaluating the impact of IRE after induction treatment reported an overall survival of 27 months (14). This may be because chemotherapy responders who have better outcomes are being selected to undergo IRE or alternatively it may suggest that chemotherapy modifies the tumour microenvironment making it more sensitive to IRE.

Second line chemotherapy for LAPC

For peer review only - http://bmjopen.bmj.com/site/about/guidelines.xhtml

NICE recommends that patients who have previously undergone FOLFIRINOX therapy as first line should be offered Gemcitabine based second-line chemotherapy if their cancer has progressed on FOLFIRIONOX (17).

Locally advanced and borderline unresectable LAPC

The definition of borderline resectable disease is controversial (18), whereas the definition of unresectable locally advanced disease has clear consensus, namely the involvement of the coeliac artery or encasement of the superior mesenteric artery of more that 180º and or the involvement of the superior mesenteric vein or portal vein with no distant metastasis (19). Therefore, the LAP-PIE trial will only involve patients with locally advanced disease fulfilling published criteria.

Complications of pancreas IRE therapy

Reported complications following pancreas IRE include pancreatic fistula, venous and arterial thrombosis, arterial pseudoaneurysm and pancreatic abscess (14). The mean morbidity rate in the studies examined is 34%, but can reach up to 62% (20) The average rate of severe complications (defined as greater than Grade III on the Clavien-Dindo scale) following IRE is 8% but can be as high as 44% (21). The average procedure related mortality rate in these studies is 3% (12).

There are significant variations in the percentage and severity of complications reported. This difference may be due to the case selection, whether IRE is carried out during surgery (via an open or laparoscopic approach) or with image-guidance in the Interventional Radiology (IR) suite, the treatment algorithm, the completeness of reporting from treatment centres or the experience of the radiologist and/or centre. Only two studies specifically mention the experience of clinicians delivering IRE (16,

For peer review only - http://bmjopen.bmj.com/site/about/guidelines.xhtml

22) and currently, there is limited published data available exploring the number of IRE procedures performed and the relationship with outcomes (23).

IRE is a palliative option and therefore its impact on patient's quality of life (QoL) is an essential consideration. A single-centre prospective study evaluating the QoL pre and post IRE in patients with LAPC demonstrated that IRE had no adverse effect on QoL in the short-term however, concomitant treatment with chemotherapy after IRE as well as other inter-related factors may confound the results (24). We were unable to find any studies comparing QoL changes following IRE to a control group. The published data to date, regarding the improvement in QoL in patients with LAPC undergoing IRE is weak and needs further exploration.

The current information available in the literature regarding the use of IRE in the treatment of LAPC is primarily from retrospective and prospective cohort studies. A single prospective randomised controlled trial has recently reported on the use of IRE in LAPC with concurrent gemcitabine therapy (25). The authors report a survival advantage in patients treated with IRE and gemcitabine. Therefore some evidence is available for the efficacy of IRE in LAPC however, the LAP-PIE trial will evaluate whether this is also the case in the modern context of FOLFIRINOX chemotherapy, a regimen recommended in the UK by NICE. The LAP-PIE trial is a feasibility trial to determine whether patients would be willing to have pancreas IRE treatment, whether they would accept randomisation to IRE or standard of care chemotherapy, whether they would comply with treatment and study data collection and to collect preliminary data on efficacy end points and indicative costs related to health resources, with which to design a subsequent trial on treatment efficiency and cost effectiveness.

For peer review only

Methods and analysis

Study design and setting

The LAP-PIE trial is a UK multi-centre feasibility study that will recruit 50 patients with LAPC in whom the disease has remained localised and unresectable following FOLFIRINOX. Patients will receive 3-6-months of FOLFIRINOX prior to restaging and being assessed for study eligibility. All participants will be offered palliative chemotherapy according to standard centre protocols and half will also be offered one IRE procedure. If new agents for second line therapy are approved by NICE prior to or during the LAP-PIE trial these can be offered in the second line chemotherapy arm and following IRE therapy if considered appropriate by the treating oncologist. Patients randomised to the control arm who have benefitted from initial chemotherapy with FOLFIRINOX may continue on the same regimen if this deemed appropriate by the treating oncologist. It is an open-label study. Patient enrolment is expected to last 15 months assuming a recruitment rate of 0.8 patients per site per month across 7 UK centres and an opening site rate of 0.5 per month. The trial will be conducted in specialist pancreas cancer centres. IRE will be delivered across all trial centres. Interventional radiologists performing pancreas IRE in the trial will have performed a minimum of 5 previous pancreas or liver IRE ablations prior to administrating IRE to participants enrolled in the trial. Additional technical support will be available from manufacturers of the equipment, AngioDynamics ®. Figure 1 summarises the trial design and Table 1 demonstrates the schedule of enrolment, interventions and assessments.

For peer review only

For peer review only - http://bmjopen.bmj.com/site/about/guidelines.xhtml

Participants

Adults (≥18 years) with LAPC following first line chemotherapy with FOLFIRINOX will be eligible. Only patients who have completed FOLFIRINOX as their first line chemotherapy will be eligible. Patients who have received FOLFIRINOX as second line therapy prior to inclusion will not be eligible. Following FOLFIRINOX chemotherapy patients will be re-staged with CT chest, abdomen and pelvis (CAP) +/- an FDG Positron Emission Tomography (PET) scan, depending on local site protocols. The imaging will be reviewed in a specialist multi-disciplinary team (sMDT) meeting.

Patients with borderline resectable disease will be excluded. The definition for this is summarised in Table 2. Other exclusion criteria include untreated biliary or gastric outlet obstruction, coagulopathy, chronic kidney disease (CKD) stage ≥ 3 (the criteria for CKD stage 3 is an estimated glomerular filtration rate of between 30 and 59) (26), indwelling electrical devices, abdominal varices precluding safe access to the pancreas and significant co-morbidities that would contraindicate general anaesthesia and neuromuscular blockade. A contraindication, as per manufacturers guidance, is epilepsy as the electrical discharges from IRE treatment may in theory result in seizure activity through pulsatile brain stimulation. Although the overall impact of IRE on seizure activity and neurological conditions remains unclear (27), in the interest of robust patient safety, patients with neurological conditions will be excluded. The full inclusion and exclusion criteria are summarised in Figure 2.

Figure 1: Trial flow diagram

sMDT; specialist multidisciplinary team, IRE; irreversible electroporation, SoC; standard of care, CTCAP; computed tomography chest abdomen pelvis, QoL quality of life, PFS; progression free survival, OS; overall survival.

Figure 2: Inclusion and exclusion criteria of the LAP-PIE trial

LAPC: Locally Advanced Pancreatic Cancer, FOLFIRINOX; 5-FluoroUracil, Leucovorin, Irinotecan and Oxaliplatin, sMDT; specialist multidisciplinary team, IRE; irreversible electroporation, IR; interventional radiologist, WHO; world health organisation, CKD; chronic kidney disease, Hb; haemoglobin

Table 1. Schedule of enrolment, interventions, and assessments of the LAP-PIE trial.

	Study Period									
	**Enrolment**	**Allocation**	**Post-Allocation Follow-up Time Points**							**Close-out**
**TIMEPOINT**	***-t₁***	**0**	***Pre-IRE***	***6 hours post-IRE***	***6 days post-IRE***	***3 months***	***6 months***	***9 months***	***12 months***	
**ENROLMENT:**										
**Eligibility screen**	X									
**PIS given**	X									
Systemic treatment with FOLFIRINOX	X									
sMDT	X									
Informed consent	X									
**Allocation**		X								
**INTERVENTIONS:**										
IRE (intervention)			◆—	—	—	—	—	—	—◆	
Systemic chemotherapy (SoC)						◆—	—	—	—◆	

For peer review only

For peer review only - http://bmjopen.bmj.com/site/about/guidelines.xhtml

**ASSESSMENTS:**										
CT CAP	X					X	X	X	X	
Blood tests	X		X	X	X	X	X	X	X	X
HRQoL						X	X	X	X	X
Complications						X	X	X	X	X

PIS patient information sheet, sMDT specialist multidisciplinary team, IRE irreversible electroporation, SoC standard of care, CT CAP computed tomography chest abdomen pelvis, HRQoL health related quality of life.

Table 2: Definition of borderline resectable pancreatic cancer (18)

	**Borderline Resectable**	**Unresectable**
**Celiac artery**	Tumour without encasement or abutment	Tumour contact > 180º
**Superior Mesenteric artery**	Tumour abutment </= 180º	Tumour contact > 180º
**Superior Mesenteric vein (SMV)/Portal vein (PV)**	Tumour abutment of SMV/PV > 180º or abutting</= 180º with irregularity of the vein ± thrombosis with anatomical structures that still permit safe and complete resection with vein reconstruction	SMV/PV unreconstructible secondary to tumour involvement or occlusion
**Common hepatic artery**	Reconstructible short segment abutment	Unreconstructible interface between tumour mass and vessel

Adapted from Callery MP, Chang KJ, Fishman EK et al. Pre-treatment assessment of resectable and borderline resectable cancer: expert consensus statement. Ann Surg Oncol 2009; 16:1727-33.

Randomisation

Following confirmation of eligibility randomisation will be performed by the Liverpool Clinical Trials Centre (LCTC) using a web-based randomisation procedure. The LCTC will inform local centres of the treatment arm and the local LAP-PIE Clinical Trials Nurse will inform the patient and make arrangements for clinical review and the date for the chosen therapy.

For peer review only - http://bmjopen.bmj.com/site/about/guidelines.xhtml

Intervention

Pancreas IRE

Patients will be invited for an anaesthetic pre-assessment to ensure they are fit enough to undergo IRE under general anaesthesia prior to being booked for hospital admission. Patients will be admitted to a ward or radiology admissions unit under joint clinical care of a pancreas interventional radiologist and one of the other site leads (Surgery, Oncology).

The CT CAP performed on completion of FOLFIRINOX will be used by the interventional radiologist to plan IRE therapy. Under general anaesthesia IRE will be delivered percutaneously using CT guidance. Timing of the procedure will be co-ordinated with the treating oncologist to ensure bone marrow recovery following FOLFIRINOX chemotherapy. A broad-spectrum antibiotic, based on individual centres first line prophylaxis for GI infection, will be given prior to the procedure and 6 hours and 12 hours following IRE.

The IRE procedure with be standardised to use between 2-6 IRE needles depending on the size, shape and relationship of critical structures to the tumour, with a maximum inter-electrode distance of 2cm. A treatment margin of at least 0.5cm will be maintained. The needle placement prior to treatment will be recorded on CT.

IRE therapy will be delivered using ECG synchronisation to ensure the pulse is delivered during diastole. The length of the electrical pulse and amperage will be decided by the treating interventional radiologist and will be between 70-100 microseconds with an amperage of between 20-40 AMPs. The treating interventional radiologist will have relevant expertise in IRE procedures with a minimum of 5 IRE procedures on liver or pancreas performed previously.

For peer review only

Following IRE therapy, full blood count, liver function tests, coagulation, renal function, and amylase (or lipase) will be monitored and patients will be admitted for a minimum of 24 hours to ensure adequate pain management and to monitor and manage any immediate complications. Participants will be discharged with pain relief as required and DVT prophylaxis for 4 weeks.

The intra-operative imaging will be reviewed by the lead radiologist at each site and if the entire tumour has been treated based on these images, the procedure will be deemed as successful. The technical success will be confirmed by central review. Contrast CT follow up scans will be assessed for evidence of tumour growth as indicated by mass enlargement or increased vascularity. In the absence of tumour growth, the procedure will be considered successful at follow up. At the end of the follow up period, serial imaging will be analysed, and the tumour staged. Local disease control will be evaluated according to the Response Evaluation Criteria in Solid Tumours (RECIST). This will be performed by the radiology leads at each site and confirmed by a central review.

Following IRE, patients in this arm will be offered second line chemotherapy, which is the current standard of care. The drug choice and timing of chemotherapy will be decided by treating oncologists and will adhere to NICE guidelines.

Controls (Second line systemic chemotherapy)

Patients randomised to receive further chemotherapy (chemotherapy alone) will commence treatment within 6 weeks after sMDT or 4 weeks after randomisation, whichever is sooner. The second line chemotherapy regime will be selected at the discretion of the treating oncologist and will adhere to NICE guidelines and local protocols.

Outcomes

For peer review only

The primary objective of the LAP-PIE trial is to ascertain feasibility of such a trial concept. In particular, the ability to identify and recruit LAPC patients at the selected sites to the LAP-PIE trial will be evaluated. The primary outcome consists of recruitment rate, number of screening failures, number of patients completing study pathway as per protocol and trial withdrawal rate.

The secondary objectives of the LAP-PIE trial aim to assess the practicality and technical success rate of pancreas IRE treatment. Technical success will be determined by the interventional radiologist performing the procedure. The procedure will be deemed to be technically successful if the entire tumour has been treated based on imaging performed during the intervention. Completion and complication rates of IRE procedures will be evaluated. The proportion of patients in whom percutaneous IRE was successful, defined as no local growth on follow up, will be determined as will the proportion of patients who became eligible for surgery following IRE and the R0/R1 resection rates in these participants. Acceptability of pancreas IRE therapy to patients and their clinicians, rate of withdrawal from the study and outcome measures including overall survival (measured from randomisation), progression free survival and quality of life will be evaluated. Finally, health related costs, including indicative costs related to health resource use in both arms, social costs of attending IRE treatment such as cost of travel to and from a tertiary centre, time off work and social support costs will be evaluated.

Follow up

All patients will be clinically reviewed every 3 months during which a CT CAP will be performed. Additional imaging can be performed if clinically indicated. Other investigations will include biochemistry and haematology, CA19-9 levels, CRP, quality of life as measured by a validated questionnaire (EQ5D-5L) (28) and a clinical review

For peer review only

of any adverse events experienced. This information will be recorded in an electronic case report form (eCRF).

Sample size calculation

Formal sample size calculations are not appropriate for a feasibility study. Sample sizes between 24 and 50 have been recommended to estimate the standard deviation required for a sample size calculation to allow for the design of a subsequent randomised controlled trial aimed at evaluating the cost effectiveness of IRE in patients with LAPC (29, 30). A convenient sample size of 50 patients, randomised equally to the two groups, has been selected.

Patient and Public Involvement (PPI)

PPI representatives have been involved in designing the trial ensuring that patient interests are central to the study. The design of the trial was presented to the Pancreas Cancer Action Day which has a large representation from patients and carers and received extremely positive feedback. Two patient representatives will serve on the Trial management group (TMG) and will be involved in informing, monitoring, and helping to interpret the findings from the study. Both patient representatives were involved in the trial design and in developing the trial protocol and patient information sheets. As the trial progresses, they will help address any patient related concerns and will have input into the interpretation of the trial outcomes and how this will be communicated to the patient user groups. They will then be involved, along with other patient advocates, the trial management team and other key invited participants in the discussion of the trial findings at a focus group meeting and

For peer review only - http://bmjopen.bmj.com/site/about/guidelines.xhtml

the preparation of the protocol for a subsequent trial addressing efficacy and cost effectiveness of IRE therapy in pancreas cancer. Once the data analysis and interpretation has been completed they will contribute to the dissemination of the trial outcome information to patients support groups via social media.

Assessment of feasibility

One aspect of the feasibility evaluation of this trial is to evaluate the ability to recruit LAPC patients into a trial of this nature. The average number of patients with unresectable locally advanced pancreatic cancer within UK specialist pancreas units is 60 patients per centre per year (Royal Free Hospital London audit data 2017, unpublished). Staging following FOLFIRINOX chemotherapy will demonstrate localised but unresectable disease in approximately 40 out of 60 patients (65%) (12). These patients will be eligible for the LAP-PIE trial. We anticipate recruiting 20% of potential participants to the LAP-PIE trial which equates to 8 per centre per year. Recruitment within 5 centres would allow 40 UK patients per year. A recruitment time of 15 months would allow 50 patients to be recruited.

Assessment of Complications

Adverse Events (AE) for the Standard of Care Chemotherapy will be defined using the Common Terminology Criteria for Adverse Events (CTCAE) Version 4.0 (31). AEs for IRE will be defined using the Clavien Dindo classification of surgical complications (32). Complications greater than grade 3 on the CTCAE scale and greater than grade III on the Clavien Dindo scale will be reported.

A Serious Adverse Event (SAE) for patients randomised to receive Standard of Care Chemotherapy only will be compared against the reference safety information (RSI) within the current summary of product characteristics (SmPC) for that drug. The RSI will be taken from the latest SmPC available for the drug on

For peer review only - http://bmjopen.bmj.com/site/about/guidelines.xhtml

https://www.medicines.org.uk/emc. The RSI for IRE will be from the Nanoknife system users' manual.

Suspected Unexpected Serious Adverse Reaction (SUSAR) will be defined as life threatening, serious and non-recognised complications. 2 or more reports of SUSARs related to IRE therapy will result in the cessation of trial activity in all centres to allow for a thorough investigation into the safety of IRE. Any premature halt in the trial will be reported to the Medicines and Healthcare products Regulatory Agency (MHRA) and the Regional Ethics Committee (REC).

Qualitative Study in LAP-PIE trial

A qualitative study will be carried out to understand patients' and clinicians' perspectives and experiences of the procedure and the design of the trial. All patients approached to participate in the trial will be invited to participate. A purposive sample of healthcare professionals involved in the trial will also be invited. Trial nurses, local R&D (research and development) personnel and Network CRN (clinical research network) managers will be included. This information will be gathered via interviews by an experienced qualitative researcher.

Interviews will be transcribed verbatim and managed using computer-assisted qualitative data analysis software (iNVivo) (33). Emerging data will be used to modify trial information and protocol. This may include the development of training materials for staff and changes to participant information sheets.

Translational Study

A translational sub study will evaluate prognostic markers for outcome. Imaging prior to and during IRE therapy will be correlated with outcome as will the immune response to IRE therapy by assessing circulating immune responses (including

For peer review only - http://bmjopen.bmj.com/site/about/guidelines.xhtml

absolute lymphocyte count, eosinophils, neutrophil: leucocyte ratio, Tregs and IFNγ) at baseline, 6 hours and 6 days post therapy.

Progress to full trial

The criteria to progress to a subsequent full trial will be determined quantitatively as (A) the ability to recruit 50 patients to the study across the study sites (B) a consent rate of 20+/-11%, (C) overall technical success rate of 50+/-14% and (D) a loss to follow-up of 10+/-8%.

Ethics

The trial will abide by the principles of the World Medical Association Declaration of Helsinki and has approval from London Brent Research Ethics Committee (REC), reference number 21/LO/0077. The trial has been registered on ISRTCN (International Standard Randomised Controlled Trial Number) https://doi.org/10.1186/ISRCTN14986389

Publication and Dissemination

Results from different centres will be analysed and published together following completion of the recruitment and follow up period. This will be presented to an international conference with an interest in hepato-pancreaticobiliary surgery, oncology and interventional radiology and published in a peer- reviewed journal.

For peer review only - http://bmjopen.bmj.com/site/about/guidelines.xhtml

For peer review only

Discussion

Trial justification

Local ablative therapy using IRE in patients with LAPC has been shown to be beneficial in prospective and retrospective studies (median survival 27 months) (14), however the increase in survival reported may be due to patient selection for IRE rather than the effect of IRE (selection bias). A systematic review of IRE therapy in LAPC included retrospective and prospective studies but no randomised controlled trials (RCT) (14, 34). Moreover, the heterogeneity among individual trial participants including varying concomitant treatments prevented combination of data sets making it difficult to establish the true impact of IRE. Despite this, the systematic review is a useful aid in summarising findings including complication rates following IRE and highlighting the current knowledge gaps such as the relationship between number of procedures performed and outcome and the impact of IRE on quality of life.

A study using propensity score matching (PSM) reported a median OS of LAPC patients undergoing IRE of 21 months (13), similar to that reported by the systematic review (27 months) (14). Such a study may effectively adjust for observed variables between cohorts and can contribute to a more precise estimation of treatment response. However, selection bias cannot be completely eliminated because PSM does not adjust for unobserved differences between groups. In order to address selection bias, a well-designed randomised control trial is required.

Before IRE can be justified by NICE to be incorporated into routine clinical practice, a randomised control trial is needed evaluating the benefit of IRE in addition to standard of care chemotherapy, which in the UK is FOLFIRINOX . Whilst a larger trial is required to investigate IRE treatment in locally advanced pancreatic cancer,

For peer review only - http://bmjopen.bmj.com/site/about/guidelines.xhtml

patients and clinicians may not be willing to enter the trial and therefore currently a large RCT is justified but not feasible.

Importance of doing a feasibility study

Clinical trials comparing different treatment types have high rates of failure (35). This is particularly true where there has been a lack of feasibility testing and qualitative evaluation of the study design (26,27). Furthermore, if a feasibility study demonstrates that a full trial would be unlikely to meet the required patient recruitment rate, intervention acceptability and other feasibility outcomes then the potential wasted cost of a larger clinical trial is avoided making feasibility trials an important first step (36). A feasibility trial will enable a realistic assessment of the capability of registered sites in patient enrolment and will compare IRE to the current standard of care. The qualitative study will explore the patient evaluation of information provided, the quality and clarity of this information, the individual providing the information, the clinical equipoise, support and attitude of other clinicians and general practitioners and the views and experiences of patients' family and friends. The feasibility trial will provide information about challenges that may be encountered during enrolment and these factors can be mitigated for if a larger RCT is justified and subsequently planned.

Exclusion of borderline resectable disease

Patients with borderline resectable disease have been excluded from the LAP-PIE trial. The exact definition of borderline resectable pancreatic disease (BRPD) varies, but broadly it is characterised as tumour abutting or involving vascular structures (18). A number of studies have suggested that neoadjuvant chemotherapy or chemoradiotherapy may downsize the tumour allowing resection and preventing local recurrence (37-39). The LAP-PIE trial will exclude patients with BRPD because of the variability in radiological criteria used for BRPD and to avoid patients being

For peer review only - http://bmjopen.bmj.com/site/about/guidelines.xhtml

recruited to the trial who would be considered in some pancreas units for surgical intervention.

Choice of FOLFIRINOX as first line

Previous studies have reported no significant improvement in survival when IRE is used as a first line treatment for unresectable LAPC (15, 16). Furthermore, systemic chemotherapy has previously been shown to downgrade a proportion of locally advanced unresectable cancer into resectable disease and therefore delivering IRE first line may result in an unnecessary, invasive procedure in a group of patients whose tumours may be downgraded and who would go on to have potentially curative resectional surgery.

FOLFIRINOX was specifically selected as the regime of choice because it has been shown to result in the greatest number of patients downgraded to resectable disease (10). Therefore, the patients recruited into the LAP-PIE trial will be those with truly unresectable disease in whom the impact of IRE can be evaluated.

Translational studies

The translational study will determine the radiological and immune response of patients undergoing IRE and determine its biological significance with emphasis on overall survival and disease response. This data will provide valuable information about the type of patient that is likely to benefit the most from IRE and therefore form the basis of a management design that is tailor made to individual patients.

Mandated imaging in the LAP-PIE trial is a CT scan and this will provide information about contrast enhancement features. The images will be reviewed and information about tumour characteristics such as size, histology and grade will be collected. The relationship between treatment parameters and imaging findings during the IRE procedure, as well as pre-treatment PET scans, will be analysed. The data

For peer review only

generated will be used to develop a mathematical model to predict IRE responses and help understand the significance of radiological changes and their biological significance. Blood samples will be taken from patients enrolled in the study and analysed for peripheral immune response markers. This will include absolute lymphocyte count and eosinophils, neutrophil: leucocyte ratio, Tregs and IFNγ. Previous studies have demonstrated a detectable peripheral immune response in patients following IRE (40, 41). Analysing the immune response of patients undergoing IRE will provide information about whether a peripheral immune response can be determined by simple biochemical tests in these patients and if this can predict treatment efficacy. This information may be used in the future to predict treatment response and guide future patient selection.

The qualitative sub-study will provide the opportunity to collate and analyse the experiences of both patients and clinicians in order to understand the perspectives and experiences of the procedure and trial providing useful information on the barriers and facilitators experienced by both groups.

Preliminary studies have shown that IRE in addition to chemotherapy may provide survival benefit in patients with locally advanced pancreatic cancer (14-16) (21-23) (34). However, this may be due to patient selection for IRE. There are no randomised controlled trials addressing the issue. The LAP-PIE trial is the first UK-based RCT of pancreas IRE in LAPC patients. The trial will assess feasibility and safety of delivering this intervention and if successful, will progress to a subsequent large scale RCT trial in assessing the efficacy and cost-effectiveness of IRE therapy in pancreatic cancer. This research is required to investigate whether IRE improves survival, health-related quality of life, or both.

For peer review only - http://bmjopen.bmj.com/site/about/guidelines.xhtml

References

1. Are C, Chowdhury S, Ahmad H, Ravipati A, Song T, Shrikandhe S, et al. Predictive global trends in the incidence and mortality of pancreatic cancer based on geographic location, socio-economic status, and demographic shift. J Surg Oncol. 2016;114(6):736-42.
2. Ghaneh P, Costello E, Neoptolemos JP. Biology and management of pancreatic cancer. Postgrad Med J. 2008;84(995):478-97.
3. Balaban EP, Mangu PB, Khorana AA, Shah MA, Mukherjee S, Crane CH, et al. Locally Advanced, Unresectable Pancreatic Cancer: American Society of Clinical Oncology Clinical Practice Guideline. J Clin Oncol. 2016;34(22):2654-68.
4. Ghosn M, Ibrahim T, Assi T, El Rassy E, Kourie HR, Kattan J. Dilemma of first line regimens in metastatic pancreatic adenocarcinoma. World J Gastroenterol. 2016;22(46):10124-30.
5. Rahib L, Fleshman JM, Matrisian LM, Berlin JD. Evaluation of Pancreatic Cancer Clinical Trials and Benchmarks for Clinically Meaningful Future Trials: A Systematic Review. JAMA Oncol. 2016;2(9):1209-16.
6. Wang C, Liu X, Wang X, Wang Y, Cha N. Effects of chemoradiotherapy and chemotherapy on survival of patients with locally advanced pancreatic cancer: A meta-analysis of randomized controlled trials. Medicine (Baltimore). 2018;97(36):e12260.
7. Excellence NIfHaC. Overview | Pancreatic cancer in adults: diagnosis and management | Guidance | NICE: NICE; 2020 [Available from: https://www.nice.org.uk/guidance/ng85.
8. Pavillard V, Formento P, Rostagno P, Formento JL, Fischel JL, Francoual M, et al. Combination of irinotecan (CPT11) and 5-fluorouracil with an analysis of cellular determinants of drug activity. Biochem Pharmacol. 1998;56(10):1315-22.

For peer review only - http://bmjopen.bmj.com/site/about/guidelines.xhtml

9. Ducreux M, Mitry E, Ould-Kaci M, Boige V, Seitz JF, Bugat R, et al. Randomized phase II study evaluating oxaliplatin alone, oxaliplatin combined with infusional 5-FU, and infusional 5-FU alone in advanced pancreatic carcinoma patients. Ann Oncol. 2004;15(3):467-73.
10. Suker M, Beumer BR, Sadot E, Marthey L, Faris JE, Mellon EA, et al. FOLFIRINOX for locally advanced pancreatic cancer: a systematic review and patient-level meta-analysis. Lancet Oncol. 2016;17(6):801-10.
11. Hajatdoost L, Sedaghat K, Walker EJ, Thomas J, Kosari S. Chemotherapy in Pancreatic Cancer: A Systematic Review. Medicina (Kaunas). 542018.
12. Hurt CN, Falk S, Crosby T, McDonald A, Ray R, Joseph G, et al. Long-term results and recurrence patterns from SCALOP: a phase II randomised trial of gemcitabine- or capecitabine-based chemoradiation for locally advanced pancreatic cancer. Br J Cancer. 2017;116(10):1264-70.
13. He C, Wang J, Sun S, Zhang Y, Lin X, Lao X, et al. Irreversible electroporation versus radiotherapy after induction chemotherapy on survival in patients with locally advanced pancreatic cancer: a propensity score analysis. BMC Cancer. 192019.
14. Lafranceschina S, Brunetti O, Delvecchio A, Conticchio M, Ammendola M, Currò G, et al. Systematic Review of Irreversible Electroporation Role in Management of Locally Advanced Pancreatic Cancer. Cancers (Basel). 112019.
15. Mansson C, Brahmstaedt R, Nygren P, Nilsson A, Urdzik J, Karlson BM. Percutaneous Irreversible Electroporation as First-line Treatment of Locally Advanced Pancreatic Cancer. Anticancer Res. 2019;39(5):2509-12.
16. Belfiore MP, Ronza FM, Romano F, Ianniello GP, De Lucia G, Gallo C, et al. Percutaneous CT-guided irreversible electroporation followed by chemotherapy as a novel neoadjuvant protocol in locally advanced pancreatic cancer: Our preliminary experience. Int J Surg. 2015;21 Suppl 1:S34-9.
17. Excellence NIfHaC. Recommendations | Pancreatic cancer in adults: diagnosis and management | Guidance | NICE: NICE; 2020 [Available from: https://www.nice.org.uk/guidance/ng85/chapter/Recommendations#managing-resectable-and-borderline-resectable-pancreatic-cancer.
18. Callery MP, Chang KJ, Fishman EK, Talamonti MS, William Traverso L, Linehan DC. Pretreatment assessment of resectable and borderline resectable pancreatic cancer: expert consensus statement. Ann Surg Oncol. 2009;16(7):1727-33.
19. Network NCC. Pancreatic Adenocarcinoma, Version 2.2017, NCCN Clinical Practice Guidelines in Oncology - NCCN-2017-pancreatic-cancer.pdf: NCCN; 2017 [Available from: http://clubedopancreas.pt/wp-content/uploads/2018/06/NCCN-2017-pancreatic-cancer.pdf.
20. Narayanan G, Hosein PJ, Beulaygue IC, Froud T, Scheffer HJ, Venkat SR, et al. Percutaneous Image-Guided Irreversible Electroporation for the Treatment of Unresectable, Locally Advanced Pancreatic Adenocarcinoma. J Vasc Interv Radiol. 2017;28(3):342-8.
21. Kluger MD, Epelboym I, Schrope BA, Mahendraraj K, Hecht EM, Susman J, et al. Single-Institution Experience with Irreversible Electroporation for T4 Pancreatic Cancer: First 50 Patients. Ann Surg Oncol. 2016;23(5):1736-43.
22. Liu S, Qin Z, Xu J, Zeng J, Chen J, Niu L, et al. Irreversible electroporation combined with chemotherapy for unresectable pancreatic carcinoma: a prospective cohort study. Onco Targets Ther. 2019;12:1341-50.

For peer review only

23. Philips P, Hays D, Martin RC. Irreversible electroporation ablation (IRE) of unresectable soft tissue tumors: learning curve evaluation in the first 150 patients treated. PloS one. 2013;8(11).
24. Field W, Rostas JW, Martin RCG. Quality of life assessment for patients undergoing irreversible electroporation (IRE) for treatment of locally advanced pancreatic cancer (LAPC). Am J Surg. 2019;218(3):571-8.
25. Ma YY, Leng Y, Xing YL, Li HM, Chen JB, Niu LZ. Gemcitabine plus concurrent irreversible electroporation vs gemcitabine alone for locally advanced pancreatic cancer. World journal of clinical cases. 2020;8(22).
26. Outcomes KDIG. KDIGO 2012 Clinical Practice Guidelines for Evaluation and Management of Chronic Kidney Disease 2013 [Available from: https://kdigo.org/wp-content/uploads/2017/02/KDIGO_2012_CKD_GL.pdf.
27. Goddard GV. Development of epileptic seizures through brain stimulation at low intensity. Nature. 1967;214(5092).
28. Group E. EQ-5D-5L 2009 [Available from: https://euroqol.org/eq-5d-instruments/eq-5d-5l-about/.
29. Julious SA. Sample size of 12 per group rule of thumb for a pilot study. Pharmaceutical Statistics. 2020;4(4):287-91.
30. Sim J, Lewis M. The size of a pilot study for a clinical trial should be calculated in relation to considerations of precision and efficiency. J Clin Epidemiol. 2012;65(3):301-8.
31. Common Terminology Criteria for Adverse Events (CTCAE) - CTCAE_4.03_2010-06-14_QuickReference_5x7.pdf 2020 [Available from: https://www.ncbi.nlm.nih.gov/pubmed/.
32. Dindo D, Demartines N, Clavien PA. Classification of surgical complications: a new proposal with evaluation in a cohort of 6336 patients and results of a survey. Ann Surg. 2004;240(2):205-13.
33. NVIVO. Qualitative Data Analysis Software | NVivo 2020 [Available from: https://www.qsrinternational.com/nvivo-qualitative-data-analysis-software/home.
34. Rai ZL, Feakins R, Pallett LJ, Manas D, BR D. Irreversible Electroporation (IRE) in Locally Advanced Pancreatic Cancer: A Review of Current Clinical Outcomes, Mechanism of Action and Opportunities for Synergistic Therapy. Journal of clinical medicine. 2021;10(8).
35. Fogel DB. Factors associated with clinical trials that fail and opportunities for improving the likelihood of success: A review. Contemp Clin Trials Commun. 2018;11:156-64.
36. Morgan B, Hejdenberg J, Hinrichs-Krapels S, Armstrong D. Do feasibility studies contribute to, or avoid, waste in research? PLoS One. 2018;13(4):e0195951.
37. Pisters PW, Abbruzzese JL, Janjan NA, Cleary KR, Charnsangavej C, Goswitz MS, et al. Rapid-fractionation preoperative chemoradiation, pancreaticoduodenectomy, and intraoperative radiation therapy for resectable pancreatic adenocarcinoma. J Clin Oncol. 1998;16(12):3843-50.
38. Yeung RS, Weese JL, Hoffman JP, Solin LJ, Paul AR, Engstrom PF, et al. Neoadjuvant chemoradiation in pancreatic and duodenal carcinoma. A Phase II Study. Cancer. 1993;72(7):2124-33.
39. Evans DB, Rich TA, Byrd DR, Cleary KR, Connelly JH, Levin B, et al. Preoperative chemoradiation and pancreaticoduodenectomy for adenocarcinoma of the pancreas. Arch Surg. 1992;127(11):1335-9.

For peer review only

40. He C, Wang J, Sun S, Zhang Y, Li S. Immunomodulatory Effect after Irreversible Electroporation in Patients with Locally Advanced Pancreatic Cancer. J Oncol. 2019;2019:9346017.
41. Pandit H, Hong YK, Li Y, Rostas J, Pulliam Z, Li SP, et al. Evaluating the Regulatory Immunomodulation Effect of Irreversible Electroporation (IRE) in Pancreatic Adenocarcinoma. Ann Surg Oncol. 2019;26(3):800-6.

For peer review only

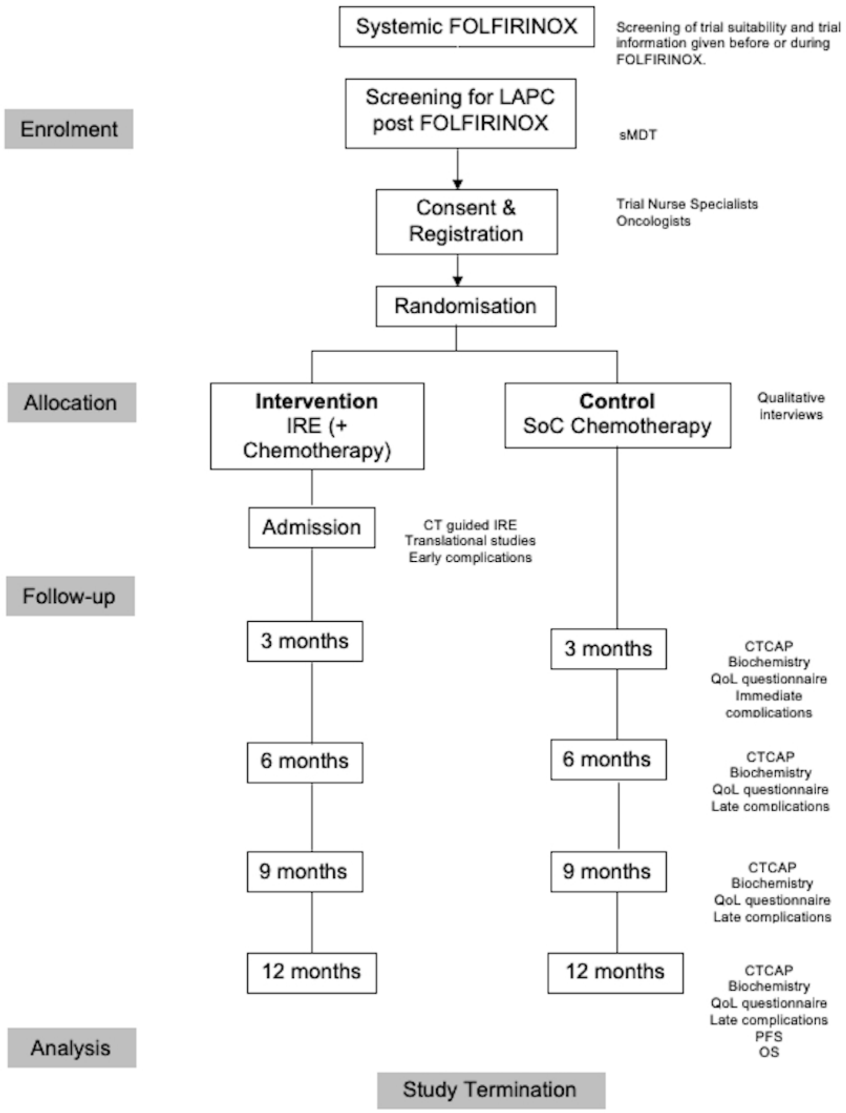

Figure 1. Trial Flow Diagram.

Inclusion criteria

- Tissue confirmation of adenocarcinoma
- Successful completion of FOLFIRINOX
- Cancer deemed unresectable at sMDT review
- Ability to provide informed consent
- ≥ 18 years of age
- Considered amenable to IRE by pancreas IR
- WHO Performance status 0 /1.
- Maximum cancer diameter of 3.5 cm at time of IRE treatment.
- Medically fit for general anaesthetic

Exclusion Criteria

- First line chemotherapy other than FOLFIRINOX
- Concomitant malignancy (except skin and prostate cancers)
- Metastatic/Borderline resectable disease
- Untreated gastric outlet/biliary obstruction
- Co-morbidity precluding general anaesthesia +/- neuromuscular blockade
- Indwelling electrical devices
- CKD≥3
- History of neurological disease
- Abdominal varices
- Active bleeding
- Anticoagulation which cannot be discontinued,
- Coagulopathy (INR>1.5)/**any one** of the following:
  - Hb< 8 g/dL
  - absolute neutrophil count< 1500 cells/mL
  - platelet count< 50,000

For peer review only

Treatment of unresectable Locally Advanced Pancreatic cancer with Percutaneous Irreversible Electroporation (IRE) following initial systemic chemotherapy (LAP-PIE) trial: Study protocol for a feasibility randomised controlled trial.

Protocol

Version II- August 2021

Study Principal Investigator:	Brian R. Davidson, MB ChB, MD, FRCS (Eng.), FRCPS (Glasg), Division of Surgery and Interventional Science, University College London
Sponsors:	Royal Free London NHS Foundation Trust
Protocol contributors:	Zainab Rai, (Division of Surgery and Interventional Science, University College London)<br>Veronica Ranieri, (Division of Surgery and Interventional Science, University College London)<br>Michael Stackpoole, (Liverpool Clinical Trials Unit, Liverpool)<br>Peter Littler, (Interventional Radiology, Freeman Hospital, Newcastle)<br>Dan Palmer, (Liverpool Clinical Trials Unit, Liverpool)<br>Paula Ghaneh, (Liverpool Clinical Trials Unit, Liverpool)<br>Kurinchi Gurusamy, (Division of Surgery and Interventional Science, University College London)<br>Derek Manas, (Newcastle NHS Foundation Trust, Newcastle)<br>Elena Pizzo, (Statistical sciences, University College London)<br>Eftychia E Psarelli, (University of Liverpool)<br>Roopinder Gilmore(Royal Free London NHS Foundation Trust)<br>Praveen Peddu (King's College Hospital NHS Trust),

For peer review only - http://bmjopen.bmj.com/site/about/guidelines.xhtml

David C Bartlett, (University Hospitals Birmingham NHS Foundation Trust Birmingham)
Nico de'Liguori Carino (Manchester Royal Infirmary)
Brian Davidson, (Centre for Surgical Innovation, Organ Regeneration and Transplant University College London)

Trial identifiers: Trial registered with International Standard Randomised Controlled Trial Number at https://www.isrctn.com/ISRCTN14986389
Trial ID: ISRCTN14986389
Date registered: 09/02/2021
PB-PG-1217-20042

For peer review only

For peer review only - http://bmjopen.bmj.com/site/about/guidelines.xhtml

REVISION HISTORY

Version I	Date 29/10/2020	Amendment Text	Description
Version II	Date 14/08/2021		As per Reviewer's comments

For peer review only

For peer review only - http://bmjopen.bmj.com/site/about/guidelines.xhtml

Table of Contents

1. TRIAL SUMMARY ....
2. INTRODUCTION ....
2.1. Background and rationale ....
2.2. Objectives ....
2.3. Trial design ....
3. METHODS ....
3.1. Study setting ....
3.2. Eligibility criteria ....
3.2.1. Inclusion criteria ....
3.2.2. Exclusion criteria ....
3.3. Interventions ....
3.3.1. Intervention description ....
3.3.2. Modifications ....
3.3.3. Adherence ....
3.3.4. Concomitant care ....
3.4. Outcomes ....
3.5. Participant timeline ....
3.6. Sample size ....
3.7. Recruitment ....
3.8. Allocation ....
3.9. Blinding (masking) ....
3.9.1. Blinding mechanism ....
3.9.2. Emergency unblinding ....
3.10. Data collection ....
3.10.1. Trial procedures and evaluations ....
3.10.2. Retention ....
3.11. Data management ....
3.12. Statistical methods ....
3.12.1. Outcomes ....
3.12.2. Additional analyses ....
3.12.3. Analysis population and missing data ....
3.13. Data monitoring ....
3.13.1. Formal committee ....
3.13.2. Interim analysis ....
3.14. Safety/harms ....
3.15. Auditing ....
4. ETHICS AND DISSEMINATION ....
4.1. Research ethics approval ....
4.2. Protocol amendments ....
4.3. Informed consent process ....
4.4. Confidentiality ....
4.5. Declaration of interests ....
4.6. Access to data ....
4.7. Dissemination policy ....

For peer review only - http://bmjopen.bmj.com/site/about/guidelines.xhtml

4.7.1. Trial results ....
4.7.2. Authorship ....
4.7.3. Reproducible research ....
5. STUDY ADMINISTRATION ....
5.1. Key contacts ....
5.2. Funders ....
5.3. Roles and responsibilities ....
5.3.1. Protocol contributors ....
5.3.2. Sponsor and funder ....
5.3.3. Trial committees ....
6. REFERENCES ....
7. APPENDICES ....
7.1. Informed consent materials ....

For peer review only

For peer review only - http://bmjopen.bmj.com/site/about/guidelines.xhtml

1. TRIAL SUMMARY

World Health Organization Registration Data Set

Title	Treatment of unresectable Locally Advanced Pancreatic cancer with Percutaneous Irreversible Electroporation (IRE) following initial systemic chemotherapy (LAP-PIE) trial: Study protocol for a feasibility randomised controlled trial.
Primary registry and trial identifying number	Trial registered with International Standard Randomised Controlled Trial Number at https://www.isrctn.com/ISRCTN14986389<br>Trial ID: ISRCTN14986389<br>Date registered: 09/02/2021
Secondary identifying numbers	PB-PG-1217-20042
Sources of monetary or material support	Sources and types of financial, material, and other support<br>**Funding body:**<br>National Institute for Health Research (NIHR), Research for Patient Benefit (RfPB) programme<br>Central Commissioning Facility<br>Grange House<br>15 Church Street<br>Twickenham<br>TW1 3NL<br>**Training support:**<br>Angiodynamics. Manufacturer of CE marked device that is in use in clinics already. Angiodynamics will provide training and support as normal during this study and supply of the needle probes used through the duration.<br>**Other Support.**<br>Operational Director Liverpool Clinical Trials Centre Block C, Waterhouse Building1-3 Brownlow StreetLiverpoolL69 3GLTel: 0151 794 8897Fax: 0151 794 8931LAP-PIE@liverpool.ac.uk
Primary sponsor	Royal Free London NHS Foundation Trust
Secondary sponsors (if any)	Not applicable
Central contact	Operational Director<br>Liverpool Clinical Trials Centre Block C, Waterhouse Building 1-3 Brownlow Street Liverpool L69 3GL<br>0151 794 8897

For peer review only - http://bmjopen.bmj.com/site/about/guidelines.xhtml

	LAP-PIE@liverpool.ac.uk
Study officials/Investigators	Brian R. Davidson, MB ChB, MD, FRCS (Eng), FRCPS (Glasg)<br>Division of Surgery and Interventional Science, University College London<br>Royal Free Hospital, 9th Floor (East), 2QG, 10 Pond St, London NW3 2PS<br>020 7794 0500, Ext.33603<br>b.davidson@ucl.ac.uk<br>Study Principal Investigator
Brief title	Treatment of unresectable Locally Advanced Pancreatic cancer with Percutaneous Irreversible Electroporation (IRE) following initial systemic chemotherapy: a feasibility randomised controlled trial
Acronym	LAP-PIE
Countries of recruitment	United Kingdom
Condition(s) or focus of study	Locally advanced pancreatic cancer, Irreversible electroporation, Feasibility trial
Interventions	
IRE (interventional)	Intervention type: Device<br>Intervention name: Irreversible Electroporation<br>Other names: IRE<br>Intervention description: The length of the electrical pulse and amperage will be decided by the treating interventional radiologist and will be between 70-100 microseconds with an amperage of between 20-40 AMPs. The treating interventional radiologist will have relevant expertise in IRE procedures with a minimum of 5 IRE procedures on liver or pancreas performed previously.
Standard of Care (control)	Intervention type: Drug<br>Intervention name: 5-FluoroUracil, Leucovorin, Irinotecan and Oxaliplatin<br>Other names: FOLFIRINOX<br>Intervention description: Patients randomised to receive further chemotherapy will commence treatment within 6 weeks after sMDT or 4 weeks after randomisation, whichever is sooner. The second line chemotherapy

For peer review only - http://bmjopen.bmj.com/site/about/guidelines.xhtml

	regime will be selected at the discretion of the treating oncologist and will adhere to NICE guidelines and local protocols.
Key eligibility criteria	Age eligibility: 18 years or older<br>Sex eligibility: Both<br>Accepts healthy volunteers: No<br><br>Inclusion criteria:<br>1. Tissue confirmation<br>2. Successful completion of FOLFIRINOX<br>3. Cancer deemed inoperable at sMDT review,<br>4. WHO Performance status 0 /1<br>5. Maximum cancer diameter of 3.5 cm at time of IRE treatment<br><br>Exclusion criteria:<br>1. First line chemotherapy other than FOLFIRINOX<br>2. Concomitant malignancy (except skin and prostate cancers)<br>3. Metastatic/Borderline resectable disease<br>4. Untreated gastric outlet/biliary obstruction<br>5. Co-morbidity precluding general anaesthesia +/- neuromuscular blockade<br>6. Indwelling electrical devices<br>7. Chronic Kidney Disease > 3<br>8. History of neurological disease<br>9. Abdominal varices<br>10. Active bleeding<br>11. Anticoagulation which cannot be discontinued<br>12. Coagulopathy
Study design	Study type: Interventional trial<br>Allocation: Randomized<br>Intervention model: Parallel group<br>Primary purpose: Other<br>Phase: Feasibility
Masking	None
Date of enrolment	
Target sample size	50
Recruitment status	Active, not recruiting

For peer review only - http://bmjopen.bmj.com/site/about/guidelines.xhtml

Primary outcomes	To ascertain the feasibility of identifying and recruiting LAPC patients at the selected sites to the LAP-PIE trial. The primary outcome consists of recruitment rate, number of screening failures, number of patients completing study pathway as per protocol and trial withdrawal rate. Timeframe: 36 months
Secondary outcomes	To assess the practicality and technical success of pancreas IRE treatment. Completion rates of IRE procedures, safety and complications of IRE treatment, technical success of intervention, acceptability of pancreas IRE therapy to patients and their clinicians, rate of withdrawal from the study and outcome measures including overall survival (measured from diagnosis and randomisation), progression free survival (measured as a binary outcome) and quality of life will be evaluated. Timeframe: 36 months

For peer review only

For peer review only - http://bmjopen.bmj.com/site/about/guidelines.xhtml

2. INTRODUCTION

2.1. Background and rationale

Surgery (pancreatic resection) is the only treatment with the potential to cure pancreatic cancer. However only 10 to 20% of people are eligible for surgery. One of the major reasons is that the cancer has spread into the surrounding structures, often the superior mesenteric vein and/or artery. This circumstance affecting about 30% of all patients is termed locally advanced pancreatic cancer (LAPC). Currently, the recommended treatment for LAPC is chemotherapy. Those with a response to chemotherapy would again be considered for resection. Overall 65% of patients post chemotherapy remain with localised but unresectable disease (UK SCALOP trial induction protocol data).

These individuals will be invited to participate in this study.

Irreversible electroporation (IRE) is a new method of treating localised cancers which are not suitable for surgery. The IRE treatment destroys cancer cells using electricity. Under general anaesthetic the IRE electrodes are inserted into the borders of the pancreas cancer prior to an electrical charge being passed through the cancer. Early studies of IRE in pancreas cancer show that IRE treatment may increase how long people with LAPC live but these studies were not controlled or randomised. The improved outcome could therefore be related to patient selection. In addition, major complications may result from this treatment (eg acute pancreas inflammation, acute pancreatitis), particularly when the treatment is carried out at open surgery rather than percutaneously.
There is therefore uncertainty on the clinical application of pancreas IRE therapy.

2.2. Objectives

Primary Objective:
To ascertain the feasibility of performing a Randomised Controlled Study on Irreversible Electroporation following initial systemic chemotherapy for Locally Advanced Pancreatic Cancer.

Secondary Objectives:
To Assess:
1. Recruitment to a trial of this nature
2. The practicality and technical success of pancreas IRE treatment
3. The acceptability of pancreas IRE therapy to patients and their clinicians
4. Rate of withdrawal from the study
5. Safety of the IRE treatment
6. Relative importance of different outcome measures (Survival and Quality of Life)

2.3. Trial design

For peer review only - http://bmjopen.bmj.com/site/about/guidelines.xhtml

Trial Design
The LAP-PIE trial is a UK multi-centre, feasibility study that will recruit 50 patients with LAPC in whom the disease has remained localised and unresectable following FOLFIRINOX chemotherapy. All participants will be offered further chemotherapy according to standard centre protocols and half will also be offered IRE. It is an open-label study.

3. METHODS

3.1. Study setting

We are proposing to run a small-scale study (a "feasibility study") that will recruit 50 people with LAPC whose disease has remained localised to the pancreas but is unresectable following initial chemotherapy. All will be offered further chemotherapy according to standard centre protocols and half will also be offered IRE. We will assess whether patients are willing to participate in this study and complete the designated treatment. We will assess the completeness of pancreas IRE therapy (technical success) and the complications of the procedure. We will follow the study participants for 12 months assessing their quality of life and cancer response from imaging.

Nested within the feasibility trial there are qualitative and translational components. The qualitative study will assess patients' and clinicians' perspectives surrounding involvement and participation in a trial of this nature. The translational component aims to determine whether there is a biological significance between post-IRE imaging and peripheral immune response and treatment efficacy.

Each centre will complete a feasibility questionnaire to ensure that the centre has the appropriate facilities, qualified personnel and capacity to set up and run the trial in accordance with the current approved protocol and GCP standards.

A list of study sites can be obtained from contacting the central enquiry for the LAP-PIE trial at LAP-PIE@liverpool.ac.uk

3.2. Eligibility criteria

3.2.1. Inclusion criteria

1. Tissue confirmation
   Histopathological confirmation of pancreatic adenocarcinoma
2. Successful completion of FOLFIRINOX
3. Cancer deemed inoperable at sMDT review,
   sMDT (specialist multi-disciplinary team)
4. WHO Performance status 0 /1
   World Health Organisation (WHO)

For peer review only - http://bmjopen.bmj.com/site/about/guidelines.xhtml

5. Maximum cancer diameter of 3.5 cm at time of IRE treatment
   Retrospective and prospective studies suggest minimal clinical benefit of treating tumours > 3.5cm

3.2.2. Exclusion criteria

1. • First line chemotherapy other than FOLFIRINOX
2. • Concomitant malignancy (except skin and prostate cancers)
3. • Metastatic/Borderline resectable disease
4. • Untreated gastric outlet/biliary obstruction
5. • Co-morbidity precluding general anaesthesia +/- neuromuscular blockade
6. Indwelling electrical devices
7. Chronic Kidney Disease > 3
8. History of neurological disease
9. Abdominal varices
10. Active bleeding
11. Anticoagulation which cannot be discontinued
12. Coagulopathy
    (INR>1.5)/any one of the following, Hb< 8 g/dL, absolute neutrophil count< 1500 cells/mL, platelet count< 50,000.

3.3. Interventions

3.3.1. Intervention description

<u>Intervention</u>

Irreversible electroporation (IRE)

This new form of cancer treatment involves application of electric pulses to increase the permeability of cancer cell membranes. IRE induces cancer cell apoptosis which peaks at about 24 hours after IRE is performed. IRE has been used for ablation of several solid cancers including liver, pancreatic, and prostate cancers on an experimental basis. In pancreatic cancer, IRE has been used in patients for ablation of locally advanced pancreatic cancer either by open surgery, laparoscopically or percutaneously under CT (computed tomography) guidance or ultrasound guidance. In this study all IRE will be delivered percutaneously with CT guidance. The radiologist delivering IRE treatment in this study will be required to have performed a minimum of 5 previous liver or pancreas IRE ablations prior to delivering treatment to patients enrolled in the trial. Post-IRE treatment, patient will be admitted for a minimum 24 hours for pain relief and monitoring and management of immediate procedure-related complications.

Controls (Second line systemic chemotherapy)

Patients randomised to receive further chemotherapy will commence treatment within 6 weeks after sMDT or 4 weeks after randomisation, whichever is sooner. The second line chemotherapy regime will be selected at the discretion of the treating oncologist and will adhere to NICE guidelines and local protocols.

For peer review only - http://bmjopen.bmj.com/site/about/guidelines.xhtml

3.3.2. Modifications

Premature Discontinuation of treatment

Participants may discontinue IRE or standard of care chemotherapy for reasons including, but not limited to:

- Participant-led i.e. request by the participant / person with parental responsibility / legal representative / consultee
- Unacceptable toxicity/side effects
- Intercurrent illness preventing further treatment.
- Pregnancy
- Death
- Clinician-led:
    - Any change in the participant's condition that justifies the discontinuation of treatment in the clinician's opinion.
    - Reasons of non-adherence or non-compliance with treatment or other trial procedures
    - Participant meets an exclusion criterion (either newly developed or not previously recognised)

3.3.3. Adherence

All sites will have a nominated clinical lead in each respective speciality (Surgery, Interventional Radiology and Oncology) who will be responsible for adhering to intervention protocol. They will be invited to attend Investigator meetings and be kept abreast of any changes to the protocol/progress of the trial.

Both the interventional arm (IRE) and the Standard of Care (Chemotherapy) requires patients to attend hospital and therefore no specific adherence strategies have been developed. Participants will be encouraged to attend these appointments.

3.3.4. Concomitant care

Concomitant Medications/Treatments

Concomitant treatment for conditions other than Locally Advanced Pancreatic Cancer may be continued/started throughout the trial in their standard dose as long as it does not affect inclusion or exclusion criteria. Use of concomitant treatment must be recorded in the patient medical record and study CRFs: the name of the drug, dose, start/stop dates and the indication for it are necessary.

Use of any other non-marketed or investigational products during the trial are NOT permitted.

Supportive medications such as analgesics, anti-emetics etc. should be given per local practice. Analgesic use prior to commencing second line therapy should be documented in the Case Report Form (CRF) with drugs, dose and frequency of use.

For peer review only - http://bmjopen.bmj.com/site/about/guidelines.xhtml

For peer review only

Medications Permitted

All medication necessary for the wellbeing of the patient, which is not expected to interfere with the evaluation of the IRE therapy and aftercare may be administered. The local investigator may contact the study team for guidance from the Chief Investigator or delegated medical expert. Particular attention should be given to treatment related to the side effects and risks of the IRE therapy.

IRE will be carried out under general anaesthetic and neuromuscular relaxation. Broad spectrum antibiotics will be administered pre and for 2 doses post procedure.

Medications Not Permitted/ Precautions Required

There are no medications which would contra-indicate pancreas IRE therapy.
As with all interventions patients with abnormal blood clotting or taking anticoagulant medication should be considered with caution and withdrawal of anticoagulant medication or alternative short-lived anticoagulant administered until the patient recovers from the procedure at which time routine medication will be re-commenced.

Data on Concomitant Medication

The systemic chemotherapy (drug/dose/duration) will be recorded for both treatment groups over the 12 month follow up period. Concomitant medication will be collected at baseline and updated through the study duration. Each visit CRF will prompt the local research team to complete the concomitant medication form for any new drugs and to ensure the start and stop date of previous medication is stated.

3.4. Outcomes

Outcomes

The primary objective of the LAP-PIE trial is to ascertain feasibility of such a trial concept. In particular, the ability to identify and recruit LAPC patients at the selected sites to the LAP-PIE trial will be evaluated. The primary outcome consists of recruitment rate, number of screening failures, number of patients completing study pathway as per protocol and trial withdrawal rate.

The secondary objectives of the LAP-PIE trial aim to assess the practicality and technical success of pancreas IRE treatment. Completion rates of IRE procedures, safety and complications of IRE treatment, technical success of intervention, acceptability of pancreas IRE therapy to patients and their clinicians, rate of withdrawal from the study and outcome measures including overall survival (measured from diagnosis and randomisation), progression free survival (measured as a binary outcome) and quality of life will be evaluated.

3.5. Participant timeline

For peer review only - http://bmjopen.bmj.com/site/about/guidelines.xhtml

Schedule of enrolment, interventions, and assessments of the LAP-PIE trial.

	**Study Period**									
	**Enrolment**	**Allocation**	**Post-Allocation**							**Close-out**
**TIMEPOINT**	$-t_1$	**0**	***Pre-IRE***	***6 hours post -IRE***	***6 days post -IRE***	***3 months***	***6 months***	***9 months***	***12 months***	
**ENROLMENT:**										
**Eligibility screen**	X									
**PIS given**	X									
Systemic treatment with FOLFIRINOX	X									
sMDT	X									
Informed consent	X									
**Allocation**		X								
**INTERVENTIONS:**										
IRE (intervention)			◆—	—	—	—	—	—	—◆	
Systemic chemotherapy (SoC)						◆—	—	—	—◆	
**ASSESSMENTS:**										
CT CAP	X					X	X	X	X	
Blood tests	X		X	X	X	X	X	X	X	X
HRQoL						X	X	X	X	X
Complications						X	X	X	X	X

PIS; Patient Information Sheet, FOLFIRINOX; 5-FluoroUracil, Leucovorin, Irinotecan and Oxaliplatin, sMDT; specialist Multi-Disciplinary Team, HRQoL; Health Related Quality of Life

For peer review only

For peer review only - http://bmjopen.bmj.com/site/about/guidelines.xhtml

3.6. Sample size

A convenient sample size of 50 patients, randomised equally to the two groups, has been chosen for this feasibility study. Formal sample size calculations are not appropriate for a feasibility study. However, this size will allow us to assess whether the consent rate of 20% has been achieved with a 95% confidence interval of +/- 11%. This sample size will also allow us to assess whether the technical success of IRE is 50% with a 95% confidence interval of +/- 14%, and loss to follow-up is 10% with a 95% confidence interval of +/-8%. In addition, sample sizes between 24 and 50 have been recommended to estimate the standard deviation required for a sample size calculation to allow us to design a large randomised controlled trial aimed at evaluating the cost effectiveness of IRE following chemotherapy for locally advanced pancreatic cancer (24, 25).

The recruitment target is 50 patients in 15 months from 7 UK centres, assuming a recruitment rate of 0.8 patients per site per month

3.7. Recruitment

This is a feasibility trial and one key aspect of feasibility evaluation is the ability to recruit patients with unresectable locally advanced pancreatic cancer into a trial of this nature. We will explore the reasons for participation and non-participation of patients and patients' and clinicians' acceptability of the trial to assist in optimisation of recruitment strategies employed for the subsequent trial. Non-participation can be related to how the clinical trial is presented to the patient, and how the patient assimilates this information. It is therefore important to understand how patients perceive information about potential participation and their experiences of receiving information on the trial.

3.8. Allocation

Allocation Sequence Generation

Participants will be randomised on a 1:1 basis. Randomisation lists shall be produced by the trial statistician using randomly permuted blocks Lists created using Stata (Version 14 or higher) using the add-on package 'ralloc'

Patient allocations will be irrevocably generated upon completion of the web-based randomisation form by a delegated member of the trial research team. Allocation concealment will be ensured as the service will not release the randomisation code until the patient has been recruited into the trial; this takes place after all baseline measurements have been completed.

For peer review only - http://bmjopen.bmj.com/site/about/guidelines.xhtml

3.9. Blinding (masking)

3.9.1. Blinding mechanism

The intervention is a procedure requiring expertise and general anaesthesia therefore it is not possible or appropriate to design this study as a blinded trial

3.9.2. Emergency unblinding

Not a blinded study therefore not applicable

3.10. Data collection

3.10.1. Trial procedures and evaluations

Data Collection Methods

Each participating site will maintain appropriate medical and research records for this trial, in compliance with ICH E6 GCP, Section 4.9 and regulatory and institutional requirements for the protection of confidentiality of subjects.

Data that is to be recorded directly on the electronic Clinical Record Forms (eCRF) or workbooks (i.e. no prior electronic or written record of the data), is to be considered to be source data e.g. questionnaires

Date(s) of informed consent processes (including date of provision of patient information, randomisation number and the fact that the patient is participating in a clinical trial (including possible treatment arms) will be added to the patient's medical record chronologically.

Data will be entered into MACRO and PHAROS by members of the research team at site. Training will be provided prior to any data entry. The eCRF workbooks that replicate each MACRO and PHAROS form must be retained at site for the study.

Questionnaires are a source document and sites will retain a copy at site in the Investigator Site File (ISF) (part of the Trial Master File).

Quality Assurance and Control

Quality assurance will be maintained to the required level via electronic database validations, internal LCTC quality processes and procedures, training of those involved in trial procedures and regular monitoring and audit.

The TMG will be involved in monitoring screening, randomisation and consent rates, as well as compliance with processes detailed in the protocol. Any activity that is determined to be a breach of the study protocol will be recorded on a protocol deviation form and reviewed by the study CI to confirm severity, the impact on the study and whether any corrective and preventative action is required.

The LAP-PIE study will have a designated data management and query resolution plan in place and specific instructions on how to complete the eCRF forms and workbooks. A

For peer review only - http://bmjopen.bmj.com/site/about/guidelines.xhtml

member of the study team will periodically review the study for missing or erroneous/anomalous data based on validations built into the database.

Study Instruments

The EQ5D-5L health questionnaire will be used at Baseline and each follow up visit in order to assess quality of life for all patients on the study. The EQ5D-5L is descriptive system for health-related quality of life states in adults, consisting of five dimensions (Mobility, Self-care, Usual activities, Pain & discomfort, Anxiety & depression), each of which has five severity levels that are described by statements appropriate to that dimension. It has been used to assess quality of life in a number of conditions (ref) and is considered a reliable and valid health related questionnaire (ref).

Data collection forms can be requested by contacting the central LAP-PIE trial team on LAP-PIE@liverpool.ac.uk

3.10.2. Retention

Plans to Promote Participant Retention and Complete Follow-up.

Specific plans to promote participant retention and trial completion are not appropriate for a feasibility trial because part of the primary outcome of feasibility is understanding the reasons for discontinuation. This information will be used to plan a larger randomised control trial where participant retention methods will be developed based on information from this feasibility trial.

Outcome Data to be collected for participants who discontinue/deviate from interventions protocols

Discontinuation from study intervention does not mean discontinuation of the study altogether, and the remaining study procedures, follow up assessment / visits and data collection should be completed as indicated in the protocol (unless consent is specifically withdrawn). Patients finishing the study should have an end of Study eCRF completed, (if due to patient death, there is a notification of death eCRF), detailing the reason for not continuing.

Participant Withdrawal from Follow Up visits

Patients are free to withdraw from follow up at any time without providing a reason, though a reason should be recorded if one is given. Those who wish to withdraw from further follow-up will have the data collected up to the point of that withdrawal included in the analyses. The participant will not contribute further data to the study and the CTU should be informed. The End of Study eCRF should be completed to notify the CTU of this withdrawal.

If patients express a wish to withdraw from follow up, the research team at site should ascertain if this is for all elements of trial follow-up, or if for example data from routine

For peer review only - http://bmjopen.bmj.com/site/about/guidelines.xhtml

assessments can still be collected for the trial. In the case of ongoing adverse events, participants should be given appropriate care under medical supervision until the symptoms of any adverse event resolve or the patient's condition becomes stable. Any SAEs will be notifiable to the CTU via processes detailed in Section 11 even if a participant has withdrawn from follow up.

3.11. Data management

Confidentiality

Patients will consent for the use of their data, including identifiable data such as NHS number and Date of Birth before taking part in the study. Though the consent form will have their name and NHS number, this data will not be used on any eCRF or Workbook. Patients will instead be referred to using their screening number, and then randomisation number once allocated to one of the study arms.

Consent forms with patient identifiable information will be kept within the patient file at the local research site and a copy at the LCTC to evidence that the patient consented to study. At both locations the consent forms will be stored within a locked cabinet/drawer within a secure office and department.

The LCTC as part of The University of Liverpool will preserve the confidentiality of participants taking part in the study. The University of Liverpool is registered as a Data Controller with the Information Commissioners Office.

Breaches of data protection principles or regulations identified by LCTC will be notified promptly to the trial Sponsor and The University of Liverpool's Data Protection Officer and appropriate processes followed

Quality Assurance and Control

Quality assurance will be maintained to the required level via electronic database validations, internal LCTC quality processes and procedures, training of those involved in trial procedures and regular monitoring and audit.

The TMG will be involved in monitoring screening, randomisation and consent rates, as well as compliance with processes detailed in the protocol. Any activity that is determined to be a breach of the study protocol will be recorded on a protocol deviation form and reviewed by the study CI to confirm severity, the impact on the study and whether any corrective and preventative action is required.

The LAP-PIE study will have a designated data management and query resolution plan in place and specific instructions on how to complete the eCRF forms and workbooks. A member of the study team will periodically review the study for missing or erroneous/anomalous data based on validations built into the database.

For peer review only - http://bmjopen.bmj.com/site/about/guidelines.xhtml

The frequency of the LAP-PIE Trial Management Group meeting will be outlined in the monitoring plan, along with what data in the Central Monitoring Report will be reviewed

3.12. Statistical methods

3.12.1. Outcomes

Primary Outcome

1. Recruitment rate will be measured by:

(I) the total number of patient participants randomised per month and

(ii) ratio of successful recruitment to eligible patients approached.

1. Number of screening failures.
2. Number of patients completing study pathway as per protocol will be measures as the number of randomised patients completing the full study pathway with no major protocol deviations.
3. Trial withdrawal rate will be measured using the total number of patients randomised in the study. In addition, withdrawal rates will be presented by trial withdrawal reasons and withdrawal stage for both arms.

Secondary Outcomes

Adherence to protocol

The number of minor and major protocol deviations will be collected on a patient participant and site level.

Mortality rate

Overall survival will be recorded for all patients.

1. Survival is defined as the number of days between date of randomisation and date of death due to any cause or the date of the last follow-up if the patient is still alive at the time of final trial follow up at 12 months post randomisation.
2. Survival is defined as the number of days between date of diagnosis and date of death due to any cause or the date of the last follow-up if the patient is still alive at the time of final trial follow up at 12 months post randomisation.

An overall survival rate will be calculated as also a 9-month using both definitions.

Technical success

Proportion of patients with a successful percutaneous IRE (complete local therapy) as defined in section 10.8 (Assessing Efficacy)

Surgical rate.

It is possible that patients will be considered for surgery having completed second line therapy (IRE and chemo or chemo alone). If so, the following will be recorded:

1. Number of patients who become eligible for surgery over the total number of patients recruited.
2. Number of patients proceeding to surgery over the total number of patients who become eligible for surgery.

For peer review only - http://bmjopen.bmj.com/site/about/guidelines.xhtml

Resection rate (R0/R1)
The ratio will be calculated over numbers undergoing tumour resection in each arm of the trial. Pathology of the specimens will determine the overall R0/R1 resection rate. The R0 resection margin will be recorded according to the Royal College of Pathologists report on Standards and datasets for reporting cancers.

Local or systemic disease progression on follow up
The proportion of patients with local or systemic disease progression as defined by RECIST criteria in each arm of the trial will be calculated at 3,6,9 and 12 months post.

Chemotherapy-related toxicities.
Formal toxicity assessments will be performed and graded according to section 12. The proportion of patients experiencing grade 3/4 toxicity will be calculated.

IRE Procedure related complications
Complications related to IRE therapy will be recorded and the severity grading will be performed according to Clavien-Dindo classification. The proportion of patients experiencing grade III and above toxicity will be calculated.

Quality of life
Quality of life will be assessed with the EQ-5D-5L. The questionnaires will be completed as per the Schedule of Trial Procedures in Section 3 by all patients. The data will be scored according to the EQ-5D-5L scoring manual.

3.12.2. Additional analyses
Not applicable

3.12.3. Analysis population and missing data
Not applicable for a feasibility trial

3.13. Data monitoring
3.13.1. Formal committee
The Data Monitoring Committee (DMC) will consist of an independent chairperson, plus 2 independent members; who are experts in the field of Pancreatic Cancer, and an independent biostatistician.
The DMC will be responsible for reviewing and assessing recruitment, interim monitoring of safety and effectiveness, trial conduct and external data. The DMC will first convene prior to start of recruitment and will then define frequency of subsequent meetings (at least annually).

Further details of its charter can be requested from central enquires on LAP-PIE@liverpool.ac.uk

3.13.2. Interim analysis

For peer review only - http://bmjopen.bmj.com/site/about/guidelines.xhtml

No formal interim analyses will be performed during the course of the feasibility study. The Data and Monitoring Committee (DMC) will assess the actual recruitment rates and make decisions on whether to stop the trial in light of the analyses presented. These analyses will be performed at the LCTC. A decision to discontinue recruitment, in all patients or in selected subgroups will be made only if the result is likely to convince a broad range of clinicians including participants in the trial and the general clinical community. The DMC will make formal recommendations to the Trial Steering Committee (TSC) as to the continuation of the trial.

3.14. Safety/harms

Assessment of Complications

Adverse Events (AE) for the Standard of Care Chemotherapy will be defined using the Common Terminology Criteria for Adverse Events (CTCAE) Version 4.0 (ref). AEs for IRE will be defined using the Clavien Dindo classification of surgical complications (ref). Complications greater than grade 3 on the CTCAE scale and greater than grade III on the Clavien Dindo scale will be reported.

A Serious Adverse Event (SAE) for patients randomised to receive Standard of Care Chemotherapy only, will be compared against the reference safety information (RSI) within the current summary of product characteristics (SmPC) for that drug. The RSI will be taken from the latest SmPC available for the drug on https://www.medicines.org.uk/emc. The RSI for IRE will be from the Nanoknife system users' manual.

All safety events which are recorded for the study will be reported following the procedures detailed below. The occurrence of a safety event may come to the attention of research staff during routine study visits, from the participant's notes, directly from the participant or by other means. Note that reporting procedures vary dependent on the nature of the incident (i.e. "serious" events are to be reported to LCTC in an expedited manner). Any questions concerning adverse event reporting should be directed to the LCTC in the first instance. A flowchart is provided in the additional documents to aid in determining reporting procedures for different types of adverse events.

Reporting Safety Events to the LCTC

SAE reporting will be done from the date of randomisation until the end of study period at 12 months following randomisation

All AEs, whether serious or non-serious, that occur during this timeframe will be recorded via the LCTC's pharmacovigilance database: https://lctu.org.uk/PV/login.aspx

Sites will use the LCTC pharmacovigilance database to report adverse, instruction on how to enter AEs on the pharmacovigilance database will be provided to research staff at site initiation as a work instruction. Paper CRFs will be available for download from the LCTC PORTAL for use only in case the pharmacovigilance database is not working.

For peer review only - http://bmjopen.bmj.com/site/about/guidelines.xhtml

These can be sent in to the LCTC via Fax for upload by the LAP-PIE Study Coordinator or Data Manager.

Follow-up After Adverse Events
All reportable adverse events will be followed until satisfactory resolution or until the investigator responsible for the care of the participant deems the event to be chronic or the patient to be stable.

When reporting "serious" safety events the investigator responsible for the care of the participant will apply the following criteria to provide information relating to event outcomes:

- resolved
- resolved with sequelae (specifying with additional narrative)
- not resolved/ongoing
- ongoing at final follow-up
- fatal or unknown.

3.15. Auditing

Quality assurance will be maintained to the required level via electronic database validations, internal LCTC quality processes and procedures, training of those involved in trial procedures and regular monitoring and audit.

The Trial Monitoring Group (TMG) will be involved in monitoring screening, randomisation and consent rates, as well as compliance with processes detailed in the protocol. Any activity that is determined to be a breach of the study protocol will be recorded on a protocol deviation form and reviewed by the study CI to confirm severity, the impact on the study and whether any corrective and preventative action is required.

Independent oversight of the trial will be provided by the members of the DMC.

The LAP-PIE study will have a designated data management and query resolution plan in place and specific instructions on how to complete the eCRF forms and workbooks. A member of the study team will periodically review the study for missing or erroneous/anomalous data based on validations built into the database.
The frequency of the LAP-PIE TMG meeting will be outlined in the monitoring plan, along with what data in the Central Monitoring Report will be reviewed

4. ETHICS AND DISSEMINATION

4.1. Research ethics approval

Statement of Compliance

The trial involves the use of a CE marked medical device which is utilised for the intended purpose therefore this trial is not within the remit of the Medical Devices Regulation.

For peer review only - http://bmjopen.bmj.com/site/about/guidelines.xhtml

The procedures detailed within this protocol are compliant with the Ionising Radiation (Medical Exposure) Regulations, and appropriate review by a Medical Physics Expert and Clinical Radiation Expert has been undertaken.

Ethical Considerations

The LAP-PIE trial will be conducted in accordance with, but not limited to, the Human Rights Act 1998, the GDPR, Freedom of Information Act 2000 subject to the provisions of sections 41 and 43 thereof, the EU Clinical Trials Directive, the Medicines for Human Use (Clinical Trials) Regulations 2004, the Medicines Act 1968, the Human Tissue Act 2004, ICH GCP, the Declaration of Helsinki 1996 and the NHS Research Governance Framework for Health and Social Care, as amended from time to time.

Where patients agree to take part in the study, they will be informed of how data are recorded, collected, stored and processed and may be transferred to other countries, in accordance with The General Data Protection Regulation (GDPR) (EU) 2016/679.

This study may be terminated at the request of the CI, ISDMC, REC if, during the course of the study, concerns about the safety of the study emerge.
The CI will update the ethics committee of any new information related to the intervention when appropriate and this will also be disseminated to the Principal Investigators at each trial centre.

Patients entering the trial who are randomised to have standard of care (SoC) will continue on systemic chemotherapy. As there have been no previous randomised trials of IRE for pancreas cancer, we do not know whether these patients in the SoC arm will have a better or worse survival and quality of life than those receiving IRE.

The trial will abide by the principles of the World Medical Association Declaration of Helsinki and has been designed to be as pragmatic as possible. The protocol has undergone ethical review by an independent Research Ethics Committee and has received a favourable opinion by Brent REC.

Approvals

The trial protocol will receive approval by a National Research Ethics Committee (NREC), but all participating sites must undergo site specific assessment of capacity and capability. A copy of all site approval documents and a copy of the PIS and ICF on local headed paper should be forwarded to LCTC before patients are entered. The LCTC should receive a confirmation of capacity and capability for each new centre via the site's R&D department

4.3. Informed consent process

Written informed consent is required for all patients participating in the LAP-PIE trial. Discussion of objectives, risks and inconveniences of the trial and the conditions under which it is to be conducted are to be provided to patients by staff with appropriate experience. An appropriate Patient Information Sheet (PIS), describing in detail the trial interventions/products, trial procedures and risks will be approved by an independent

For peer review only - http://bmjopen.bmj.com/site/about/guidelines.xhtml

ethical committee (IEC) and the patient will be asked to read and review the document. Upon reviewing the document, the investigator will explain the research study to the patient and answer any questions that may arise. A contact point where further information about the trial may be obtained will be provided.

In obtaining and documenting consent, the research team will comply with applicable regulatory requirements and will adhere to GCP and to the ethical principles that have their origin in the Declaration of Helsinki.

Informed consent will be re-affirmed throughout the trial and all discussions and consent will be documented appropriately. If a potential participant does not want to provide consent, they do not have to give a reason.

Written informed consent will be sought from patients who will be approached by the study team and invited to consider participation. This is a discussion that will take place within the hospital that the patient is attending for their LAPC treatment and assessments.

The PIS includes a detailed explanation of the study and makes clear that the rights and welfare of the participants will be protected; it will be emphasised that consent may be declined or withdrawn at any time in the future without the quality of care being adversely affected. The research team will facilitate verbal discussions about the research and the consent process, as well as providing answers to any questions that arise.

All the points on the main study section of the consent form are mandatory for the patient to be eligible, they may not agree to only a selection of criteria. There are optional statements on the consent form related to the translational sub studies and the qualitative evaluation. Patients who do not wish to participate in the sub studies or qualitative evaluation are still eligible for the LAP-PIE trial.

After verbal and written information has been provided, the individual seeking consent must ensure that the patient has fully understood all the information and will ask if they are happy to consent to participation in the trial. Where this is the case, written informed consent will be obtained by means of a dated patient signature on the consent form. This will be countersigned and dated by the person who obtained informed consent i.e. the PI or other appropriately qualified member of the research team who has been delegated this responsibility.

The person obtaining consent, i.e. the person signing off on the consent form with the patient, must be the Principle Investigator or delegated other, a medical practitioner capable of explaining the study rationale and the treatment options to the patient.

The original signed document will be retained in the trial site's Investigator Site File (ISF) and copies will be made:

- One copy provided to the patient for their information,

For peer review only - http://bmjopen.bmj.com/site/about/guidelines.xhtml

- One copy transferred to the LCTC
- One copy filed in the participant's medical records.

N.B. Details of the consent process (date, persons involved, version and type of information sheet and consent form used) must also be recorded directly into the participant's medical records.

4.4. Confidentiality

Patients will consent for the use of their data, including identifiable data such as NHS number and Date of Birth before taking part in the study. Though the consent form will have their name and NHS number, this data will not be used on any eCRF or Workbook. Patients will instead be referred to using their screening number, and then randomisation number once allocated to one of the study arms.

Consent forms with patient identifiable information will be kept within the patient file at the local research site and a copy at the LCTC to evidence that the patient consented to study. At both locations the consent forms will be stored within a locked cabinet/drawer within a secure office and department.

The LCTC as part of The University of Liverpool will preserve the confidentiality of participants taking part in the study. The University of Liverpool is registered as a Data Controller with the Information Commissioners Office.

Breaches of data protection principles or regulations identified by LCTC will be notified promptly to the trial Sponsor and The University of Liverpool's Data Protection Officer and appropriate processes followed.

Records Retention

The investigator at each investigational site will make arrangements to store the essential trial documents, including the Investigator Trial File, until the LCTC informs the investigator that the documents are no longer to be retained.

The PI at each investigational site will make arrangements to store the essential trial documents (as defined by ICH GCP guidelines) including the Investigator Site File, the applicable participant medical records for the full length of the trial's retention period and will arrange for confidential destruction at the end of this period as instructed by the Sponsor.

The PI is also responsible for archiving all relevant source documents so that the trial data can be compared against source data after completion of the trial (e.g. in case of inspection from authorities). They must ensure the continued storage of the documents, even if they, for example, leave the clinic/practice or retire before the end of required storage period. Delegation of responsibility for this must be documented in writing.

For peer review only - http://bmjopen.bmj.com/site/about/guidelines.xhtml

4.5. Declaration of interests

None declared

4.6. Access to data

The Trial Management Group (TMG) will have access to the final trial dataset. The TMG consists of the Chief Investigator, other lead investigators (clinical and non-clinical) and members of the Liverpool Clinical Trials Centre.
Individual Clinicians must undertake not to submit any part of their individual data for publication without the prior consent of the Trial Management Group.

4.7. Dissemination policy

4.7.1. Trial results

This will be the first controlled trial of pancreas IRE therapy in locally advanced pancreas cancer and will have implications for pancreas cancer patients in the UK and Internationally. The trial protocol will be published at the outset. Data from the trial will be presented at National and International meetings (AUGIS, IHPBA, NCRI, BASO) and the trial will be published in a high impact journal. Further dissemination of trial results will be to charitable organisations and patient groups including dissemination via social media. This dissemination will be facilitated by trial outcome literature reviewed and disseminated by our PPI members. The study is not powered to show effectiveness and it is likely that the data will form the basis of an efficiency and cost effectiveness application for a larger multi centre trial. Experience in optimising recruitment, site set up and developing local radiology expertise will be crucial for the success of the subsequent trial which will be powered from data collected during the feasibility study.

4.7.2. Authorship

All publications will include a list of participants, and if there are named authors, these will include the trial's Chief Investigator(s), Statistician(s) and Trial Manager(s) involved at least. If there are no named authors (i.e. group authorship) then a writing committee will be identified that will include these people, at least.

4.7.3. Reproducible research

On completion of the research, a Final Trial Report will be prepared and submitted to the REC. The results of LAP-PIE will be published regardless of the results.

There are no plans for granting public access, however data sharing will be supported via access through the Trial Management Group (TMG).

5. STUDY ADMINISTRATION

5.1. Key contacts

Study Principal Investigator

Brian R. Davidson, MB ChB, MD, FRCS (Eng), FRCPS (Glasg)

For peer review only - http://bmjopen.bmj.com/site/about/guidelines.xhtml

Division of Surgery and Interventional Science, University College London
Royal Free Hospital, 9th Floor (East), 2QG, 10 Pond St, London NW3 2PS
020 7794 0500, Ext.33603
b.davidson@ucl.ac.uk

Co-Principal Investigator

Zainab L. Rai, MBBS, BSc, MRCS (Eng)
Division of Surgery and Interventional Science, University College London
Royal Free Hospital, 9th Floor (East), 2QG, 10 Pond St, London NW3 2PS
Zainab.rai@nhs.net

Central contact

Operational Director

Liverpool Clinical Trials Centre Block C, Waterhouse Building 1-3 Brownlow Street
Liverpool L69 3GL
0151 794 8897
LAP-PIE@liverpool.ac.uk

Sponsor

Research & Development Office Lower Ground Floor Finance Office (LG/306)
Royal Free London NHS Foundation Trust
Pond Street London
London, London, NW3 2QG
United Kingdom
020 7317 7558
rf.randd@nhs.net

5.2. Funders

For peer review only

For peer review only - http://bmjopen.bmj.com/site/about/guidelines.xhtml

Sources and types of financial, material, and other support
Funding body: National Institute for Health Research (NIHR), Research for Patient Benefit (RfPB) programme
Central Commissioning Facility
Grange House
15 Church Street
Twickenham
TW1 3NL
Training support: Angiodynamics. Manufacturer of CE marked device that is in use in clinics already. Angiodynamics will provide training and support as normal during this study and supply of the needle probes used through the duration.
Other Support. Operational Director
Liverpool Clinical Trials Centre
Block C, Waterhouse Building
1-3 Brownlow Street
Liverpool
L69 3GL
Tel: 0151 794 8897
Fax: 0151 794 8931
LAP-PIE@liverpool.ac.uk

For peer review only

5.3. Roles and responsibilities

5.3.1. Protocol contributors

Zainab L Rai, Veronica Ranieri, Stackpoole M, Littler P, Palmer D, Paula Ghaneh, Gurusamy K, Manas D, Elena Pizzo, Eftychia E Psarelli, Roopinder Gilmore, Praveen Peddu, David C Bartlett, Nico de'Liguori Carino, Davidson B and the UK LAP-PIE study group

Acknowledgement
This study is funded by the UK NIHR RfPB programme (PB-PG-1217-20042). The views expressed are those of the author(s) and not necessarily those of the NHS, the NIHR or the Department of Health.

5.3.2. Sponsor and funder

Study sponsor and funder had no role in the study design, data collection and management, data analysis and interpretation and were not involved in the decision to submit report for publication. They do not have ultimate authority over any of these activities.

5.3.3. Trial committees

Liverpool Clinical Trial Centre (LCTC) at the University of Liverpool in collaboration with the Chief Investigator, will have overall management responsibility and will be responsible for trial management activities including (but not limited to), budget

For peer review only - http://bmjopen.bmj.com/site/about/guidelines.xhtml

administration, Trial Master File management, safety reporting, data management, randomisation, statistical analysis, participating site coordination and IMP management.

Trial Management Group (TMG)

A Trial Management Group (TMG) will be formed comprising the Chief Investigator, other lead investigators (clinical and non-clinical) and members of the LCTC. A representative from the Sponsor will also be part of the meeting, and any reports or minutes generated will be circulated for their review. The TMG are responsible for monitoring all aspects of the progress and conduct of the trial and will be responsible for the day-to-day running and management of the trial. The TMG will meet fortnightly throughout the study unless more frequent meetings are required.

For peer review only

For peer review only - http://bmjopen.bmj.com/site/about/guidelines.xhtml

6. REFERENCES

(1) Ghaneh P, Costello E, Neoptolemos JP. Biology and management of pancreatic cancer. *Postgrad Med J* 2008;84(995):478-97.

(2) Balaban EP, Mangu PB, Khorana AA, et al. Locally Advanced, Unresectable Pancreatic Cancer: American Society of Clinical Oncology Clinical Practice Guideline. *J Clin Oncol.* 2016;34(22):2654-68.

(3) Ghosn M, Ibrahim T, Assi T, et al. Dilemma of first line regimens in metastatic pancreatic adenocarcinoma. *World J Gastroenterol* 2016;22(46):10124-30.

(4) Rahib L, Fleshman JM, Matrisian LM, et al. Evaluation of Pancreatic Cancer Clinical Trials and Benchmarks for Clinically Meaningful Future Trials: A Systematic Review. *JAMA Oncol* 2016;2(9):1209-16.

(5) Wang C, Liu X, Wang X, et al. Effects of chemoradiotherapy and chemotherapy on survival of patients with locally advanced pancreatic cancer: A meta-analysis of randomized controlled trials. *Medicine (Baltimore)* 2018;97(36):

(6) Excellence NIfHaC. Overview | Guidance | NICE: NICE; [Available from: Pancreatic cancer in adults: diagnosis and management |. 2020. [Cited: 10/2020]. Available from: https://www.nice.org.uk/guidance/ng85..

(7) V P, P F, P R, et al. Combination of Irinotecan (CPT11) and 5-fluorouracil With an Analysis of Cellular Determinants of Drug Activity. *Biochemical pharmacology.* 1998;56(10):

(8) M D, E M, M O-K, et al. Randomized Phase II Study Evaluating Oxaliplatin Alone, Oxaliplatin Combined with Infusional 5-FU, and Infusional 5-FU Alone in Advanced Pancreatic Carcinoma Patients. *Annals of oncology: official journal of the European Society for Medical Oncology.* 2004;15(3):

(9) Suker M, Beumer BR, Sadot E, et al. A patient-level meta-analysis of FOLFIRINOX for locally advanced pancreatic cancer. *Lancet Oncol.* 2016;17(6):801-10.

(10) Mansson C, Brahmstaedt R, Nygren P, et al. Percutaneous Irreversible Electroporation as First-line Treatment of Locally Advanced Pancreatic Cancer. *Anticancer Res* 2019;39(5):2509-12.

(11) Belfiore MP, Ronza FM, Romano F, et al. Percutaneous CT-guided irreversible electroporation followed by chemotherapy as a novel neoadjuvant protocol in locally advanced pancreatic cancer: Our preliminary experience. *Int J Surg* 2015;21(Suppl 1):34-9.

(12) Lafranceschina S, Brunetti O, Delvecchio A, et al. Systematic Review of Irreversible Electroporation Role in Management of Locally Advanced Pancreatic Cancer. *Cancers (Basel)* 2019;11(11):7181.

(13) Hajatdoost L, Sedaghat K, Walker EJ, et al. Chemotherapy in Pancreatic Cancer: A Systematic Review. *Medicina (Kaunas)* 2018;54(3):48.

(14) MP C, KJ C, EK F, et al. Pre-treatment Assessment of Resectable and Borderline Resectable Pancreatic Cancer: Expert Consensus Statement. *Annals of surgical oncology* 2009;16(7):

(15) Network NCC. Pancreatic Adenocarcinoma, Version 2.2017, NCCN Clinical Practice Guidelines in Oncology - NCCN-2017-pancreatic-cancer.pdf: NCCN; 2017 [Available from: http://clubedopancreas.pt/wp-content/uploads/2018/06/NCCN-2017-pancreatic-cancer.pdf.

For peer review only - http://bmjopen.bmj.com/site/about/guidelines.xhtml

(16) Excellence NIfHaC. Recommendations. Pancreatic cancer in adults: diagnosis and management | Guidance | NICE: NICE. 2020. [Cited: 10/2020]. Available from: https://www.nice.org.uk/guidance/ng85/chapter/Recommendations#managing-resectable-and-borderline-resectable-pancreatic-cancer..
(17) Wills-Percy. St Luke's Cancer Alliance: Cancer treatment 2018. 2018. [Cited: 10/2020]. Available from: http://stlukescanceralliance.co.uk/wp-content/uploads/2018/08/Gem-Cap-V3-8.18.pdf.
(18) Hurt CN, Falk S, Crosby T, et al. Long-term results and recurrence patterns from SCALOP: a phase II randomised trial of gemcitabine- or capecitabine-based chemoradiation for locally advanced pancreatic cancer. *Br J Cancer* 2017;116(10):1264-70.
(19) He C, Wang J, Sun S, et al. Irreversible electroporation versus radiotherapy after induction chemotherapy on survival in patients with locally advanced pancreatic cancer: a propensity score analysis. *BMC Cancer.* 2019;19(394):
(20) Narayanan G, Hosein PJ, Beulaygue IC, et al. Percutaneous Image-Guided Irreversible Electroporation for the Treatment of Unresectable, Locally Advanced Pancreatic Adenocarcinoma. *J Vasc Interv Radiol.* 2017;28(3):342-8.
(21) Kluger MD, Epelboym I, Schrope BA, et al. Single-Institution Experience with Irreversible Electroporation for T4 Pancreatic Cancer: First 50 Patients. *Ann Surg Oncol.* 2016;23(5):1736-43.
(22) Liu S, Qin Z, Xu J, et al. Irreversible electroporation combined with chemotherapy for unresectable pancreatic carcinoma: a prospective cohort study. *Onco Targets Ther* 2019;12(5):1341-50.
(23) W F, JW R, RCG M. Quality of Life Assessment for Patients Undergoing Irreversible Electroporation (IRE) for Treatment of Locally Advanced Pancreatic Cancer (LAPC). *American journal of surgery.* 2019;218(3):
(24) Julious SA.. Sample size of 12 per group rule of thumb for a pilot study.. *Pharmaceutical Statistics* 2005;4(4):287-91.
(25) Sim J, Lewis M.. The size of a pilot study for a clinical trial should be calculated in relation to considerations of precision and efficiency.. *J Clin Epidemiology.* 2012;65(3):301-8.

For peer review only

For peer review only - http://bmjopen.bmj.com/site/about/guidelines.xhtml

7. APPENDICES
7.1. Informed consent materials

PATIENT CONSENT FORM (please read carefully)	
**LAP-PIE:** Treatment of unresectable **L**ocally **A**dvanced **P**ancreatic cancer with **P**ercutaneous **I**rreversible **E**lectroporation (IRE) following initial systemic chemotherapy: a feasibility randomised controlled trial<br>**Study ID:** ________________<br>**NHS Number** ____________________	
**Name of Researcher: ______________________________**	
**Please initial each box**	
**1. I confirm that I have read and understand the patient information sheet date: ………………….. (Version: …………) describing the above study and have had the opportunity to consider the information, ask questions and have had these answered to my satisfaction.**	

For peer review only

For peer review only - http://bmjopen.bmj.com/site/about/guidelines.xhtml

**2. I understand that my participation in this study is voluntary and that I am free to withdraw at any time without giving a reason, without my medical care or legal rights being affected.**	
**3. I understand that sections of my medical notes and data collected during the study may be looked at by responsible individuals involved in this research at my hospital, the University of Liverpool, or from regulatory authorities where it is relevant to my taking part in research. I give permission for these individuals to have access to my records.**	

For peer review only - http://bmjopen.bmj.com/site/about/guidelines.xhtml

**4. I understand that imaging (Xray's, CT scans etc.) and blood test results will be reviewed by a central research team in order to assess success of the treatment I receive.**	
**5. I agree to allow my General Practitioner and any other relevant medical practitioner to be informed of my involvement in the study.**	

For peer review only

For peer review only - http://bmjopen.bmj.com/site/about/guidelines.xhtml

**6. I am aware that there is a qualitative aspect to this study and that patients may be selected at random to discuss their involvement and understanding of the study with a dedicated qualitative researcher. I agree to take part in these interviews if selected.**	
**7. I agree for a copy of this completed consent form to be sent to the University of Liverpool Clinical Trials Centre (where it will be kept in a secure location), to allow confirmation that my consent for the trial has been given.**	

For peer review only

For peer review only - http://bmjopen.bmj.com/site/about/guidelines.xhtml

**8. I understand that information held by the NHS and records maintained by the NHS Information Centre may be used to keep in touch with me and follow-up my health status.**		
**9. I agree that data from the study can be used in future research.**<br><br>10. I am aware that once registered on to the study there is no way to predict or influence which arm I am randomised to.		
11. I agree to take part in the LAP-PIE study		
**This part of the trial is OPTIONAL. Only initial YES if you wish to give permission. Initial NO if you do not give permission.**		
• I give permission for the samples of my blood/urine/tissue specimens to be taken and used for this trial and be transferred to research institutes in the UK and the rest of the world.	**Yes**	**No**
• I give permission for the samples of my blood/urine/tissue specimens to be used in future research and be transferred to research institutes in the UK and the rest of the world.	**Yes**	**No**
• I give permission for my stored pathological specimens to be used for this trial and be transferred to research institutes in the UK and the rest of the world.	**Yes**	**No**

For peer review only - http://bmjopen.bmj.com/site/about/guidelines.xhtml

• I give permission for my stored pathological specimens to be used for future research and be transferred to research institutes in the UK and the rest of the world.	**Yes**	**No**
• I give permission for genetic material (DNA and RNA) to be extracted from blood and cancer tissue specimens for current ethically approved research.	**Yes**	**No**
• I give permission for genetic material (DNA and RNA) to be extracted from blood and cancer tissue specimens for future ethically approved research.	**Yes**	**No**
• I agree that data from the study can be sent outside the European Economic Area (EEA).	**Yes**	**No**

**Name of patient**		**Date**		**Signature**
**Researcher**		**Date**		**Signature**
**Name of person taking consent (if different from researcher)**		**Date**		**Signature**

Please ensure the dates of consent are the same date for everyone, this form needs to be signed by both the patient and the person obtaining consent.
If there are any mistake made that need to be corrected, please draw a line through and write the correction next to it; initial and date the correction.
One copy of this form is given to the patient, one kept in the Patient File at the Research Site, and one copy is sent to the LCTC

For peer review only - http://bmjopen.bmj.com/site/about/guidelines.xhtml

PATIENT INFORMATION SHEET

Treatment of unresectable **L**ocally **A**dvanced **P**ancreatic cancer with **P**ercutaneous **I**rreversible **E**lectroporation (IRE) following initial systemic chemotherapy: a feasibility randomised controlled trial

You have been invited to take part in a research study. Before you decide, it is important that you understand why the research is being done and what it will involve. Please take time to read the following information carefully and discuss it with others if you wish.

- **Part One** tells you the purpose of the study and what will happen to you if you take part.
- **Part Two** gives you more detailed information about the conduct of the study.

The clinical team in change of your care will go through the details with you, but please ask us if there is anything that is not clear or if you would like more information. Take time to decide whether or not you wish to take part.
Thank you for reading this.

<u>Part One</u>

Please note that Locally Advanced Pancreatic Cancer has been shortened to LAPC throughout this information sheet.

What is the purpose of the study?

The Purpose of the LAP-PIE study is to see how well we are able to recruit patients to a study of this type, and to determine if a new type of cancer treatment is effective in reducing the size of a Pancreatic tumour to the point that it can be surgically removed. The treatment is known as Irreversible Electroporation (IRE), which essentially uses electric pulses to destroy the cancer cells whilst leaving the healthy cells unharmed. We are recruiting patients in order to see how effective this treatment option is compared to standard chemotherapy in patients, and at the same time look at issues in recruitment that will help us design a larger, more long-term study with the same goals.

Why have I been chosen?

You have been chosen as you are a patient who has been diagnosed with advanced pancreatic cancer that is not yet suitable for surgery. In addition, you are either due to start FOLFIRINOX® chemotherapy through the NHS, are currently having it, or at the end of treatment. You were approached by a member of the study team in a hospital that is currently running the LAP-PIE study and have been identified to meet some of our main inclusion criteria.

Do I have to take part?

For peer review only - http://bmjopen.bmj.com/site/about/guidelines.xhtml

No. It is up to you to decide whether or not to take part. If you do agree you will be asked to sign a consent form, but you are free to withdraw at any time afterwards without giving a reason. A decision to withdraw at any time, or a decision not to take part, will not affect the standard of care you receive.

What will happen to me if I take part?

From Randomisation, you will be on study for however long your second line chemotherapy lasts and a further 12 months for follow up.
The visits that you will attend are those that you would normally be required to make for treatment of LAPC; They may last slightly longer for some additional tests and for the research team to record the results of assessments for the study.
The results of standard of care tests such as blood, CT scans and biopsies will also be collected for the purposes of the study. All data is anonymised and kept on a secure system at the Liverpool Clinical Trials Centre.
If you are admitted to hospital for any reason during your time on the study, the details of the visit will be recorded and reviewed by the study team to ensure it is not related to the study itself.
At each visit you will be asked if you have experienced any adverse events or conditions or if you are taking any new medication since the last time you were seen.

What do I have to do?

If you become a patient on this study then you will be first asked to sign a consent form confirming you have read this form and spoken to the research team about the study and are willing to take part. Giving consent to the study means that you understand the assessments and visits necessary and are willing to adhere to the study protocol. You will then have some assessments done to confirm that you are eligible, many of them are tests you will already be having as a part of your standard care. You will have blood taken for laboratory assessment and will have an X-Ray of your chest and CT scan of your abdomen, pelvis and chest. All patients of childbearing potential will be given a test to confirm they are not pregnant before starting the study. There is also a quality of life questionnaire to complete which will ask you to record how you are feeling and describe your overall health.

Some patients will be selected for interviews with a qualitative researcher, which will be a discussion of your understanding of the study and how it was communicated to you. The interviewer will also ask you about your knowledge of the treatment available and your condition. This interview may be done over the phone, or in person during one of your visits to the hospital you are being treated at.

The results of these test will be sent to the LCTC who are running the study who will randomised you using a computer system to one of two arms, both arms are receiving the same standard of care that would be given, however one arm will receive on IRE procedure (more on this below).

For peer review only - http://bmjopen.bmj.com/site/about/guidelines.xhtml

Following randomisation, you will need to attend hospital to receive the therapy that your local oncologist recommends and adhere to all of the test and visits related to it. The choice of chemotherapy that you will be given is the same that would have been chosen regardless of whether you were on this study. Data on the tests and visits you attend will be recorded by the research team and given to the LCTC for the study results. Half of the patients randomised will also receive on IRE procedure at their recruiting hospital trust.

Patients receiving the IRE will be admitted to hospital for a few days during their second line chemotherapy where they will be given the IRE procedure. IRE will involve some pre procedure assessments and some after care in hospital. More details on the procedure are below.

At the end of chemotherapy, you will be reviewed to see if your LAPC is now suitable for surgery. If suitable you will be given the same procedure in hospital as normal for patients with LAPC.

Following the surgery and after caring you will need to attend hospital every 3 months for one year. At these visits you will have bloods taken, a physical exam and there will be X-Rays and CT scans done. You will also be given a quality of life questionnaire to complete at these visits.

Should you be unsuitable for IRE before you are meant to have it, or if your LAPC is not suitable for surgery after finishing chemotherapy, or your oncologist decides to take you off of the chemotherapy for whatever reason, you will still be given the option to remain on follow up for 12 months.

At the end of your involvement with the study, you will be given some final assessments and will not have to attend for any LAP-PIE related assessment, and no further data will be collected about you. Your involvement ends at the last follow up visit, or when you choose to not take part any further.

What is the Treatment that is being tested?

Irreversible Electroporation is a fairly new device, though has been licensed for use on the NHS and in patients with Locally Advanced Pancreatic Cancer. IRE is only routinely available on the NHS for patients with this disease at certain hospitals in the UK, such as the hospital you are attending.

IRE is delivered by inserting long needle probes through the skin and into the pancreas, the ends of them will surround the tumour. Once in place the generator on the IRE device will send electrical impulses down the needles and around the cancerous tissue. The pulses heat the cells and weaken their cell membranes, so they break down over around a 24-hour period following the treatment. IRE has been used previously in treating a number of types of cancer, such as Liver, Pancreatic and Prostate cancers on an experimental basis. IRE is used in order to reduce the size of a tumour so that it is

For peer review only - http://bmjopen.bmj.com/site/about/guidelines.xhtml

more suitable for surgical removal. It is different from other similar treatments in that is does not generate heat, which can cause damage to surrounding tissue.

The needles will be guided using a CT scan, and not open surgery, meaning there will be less recovery time. There will only be one 2-hour long procedure involving IRE for patients in this study. Before the procedure you will have a blood test and a further test 6 hours after your procedure ends. You will be admitted to hospital for a few nights for monitoring until deemed suitable for discharge, you will likely be prescribed pain killers to take as well. 6 days after your IRE treatment you will be asked to return for another blood test.

As IRE uses electricity, a heart monitor will be used to time each pulse, so it does not affect your hearts rhythm. Patients who have pacemakers or other indwelling electrical devices are therefore not suitable for this study.

All patients who take part in this study will still receive chemotherapy as normally indicated for their care, prior to receiving either chemotherapy plus IRE, or just more chemotherapy. It is not known at this time, whether the combination of IRE and Chemotherapy, or just Chemotherapy is more effective at reducing tumour size in LAPC to make it suitable for surgery. This study aims to look at how easy or difficult it is to recruit patients such as yourself and randomise to one option or the other before pancreatic surgery. The side effects, and overall quality of life of patients across the two groups will be compared too. The findings will guide a large study that will compare the two options with greater accuracy.

What are the alternatives for treatment?

Advanced Pancreatic Cancer that is not suitable for surgery is currently treated on the NHS using: Chemotherapy, Chemotherapy and Radiotherapy, or with Stereotactic body radiotherapy (essentially a more targeted, precise way of delivering radiotherapy). Each of these methods have their own levels of efficacy and side effects and given as needed depending on other patient factors such as overall health and the degree of Pancreatic Cancer.
All chemotherapy drugs have their own side effects, mostly nausea and fatigue as well as affecting your appetite. Radiotherapy is also known to carry these side effects and can also cause skin reactions in some cases. Some other devices are available that use direct heat to destroy cancerous tissue, however the temperatures are far greater than those used in IRE and can cause damage to the healthy surrounding tissue.

Your doctor will be able to give you more detail on these treatments.

What are the side effects of the treatment I will receive when taking part?

Patients who are enrolled to this study will receive, or have received, chemotherapy (Folfirinox®) for 6 weeks, before being randomised to Chemotherapy plus IRE, or just

For peer review only - http://bmjopen.bmj.com/site/about/guidelines.xhtml

chemotherapy. The side effects of the Folfirinox and whichever NICE approved chemotherapy given after will be well known and described to you by your oncologist.

The Side effects of the IRE based on early studies are:

- Some pain, though this can be managed with painkillers and will not normally last more than a few days
- Possible pancreatitis; this is an inflammation of the pancreas and can usually be managed with pain relief and you may need to spend a few days in hospital. If more severe this stay in hospital could extend to a few weeks or months, though this is not common
- Some problems with eating or drinking; you could have a diminished appetite or feel sick, or possibly experience heartburn. These symptoms do not normally last long
- A blood clot could develop in a vein near where the IRE was performed. This is rare and can be treated with medication, though would mean you may not be able to have surgery after the IRE, this will be for the doctor at site to decide. Standard practice with IRE is to administer drugs that reduce the risk of clots forming.
- In some very rare cases there may be some leaking of fluid from the bile duct or duodenum (the start of the small intestine). This can be caused by the needles that are used in IRE damaging these areas.

What are the possible disadvantages and risks of taking part?

This study is only recruiting patients who have been given Folfirinox as first line therapy and then their oncologist's choice for second line treatment. Therefore, the side effects and disadvantages of the chemotherapy aspect are the same as they would be regardless of entering the study. The potential side effects and risks associated with IRE are also listed above. It is possible that after undergoing either treatment option then your LAPC will still not be suitable for surgery. There is also always the possibility with any standard of care treatment that side effects, or complications, mean you will have to stop the therapy and another option will have to be considered.

There will also be the need to attend clinic on certain days that cannot be planned out at this stage, which will most likely be during working hours. There will also be more questions about your health and wellbeing asked at each routine visit in order to record the study data. Though most of the tests you will have are already part of standard of care, there will be some extra blood samples taken at various points in the study; all effort will be made to have these done at the same time as the routine bloods for your convenience.

What are the possible benefits of taking part?

We hope that the treatments will help you. However, this cannot be guaranteed. The information we get from this study may help us to improve the future treatment of patients with Advanced Pancreatic Cancer. The results of this feasibility study will also

For peer review only - http://bmjopen.bmj.com/site/about/guidelines.xhtml

For peer review only

help to develop a larger clinical trial into the use of IRE in patients with the same condition.

What happens when the research study stops?
It should be made clear if participants will have continued access to any benefits or interventions obtained during the research. If treatment is unavailable after the trial finishes, this should be explained, and information given on what treatment is available.

The chemotherapy that you will be given from this study are already approved and supplied via standard of care, and therefore will still be available should the study be terminated for any reason at your hospital.

What if there is a problem?
Any complaint about the way you have been dealt with during the study or any possible harm you might suffer will be addressed. The detailed information is given in Part 2 of this information sheet.

Will my taking part in this study be kept confidential?
Yes. All the confidential information about your participation in this study will be kept confidential. The detailed information on this is given in Part 2.

Contact for Further Information
Should you have any further queries regarding this study or about any of the treatments described above,

Please contact:
Appropriate job title:
[Trust to insert contact details]
Contact Number is:
[Trust to insert contact details]

This completes Part One of the Information Sheet. If the information in Part One has interested you and you are considering participation, please continue to read the additional information in Part Two before making any decision.

Part Two

What if new information becomes available?
Sometimes during the course of a research project, new information becomes available about the treatment/drug that is being studied. If this happens, your doctor will tell you about it and discuss with you whether you want to or should continue in the study. If you decide to withdraw your doctor will make arrangements for your care to continue. If you decide to continue in the study you will be asked to sign an updated consent form.

For peer review only - http://bmjopen.bmj.com/site/about/guidelines.xhtml

On receiving new information your doctor might consider it to be in your best interests to withdraw you from the study. He/she will explain the reasons and arrange for your care to continue.
If the study is stopped for any other reason you will be told why, and your continuing care will be arranged.

What will happen if I don't want to carry on with the study?

You retain the right to decide how much of the study you wish to participate in at all times. If there is any part of it you are not happy with you can choose not to have any test, give blood sample or answer any question. If you wish to no longer continue on the study this is your decision and will be respected. If you wish to stop during the chemotherapy phase then you will not be randomised to receive the IRE and may discuss with your doctor what you wish to do next. In all cases data and samples taken for patients who are withdrawn will be kept so they can still be used to help the study. At any time, you may tell the research team that you want any of your data and samples destroyed or not used in the research.

If you do not wish to attend the follow up visits this is entirely up to you. Every effort will be made to still contact you, or your GP for some data, or you may even decide that you no longer wish to be involved in the study at all.

What if there is a problem?
If you have a concern about any aspect of this study, you should ask to speak with the researchers who will do their best to answer your questions. If you remain unhappy and wish to complain formally, you can do this through the normal NHS Complaints Procedure. Details can be obtained from the hospital.
If you are harmed by taking part in this research project and this is due to someone's negligence, then you may have grounds for a legal action for compensation against the NHS Trust where you are being treated, but you may have to pay for your legal costs. The normal National Health Service complaints mechanisms should be available to you (if appropriate).

If you suffer harm arising from the negligent management or negligent design of the research you will have grounds to claim compensation. In the absence of negligence compensation is not payable.
If you experience non-negligent harm arising from the management or design of the research then The University of Liverpool clinical trials insurance may apply for this investigator-initiated study.
In the event of defective product then you may have grounds for a legal action for compensation against the manufacturer, but you may have to pay for your legal costs.

Will my taking part in this study be kept confidential?
The Royal Free Hospital is the sponsor for this study based in the United Kingdom. We will be using information from you and your medical records in order to undertake this study and will act as the data controller for this study. This means that we are

For peer review only - http://bmjopen.bmj.com/site/about/guidelines.xhtml

responsible for looking after your information and using it properly. [Sponsor organisation] will keep identifiable information about you [for x years after the study has finished/ until x].

Your rights to access, change or move your information are limited, as we need to manage your information in specific ways in order for the research to be reliable and accurate. If you withdraw from the study, we will keep the information about you that we have already obtained. To safeguard your rights, we will use the minimum personally identifiable information possible.

If you join the study, some parts of your medical records and the data collected for the study will be looked at by authorised persons from Cancer Research UK or their collaborators who are also involved in organising this research project and may be used in a number of research projects and/or for research related to the development of pharmaceutical products, diagnostics or medical aids by other scientists and research organisations within and outside the European Economic Area, in universities, the NHS or commercial companies involved in medical research worldwide which may also involve discussions with regulatory authorities. Patients' safety data may be reviewed outside the EU, but no one will be able to identify you. Data may also be looked at by representatives of regulatory authorities and by authorised people from the Trust, other NHS bodies or the commercial companies referred to above to check that the study is being carried out correctly. All will have a duty of confidentiality to you as a research participant.

A copy of your completed consent form will be sent to the Liverpool Cancer Trial Unit to allow them to check you have agreed to the trial. This will be kept in a secure location away from all the study data.

Involvement of the General Practitioner/Family Doctor (GP)
With your consent, your GP will be informed of your involvement in the trial. Any other medical practitioners who treat you, e.g. should you be admitted to hospital for any reason, will also be informed.

What will happen to any samples I give?
Some of your samples will be analysed at your hospital for the purpose of your treatment.
With your permission,. The researchers at the University of Liverpool work closely with other scientists in the UK and around Europe and, with your permission, your samples may be transferred to these research collaborators for use in future scientific studies. These samples will be used only for investigating Locally Advance Pancreatic Cancer and will not be used for any commercial purposes.
The samples will be kept in a secure place until we need them; nobody outside of the study will have access to **any** confidential information that you give to us. Confidential

For peer review only - http://bmjopen.bmj.com/site/about/guidelines.xhtml

details (such as your name, address and GP details) will be kept locally and not made available to collaborators.

Your sample will be coded and the researchers carrying out tests on the samples will not be given information they do not need to carry out the tests and analyse the results. Coded is not the same as anonymous. It will be possible to use the codes to identify that a result is from your sample. However, we do not plan to do this unless there is a good reason to do so. We will maintain this information so that we can properly manage the samples donated. For instance, sometimes we may need to update our record of your clinical details to help us interpret the results of tests.

What will happen to the results of the research study?

It is intended that once the study is complete a report will be written, and the results will be published to make them available to the public. They may also be used to apply to the regulatory authorities to make the drug widely available and/or for research related to the development of pharmaceutical products, diagnostics or medical aids. You will not be named or identified in any publication.

What rights do I have to the results of the research?

Any information derived directly or indirectly from this research, as well as any patents, diagnostic tests, drugs, or biological products developed directly or indirectly as a

result of this research may be used for commercial purposes. You have no right to this property or to any share of the profits that may be earned directly or indirectly as a result of this research. However, in signing this form and donating tissue and/or blood samples for this research, you do not give up any rights that you would otherwise have as a participant in research.

Who is organising and funding the research?

The study is funded by the NIHR and the equipment and training needed for IRE is being provided by Angiodynamics, the company who developed the technology.

Your doctor will not receive any payment for including you in this study.

The study is funded by the NIHR, the governing body that oversees and supports research done through the NHS. The money given a grant from them is to support the cost of performing the research such as the assessments, set up costs and time needed working on the study. No one involved in the research receives any payment for being involved, nor incentive to encourage recruitment to the LAP-PIE study.

Who has reviewed the study?

The study has been reviewed for scientific content by members of London Brent Research Ethics Committee (REC) has for ethical considerations and has a favourable opinion.

Thank you for taking the time to read and consider this information sheet. Should you decide to take part in the study, you will be given a copy of the information sheet and a signed consent form to keep.

For peer review only - http://bmjopen.bmj.com/site/about/guidelines.xhtml

For peer review only

For peer review only - http://bmjopen.bmj.com/site/about/guidelines.xhtml
